# A 2-million-year-old ecosystem in Greenland uncovered by environmental DNA

**DOI:** 10.1038/s41586-022-05453-y

**Published:** 2022-12-07

**Authors:** Kurt H. Kjær, Mikkel Winther Pedersen, Bianca De Sanctis, Binia De Cahsan, Thorfinn S. Korneliussen, Christian S. Michelsen, Karina K. Sand, Stanislav Jelavić, Anthony H. Ruter, Astrid M. A. Schmidt, Kristian K. Kjeldsen, Alexey S. Tesakov, Ian Snowball, John C. Gosse, Inger G. Alsos, Yucheng Wang, Christoph Dockter, Magnus Rasmussen, Morten E. Jørgensen, Birgitte Skadhauge, Ana Prohaska, Jeppe Å. Kristensen, Morten Bjerager, Morten E. Allentoft, Eric Coissac, PhyloNorway Consortium, Inger Greve Alsos, Eric Coissac, Alexandra Rouillard, Alexandra Simakova, Antonio Fernandez-Guerra, Chris Bowler, Marc Macias-Fauria, Lasse Vinner, John J. Welch, Alan J. Hidy, Martin Sikora, Matthew J. Collins, Richard Durbin, Nicolaj K. Larsen, Eske Willerslev

**Affiliations:** 1 Lundbeck Foundation GeoGenetics Centre, Globe Institute, University of Copenhagen, Copenhagen, Denmark; 2 Department of Zoology, University of Cambridge, Cambridge, UK; 3 Department of Genetics, University of Cambridge, Cambridge, UK; 4 Section for Molecular Ecology and Evolution, The Globe Institute, Faculty of Health and Medical Sciences, Copenhagen, Denmark; 5 Niels Bohr Institute, University of Copenhagen, Copenhagen, Denmark; 6 Université Grenoble Alpes, Université Savoie Mont Blanc, CNRS, IRD, Université Gustave Eiffel, ISTerre, Grenoble, France; 7 Nordic Foundation for Development and Ecology (NORDECO), Copenhagen, Denmark; 8 DIS Study Abroad in Scandinavia, University of Copenhagen, Copenhagen, Denmark; 9 Department of Glaciology and Climate, Geological Survey of Denmark and Greenland, Copenhagen, Denmark; 10 Geological Institute, Russian Academy of Sciences, Moscow, Russia; 11 Department of Earth Sciences, Uppsala University, Uppsala, Sweden; 12 Department of Earth and Environmental Sciences, Dalhousie University, Halifax, Nova Scotia Canada; 13 The Arctic University Museum of Norway, UiT—The Arctic University of Norway, Tromsø, Norway; 14 Carlsberg Research Laboratory, Copenhagen, Denmark; 15 Environmental Change Institute, School of Geography and the Environment, University of Oxford, Oxford, UK; 16 Geological Survey of Denmark and Greenland, (GEUS), Copenhagen, Denmark; 17 Department of Geophysics and Sedimentary Basins, Geological Survey of Denmark and Greenland, Copenhagen, Denmark; 18 Trace and Environmental DNA (TrEnD) Laboratory, School of Molecular and Life Sciences, Curtin University, Perth, Western Australia Australia; 19 University of Grenoble-Alpes, Université Savoie Mont Blanc, CNRS, LECA, Grenoble, France; 20 Institut de Biologie de l’Ecole Normale Supérieure (IBENS), Ecole Normale Supérieure, CNRS, INSERM Université PSL, Paris, France; 21 Department of Geosciences, UiT—The Arctic University of Norway, Tromsø, Norway; 22 School of Geography and the Environment, University of Oxford, Oxford, UK; 23 Center for Accelerator Mass Spectrometry, Lawrence Livermore National Laboratory, Livermore, CA USA; 24 Department of Archaeology, University of Cambridge, Cambridge, UK; 25 Section for GeoBiology, Globe Institute, University of Copenhagen, Copenhagen, Denmark; 26 MARUM, University of Bremen, Bremen, Germany

**Keywords:** Molecular ecology, Evolutionary ecology, Ecological genetics, Evolutionary ecology

## Abstract

Late Pliocene and Early Pleistocene epochs 3.6 to 0.8 million years ago^1^ had climates resembling those forecasted under future warming^2^. Palaeoclimatic records show strong polar amplification with mean annual temperatures of 11–19 °C above contemporary values^3,4^. The biological communities inhabiting the Arctic during this time remain poorly known because fossils are rare^5^. Here we report an ancient environmental DNA^6^ (eDNA) record describing the rich plant and animal assemblages of the Kap København Formation in North Greenland, dated to around two million years ago. The record shows an open boreal forest ecosystem with mixed vegetation of poplar, birch and thuja trees, as well as a variety of Arctic and boreal shrubs and herbs, many of which had not previously been detected at the site from macrofossil and pollen records. The DNA record confirms the presence of hare and mitochondrial DNA from animals including mastodons, reindeer, rodents and geese, all ancestral to their present-day and late Pleistocene relatives. The presence of marine species including horseshoe crab and green algae support a warmer climate than today. The reconstructed ecosystem has no modern analogue. The survival of such ancient eDNA probably relates to its binding to mineral surfaces. Our findings open new areas of genetic research, demonstrating that it is possible to track the ecology and evolution of biological communities from two million years ago using ancient eDNA.

## Abstract (web summary)

Analysis of two-million-year-old ancient environmental DNA from the Kap København Formation in North Greenland shows there was an open boreal forest with diverse plant and animal species, of which several taxa have not previously been detected at the site, representing an ecosystem that has no present-day analogue.

## Main

**Fig. 1:**
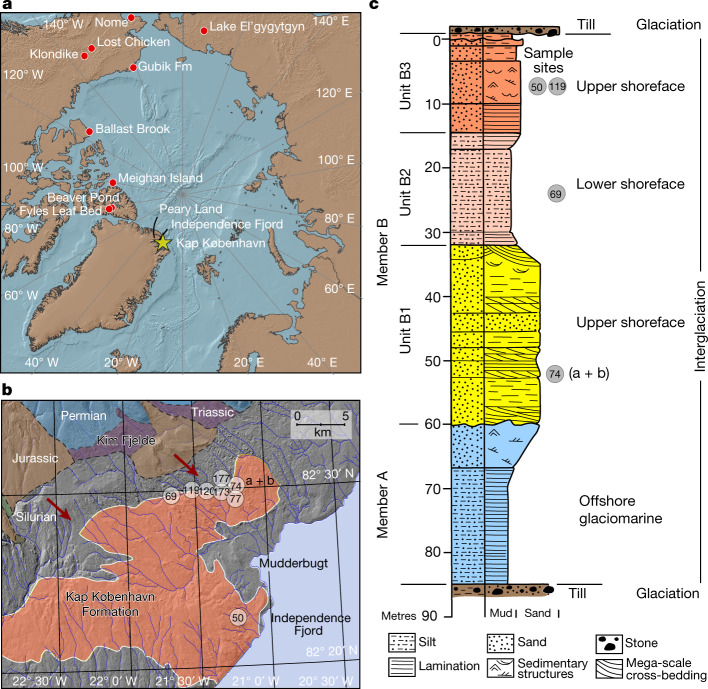
Geographical location and depositional sequence. **a**. Location of Kap København Formation in North Greenland at the entrance to the Independence Fjord (82° 24′ N 22° 12′ W) and locations of other Arctic Plio-Pleistocene fossil-bearing sites (red dots). **b**, Spatial distribution of the erosional remnants of the 100-m thick succession of shallow marine near-shore sediments between Mudderbugt and the low mountains towards the north (a + b refers to location 74a and 74b). **c**, Glacial–interglacial division of the depositional succession of clay Member A and units B1, B2 and B3 constituting sandy Member B. Sampling intervals for all sites are projected onto the sedimentary succession of locality 50. Sedimentological log modified after ref. ^7^. Circled numbers on the map mark sample sites for environmental DNA analyses, absolute burial dating and palaeomagnetism. Numbered sites refer to previous publications^7,10,11,14,61^.

The Kap København Formation is located in Peary Land, North Greenland (82° 24′ N 22° 12′ W) in what is now a polar desert. The upper depositional sequence contains well-preserved terrestrial animal and plant remains washed into an estuary during a warmer Early Pleistocene interglacial cycle^7^ (Fig. 1). Nearly 40 years of palaeoenvironmental and climate research at the site provide a unique perspective into a period when the site was situated at the boreal Arctic ecotone with reconstructed summer and winter average minimum temperatures of 10 °C and −17 °C respectively—more than 10 °C warmer than the present^7–11^. These conditions must have driven substantial ablation of the Greenland Ice Sheet, possibly producing one of the last ice-free intervals^7^ in the last 2.4 million years (Myr). Although the Kap København Formation is known to yield well-preserved macrofossils from a coniferous boreal forest and a rich insect fauna, few traces of vertebrates have been found. To date, these comprise remains from lagomorph genera, their coprolites and *Aphodius* beetles, which live in and on mammalian dung^10,11^. However, the approximately 3.4 Myr old Fyles Leaf bed and Beaver Pond on Ellesmere Island in Arctic Canada preserve fossils of mammals that potentially could have colonized Greenland, such as the extinct bear (*Protarctos abstrusus*), extinct beavers (*Dipoides* sp.), the small canine *Eucyon* and Arctic giant camelines^4,12,13^ (similar to *Paracamelus*). Whether the Nares Strait was a sufficient barrier to isolate northern Greenland from colonization by this fauna remains an open question.

**Extended Data Fig. 1:**
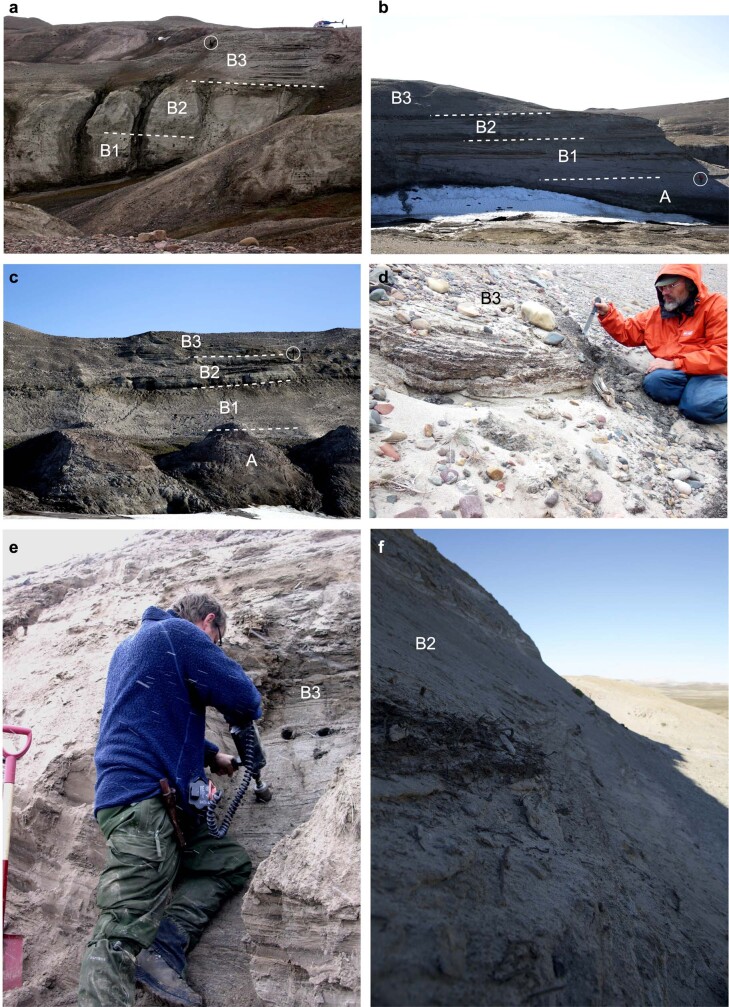
Setting A. Type locality 50 indicating units in formation **b**. Overview locality 74a+b with a complete sediment sequence. **C**. Overview of locality 69. **D**. Detail of organic rich sediment in unit B3 before excavation and cleaning for ancient eDNA samples. **E**. Sampling in the permafrost within unit B3 at locality 50. **F**. Organic rich sediment at the base of mega-scale cross-bedding within unit B2 at locality 74a+b. White circles mark persons for scale.

The Kap København Formation is formally subdivided into two members^7^ (Fig. 1). The lower Member A consists of up to 50 m of laminated mud with an Arctic ostracod, foraminifera and mollusc fauna deposited in an offshore glaciomarine environment^14^. The overlying Member B consists of 40–50 m of sandy (units B1 and B3) and silty (unit B2) deposits (Extended Data Fig. 1), including thin organic-rich beds with an interglacial macrofossil fauna that were deposited closer to the shore in a shallow marine or estuarine environment represented by upper and lower shoreface sedimentary facies^7^.

The specific depositional environments are also reflected in the mineralogy of the units, where the proximal B3 locality has the lowest clay and highest quartz contents (Sample compositions in Supplementary Tables 4.2.1 and 4.2.2 and unit averages in Supplementary Tables 4.2.3 and 4.2.4). The architecture of the basin infill suggests that Member B units thicken towards the present coast—that is, distal to the sediment source in the low mountains in the north (Fig. 1). Abundant organic detritus horizons are recorded in units B1 and B3, which also contain beds rich in Arctic and boreal plant and invertebrate macrofossils, as well as terrestrial mosses^10,15^. Therefore, the taphonomy of the DNA most probably reflects the biological communities eroded from a range of habitats, fluvially transported to the foreshore and concentrated as organic detritus mixed into sandy near-shore sediments within units B1 and B3. Conversely, the deeper water facies from Member A and unit B2 have a stronger marine signal. This scenario is supported by the similarities in the mineralogic composition between Kap København Formation sediments and Kim Fjelde sediments (Supplementary Tables 4.2.1 and 4.2.5).

## Geological age

**Fig. 2:**
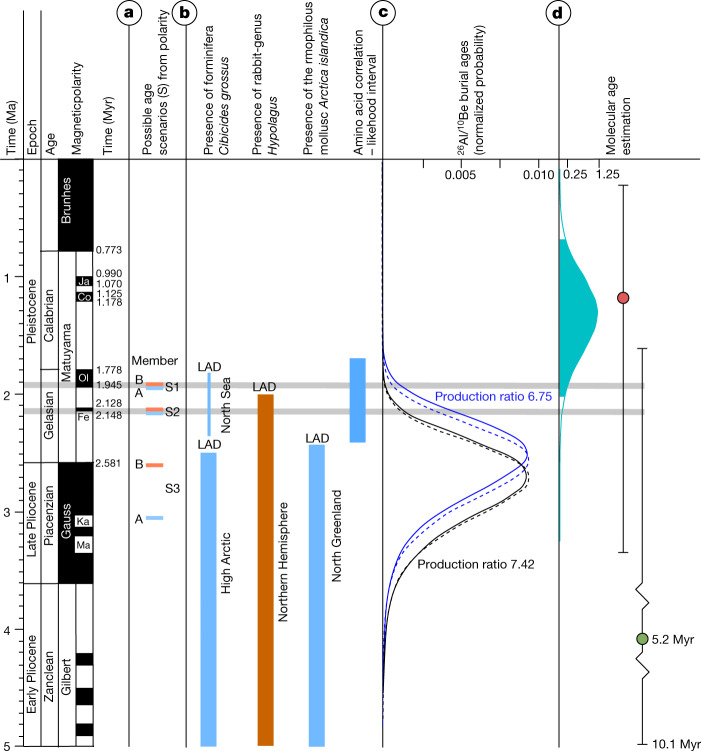
Age proxies for the Kap København Formation. **a**, Revised palaeomagnetic analysis shows unit B2 to have normal polarity and unlocks three possible age scenarios (S1–S3) including Members A (blue) and B (brown). Normal polarity is coloured black and reverse polarity is shown in white. Ja, Jaramillo; Co, Cobb Mountain; Ol, Olduvai; Fe, Feni; Ka, Kaena; Ma, Mammoth. **b**, Presence and last appearance datum (LAD) for marine foraminifera *Cibicides grossus*, rabbit-genus *Hypolagus* and the mollusc *Arctica islandica* in the High Arctic, Northern Hemisphere and North Greenland, respectively. The blue band on the far right indicates the age range for Member A estimated from amino acid ratios on shells^7^. **c**, Convolved probability distribution functions for cosmogenic burial ages calculated for two different production ratios (7.42 (black) and 6.75 (blue)). The dashed line and the solid line show the distributions for steady erosion and zero erosion, respectively. These distributions are all maximum ages. **d**, Molecular dating of *Betula* sp. yielding a median age of the DNA in the sediment of 1.323 Myr, with whiskers confining the 95% height posterior density (HPD) of 0.68 to 2.02 Myr (blue density plot), running Markov chain Monte Carlo estimation for 100 million iterations. The red dot is the median molecular age estimate found using the Mastodon mitochondrial genome restricting to radiocarbon-dated specimens, whereas the green area includes molecular clock estimated specimens in BEAST, running Markov chain Monte Carlo estimation for 400 million iterations. Whiskers confine the 95% HPD.

A series of complementary studies has successively narrowed the depositional age bracket of the Kap København Formation from 4.0–0.7 Myr to a 20,000-year-long age bracket around 2.4 Myr (see Supplementary Information, sections 1–3). This was achieved by a combination of palaeomagnetism, biostratigraphy and allostratigraphy^7,14,16–18^. Notably, the last appearance data of the mammals, foraminifera and molluscs in the stratigraphic record show an age close to 2.4 Myr (see Supplementary Information, section 2). Within this overall framework, we add new palaeomagnetic data showing that Member A has reversed magnetic polarity and the main part of the overlying unit B2 has normal magnetic polarity. In the context of previous work, this is consistent with three magnetostratigraphic intervals in the Early Pleistocene where there is a reversal: 1.93 Myr (scenario 1), 2.14 Myr (scenario 2) or 2.58 Myr (scenario 3) (Supplementary Information, section 1). Furthermore, we constrain the age using cosmogenic ^26^Al:^10^Be burial dating of Member B at four sites in this study (Supplementary Information, section 3). The recommended maximum burial age for the Kap København Formation is 2.70 ± 0.46 Myr (Fig. 2; Methods). However, we discard the older scenario 3 as it contradicts the evidence for a continuous sedimentation across Members A and B during a single glacial–interglacial depositional cycle^7,14,16,18,19^. This leaves two possible scenarios (scenarios 1 and 2), in which scenario 1 supports an age of 1.9 Myr and scenario 2 supports an age of 2.1 Myr.

## DNA preservation

DNA degrades with time owing to microbial enzymatic activity, mechanical shearing and spontaneous chemical reactions such as hydrolysis and oxidation^20^. The oldest known DNA obtained to date has been recovered from a permafrost-preserved mammoth molar dated to 1.2–1.1 Myr using geological methods and 1.7 Myr (95% highest posterior density, 2.1–1.3 Myr) using molecular clock dating^21^. To explore the likelihood of recovering DNA from sediments at the Kap København formation, we calculated the thermal age of the DNA and its expected degree of depurination at the Kap København Formation. Using the mean average temperature^22^ (MAT) of −17 °C, we found a thermal age of 2.7 thousand years for DNA at a constant 10 °C, which is 741 times less than the age of 2.0 Myr (Supplementary Information, section 4 and Supplementary Table 4.4.1). Using the rate of depurination from Moa bird fossils^23^, we found it plausible that DNA with an average size of 50 base pairs (bp) could survive at the Kap København Formation, assuming that the site remained frozen (Supplementary Information, section 4 and Supplementary Table 4.4.2). Mechanisms that preserve DNA in sediments are likely to be different from that of bone. Adsorption at mineral surfaces modifies the DNA conformation, probably impeding molecular recognition by enzymes, which effectively hinders enzymatic degradation^24–27^. To investigate whether the minerals found in Kap København Formation could have retained DNA during the deposition and preserved it, we determined the mineralogic composition of the sediments using X-ray diffraction and measured their adsorption capacities. Our findings highlight that the marine depositional environment favours adsorption of extracellular DNA on the mineral surfaces (Supplementary Information, section 4 and Supplementary Table 4.3.1.1). Specifically, the clay minerals (9.6–5.5 wt%) and particularly smectite (1.2–3.7 wt%), have higher adsorption capacity compared to the non-clay minerals (59–75 wt%). At a DNA concentration representative of the natural environments^28^ (4.9 ng ml^−1^ DNA), the DNA adsorption capacity of smectite is 200 times greater than for quartz. We applied a sedimentary eDNA extraction protocol^29^ on our mineral-adsorbed DNA samples, and retrieved only 5% of the adsorbed DNA from smectite and around 10% from the other clay minerals (Methods and Supplementary Information, section 4). By contrast, we retrieved around 40% of the DNA adsorbed to quartz. The difference in adsorption capacity and extraction yield from the different minerals demonstrates that mineral composition may have an important role in ancient eDNA preservation and retrieval.

## Kap København metagenomes

**Fig. 3:**
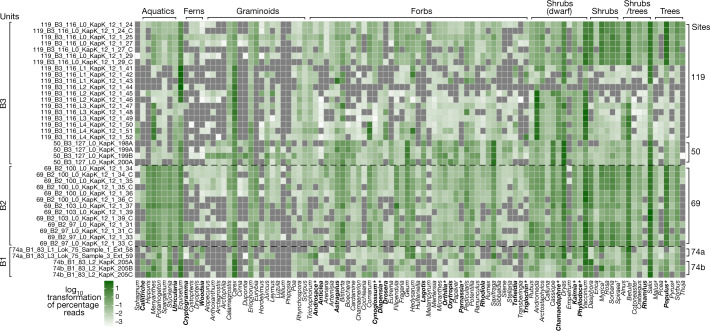
Early Pleistocene plants of northern Greenland. Taxonomic profiles of the plant assemblage found in the metagenomes. Taxa in bold are genera only found as DNA and not as macrofossil or pollen. Asterisks indicate those that are found at other Pliocene Arctic sites. Extinct species as identified by either macrofossils or phylogenetic placements are marked with a dagger. Reads classified as *Pyrus* and *Malus* are marked with a pound symbol, and are probably over-classified DNA sequences belonging to another species within Rosaceae that are not present as a reference genome.

**Fig. 4:**
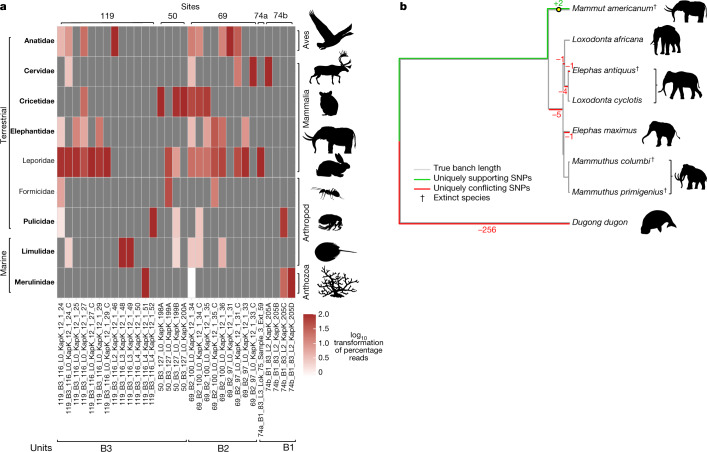
Early Pleistocene animals of northern Greenland. **a**, Taxonomic profiles of the animal assemblage from units B1, B2 and B3. Taxa in bold are genera only found as DNA. **b**, Phylogenetic placement and pathPhynder^62^ results of mitochondrial reads uniquely classified to Elephantidae or lower (Source Data 1). Extinct species as identified by either macrofossils or phylogenetic placements are marked with a dagger.

We extracted DNA^29^ from 41 organic-rich sediment samples at five different sites within the Kap København Formation (Supplementary Information, section 6 and Source Data 1), which were converted into 65 dual-indexed Illumina sequencing libraries^30^. First, we tested 34 of the 65 libraries for plant plastid DNA by screening for the conserved photosystem II D2 (*psbD*) gene using droplet digital PCR (ddPCR) with a gene-targeting primer and probe spanning a 39-bp region and a P7 index primer. Further, we screened for the *psbA* gene using a similar assay targeting the Poaceae (Methods and Supplementary Fig. 6.12.1). A clear signal in 31 out of 34 samples tested confirmed the presence of plant plastid DNA in these libraries (Source Data 1, sheets 5 and 6). Additionally, we subjected 34 of the 65 libraries to mammalian mtDNA capture enrichment using the Arctic PaleoChip 1.0^31^ and shotgun sequenced all libraries (initial and captured) using the Illumina HiSeq 4000 and NovaSeq 6000. A total of 16,882,114,068 reads were sequenced, which after adaptor trimming, filtering for ≥30 bp and a minimum phred quality of 30 and duplicate removal resulted in 2,873,998,429 reads. These were analysed for *k-*mer comparisons using simka^32^ (Supplementary Information, section 6) and then parsed for taxonomic classification using competitive mapping with HOLI (https://github.com/miwipe/KapCopenhagen.git), which includes a recently published dataset of more than 1,500 genome skims of Arctic and boreal plant taxa^33,34^ (Methods and Supplementary Information, section 6). Considering the age of the samples and thus the potential genetic distance to recent reference genomes, we allowed each read to have a similarity between 95–100% for it to be taxonomically classified using ngsLCA^35^. The metaDMG (v.0.14.0) program^36^ was subsequently used to quantify and filter each taxonomic node for postmortem DNA damage for all the metagenomic samples (Methods). This method estimates the average damage at the termini position (D-max) and a likelihood ratio (λ**-**LR) that quantifies how much better the damage model (that is, more damage at the beginning of the read) fits the data compared with a null model (that is, a constant amount of damage; see Supplementary Information, section 6). We found the DNA damage to be highly increased, especially for eukaryotes (mean D-max = 40.7%, see Supplementary Information, section 6). From this we set D-max ≥25% as a filtering threshold for a taxonomic node to be parsed for further downstream analysis as well as a λ**-**LR higher or equal to 1.5. We furthermore set a threshold requiring that the minimum number of reads per taxon exceeded the median of reads assigned across all taxa divided by two to filter for taxa in low abundance. Similarly, for a sample to be considered, the total number of reads for a sample had to exceed the median number of reads per sample divided by two, to filter for samples with fewest reads. Lastly, we filtered out taxa with fewer than three replicates and subsequently reads were normalized by conversion to proportions (Figs. 3 and 4a).

## DNA, pollen and macrofossils comparison

Greenland’s coasts extend from around 60° to 83° N and include bioclimatic zones from the subarctic to the northern polar desert^37,38^. There are 175 vascular plant genera native to Greenland, excluding historically introduced species^39–41^. Of these, 70 (40%) were detected by the metagenomic analysis (Fig. 3); the majority of these genera are today confined to bioclimatic zones well to the south of Kap København’s polar desert (see ref. ^42^ and references therein), for example, all aquatic macrophytes. Reads assigned to *Salix*, *Dryas*, *Vaccinium*, *Betula*, *Carex* and *Equisetum* dominate the assemblage, and of these genera, *Equisetum*, *Dryas*, *Salix arctica* and two species of *Carex* (*Carex nardina* and *Carex stans*) grow there currently, whereas only a few records of *Vaccinium uliginosum* are found above 80º N, and *Betula nana* are found above 74º N (ref. ^43^). Out of the 102 genera detected in the Kap København ancient eDNA assemblage, 39% no longer grow in Greenland but do occur in the North American boreal (for example, *Picea* and *Populus*) and northern deciduous and maritime forests (for example, *Crataegus*, *Taxus*, *Thuja* and *Filipendula*). Many of the plant genera in this diverse assemblage do not occur on permafrost substrates and require higher temperatures than those at any latitude on Greenland today.

In addition to the DNA, we counted pollen in six samples from locality 119, unit B3 (Methods and Supplementary Fig. 5.1.1). Percentages were calculated for 4 of the samples with pollen sums ranging from 71–225 terrestrial grains (mean = 170.25). Upland herbs, including taxa in the Cyperaceae, Ericales and Rosaceae comprised around 40% of sample 4. Samples 5 and 6 were dominated by arboreal taxa, particularly *Betula*. The Polypodiopsida (for example, *Equisetum*, *Asplenium* and *Athyrium filix-mas*) and Lycopodiopsida (*Lycopodium annotinum* and *Selaginella rupestris*) were also well represented and comprised over 30% of the assemblage in samples 1, 4 and 6.

A total of 39 plant genera out of the 102 identified by DNA also occurred as macrofossils or pollen at the genus level. A further 39 taxa were potentially identified as macrofossil or pollen but not to the same taxonomic level^10,15^ (Source Data 1, sheets 1 and 2). For example, 12 genera of Poaceae were identified by DNA (*Alopecurus*, *Anthoxanthum*, *Arctagrostis*, *Arctophila*, *Calamagrostis*, *Cinna*, *Dupontia*, *Hordelymus*, *Leymus*, *Milium*, *Phippsia* and *Poa*), of these only *Hordelymus* is not found in the Arctic today (http://panarcticflora.org/), but these were only distinguished to family level in the pollen analysis and only one Poaceae macrofossil was found. There were 24 taxa that were recorded only as DNA. These included the boreal tree *Populus* and a few shrubs and dwarf shrubs, but mainly herbaceous plants. Of the 73 plant genera recovered as macrofossils^10,15^, only 24 were not detected in the DNA analysis. Because macrofossils and DNA have similar taphonomies—as both are deposited locally—more overlap is expected between them than between DNA and pollen, which is typically dispersed regionally^44^. Nine of the taxa absent in DNA were bryophytes, probably owing to poor representation of this group within the genomic reference databases. Furthermore, the extinct taxon Araceae is not present in the reference databases. The remaining undetected genera were vascular plants, and all except two (*Oxyria* and *Cornus*) were rare in the macrofossil record. Because the detection of rare taxa is challenging in both macrofossil and DNA records^45^, we argue that this overlap between the DNA and macrofossil records is as high as can be expected on the basis of the limitations of both methods.

An additional 19 taxa were recorded in the pollen record presented here and in that of Bennike^46^ including four trees or shrubs, five ferns, three club mosses, and one each of algae, fungi and liverwort. We also find pollen from anemophilous trees, particularly gymnosperms, which can be distributed far north of the region where the plants actually grow^10^. Bennike^46^ also notes a high proportion of club mosses and ferns and suggests they may be overrepresented owing to their spore wall being resistant to degradation. Furthermore, if these taxa were preferentially distributed along streams flowing into the estuary, their spores could be relatively more concentrated in the alluvium than the pollen of more generally distributed taxa. Thus, both decay resistance and alluvial deposition could contribute to the relative frequencies we observe. This same alluvial dynamic might also have contributed to the very large read counts for *Salix*, *Betula*, *Populus*, *Carex* and *Equisetum* in the metagenomic record, implying that neither the proportion of these taxa in the pollen records nor read counts necessarily correlate with their actual abundance in the regional vegetation in terms of biomass or coverage.

Finally, we sought to date the age of the plant DNA by phylogenetic placement of the chloroplast DNA. We examined data for the genera *Betula*, *Populus* and *Salix*, because these had both sufficiently high chloroplast genome coverage (with mean depth 24.16×, 57.06× and 27.04×, respectively) and sufficient present-day whole chloroplast reference sequences (Methods). Owing to their age and hence potential genetic distance from the modern reference genomes, we lowered the similarity threshold of uniquely classified reads to 90% and merged these by unit to increase coverage. Both *Betula* and *Salix* placed basally to most of the represented species in the respective genera, and the *Populus* placement results showed support for a mixture of different species related to *P. trichocarpa* and *P. balsamifera* (Extended Data Figs. 7–9).

We used the *Betula* chloroplast reads for a molecular dating analysis, because they were placed confidently on a single edge of the phylogenetic tree (that is, not a mixture as in *Populus*), had a large number of reference sequences, and had high coverage in the ancient sample. We used BEAST^47^ v1.10.4 to obtain a molecular clock date estimate for our ancient *Betula* chloroplast sample (see Methods, ‘Molecular dating methods’ for details). We included 31 modern *Betula* and one *Alnus* chloroplast reference sequences, used only sites that had a depth of at least 20 in the ancient sample, and included a previously estimated *Betula–Alnus* chloroplast divergence time^48^ of 61.1 Myr for calibration of the root node. Our BEAST analysis was robust to both different priors on the age of the ancient sample, and to different nucleotide substitution models (Extended Data Fig. 10). This yielded a median age estimate of 1.323 Myr, with a 95% HPD of (0.6786, 2.0172) Myr (Fig. 2).

## Animal DNA results

The metazoan mitochondrial and nuclear DNA record was much less diverse than that of the plants but contained one extinct family, one that is absent from Greenland today, and four vertebrate genera native to Greenland as well as representatives of four invertebrate families (Fig. 4a). Assignments were based on incomplete and variable representation of reference genomes, so we identified reads to family level, and only where sufficient mitochondrial reads were present, we refined the assignment to genus level by matching these into mitochondrial phylogenies based on more complete present-day mitochondrial sequences (Supplementary Information, section 6). As for the plant reads, uniquely classified animal reads with more than 90% similarity were parsed and merged by unit to increase coverage for phylogenetic placement.

Most notably, we found reads in unit B2 and B3 assigned to the family Elephantidae, which includes elephants and mammoths, but taxonomically not mastodon (*Mammut* sp.)—which are, however, in the NCBI taxonomy, and therefore our analysis reads classified to Elephantidae or below therefore include *Mammut* sp. A consensus genome of our Elephantidae mitochondrial reads falls on the *Mammut* sp. branch (Fig. 4b) and is placed basal to all clades of mastodons. However, we note that this placement within the mastodons depends on only two transition single-nucleotide polymorphisms (SNPs), with the first one supported by a read depth of three and the second by only one (Extended Data Fig. 4, Methods and Supplementary Information, section 6). Furthermore, we attempted dating the recovered mastodon mitochondrial genome using BEAST^49^. We implemented two dating approaches, one was based on using radiocarbon-dated specimens alone, while the other used radiocarbon- and molecular-dated mastodons. The first analysis yielded a median age estimate for our mastodon mitogenome of 1.2 Myr (95% HPD: 191,000 yr–3.27 Myr), the second approach resulted in a median age estimate of 5.2 Myr (95% HPD: 1.64–10.1 Myr) (Supplementary Fig. 6.8.5 and Supplementary Information, section 6).

**Extended Data Fig. 2:**
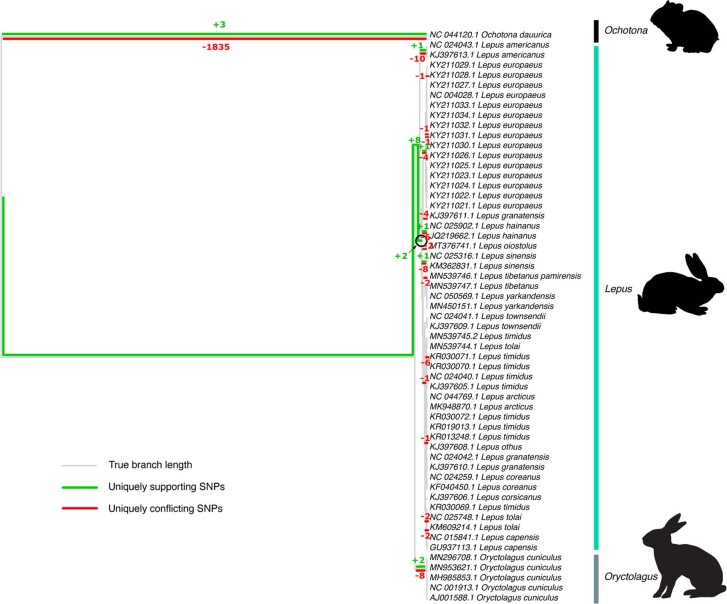
Phylogenetic placement results of Leporidae mitochondrial reads, using transversion SNPs only. Reads have been merged from all layers and sites. The green numbers on each edge represent the number of supporting (+) SNPs, whereas the red numbers indicate conflicting (−) SNPs in the ancient Leporidae environmental mitochondrial genome overlapping the reference SNPs assigned to the respective edge. There is a clear placement for the ancient Leporidae environmental mitochondrial genome on the edge marked +2, basal to the extant *Lepus* lineage.

**Extended Data Fig. 3:**
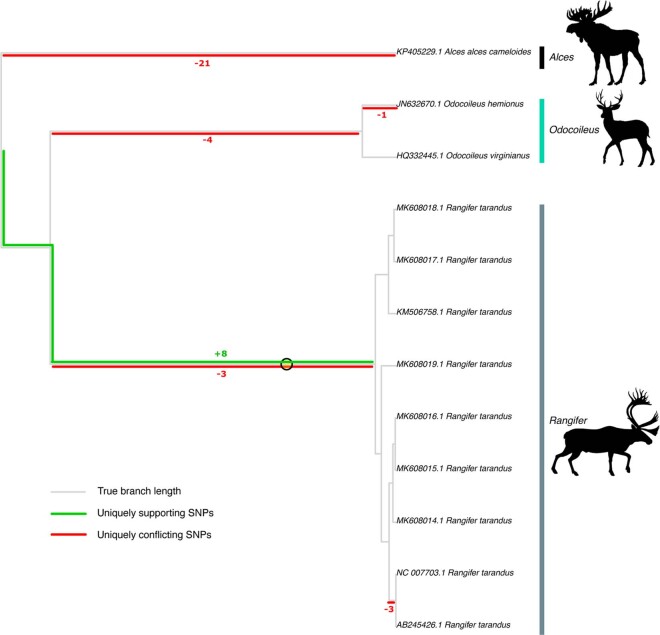
Phylogenetic placement results for representatives of the Capreolinae mitochondrial reads, using transversion SNPs only. Reads have been merged from all layers and sites. The green numbers on each edge represent the number of supporting (+) SNPs, whereas the red numbers indicate conflicting (−) SNPs in the ancient Capreolinae environmental mitochondrial genome overlapping the reference SNPs assigned to the respective edge. There is a clear placement for the ancient Capreolinae environmental mitochondrial genome on the edge marked +8/−3, basal to the *Rangifer* genus.

**Extended Data Fig. 4:**
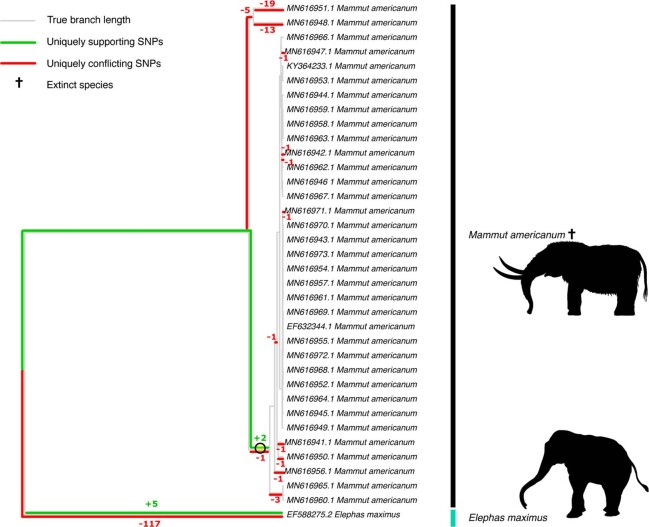
Phylogenetic placement of Elephantidae mitochondrial reads within mastodons (*Mammut americanum*), using *Elephas maximus* as outgroup, including transitions and transversion SNPs. (Please note that the NCBI taxonomy includes the Mammut genus within Elephantidae). The reference dataset consisted of mitochondria from mastodons (*Mammut americanum*) only and one *Elephas maximus* as an outgroup. Reads have been merged from all layers and sites. The green numbers on each edge represent the number of supporting (+) SNPs, whereas the red numbers indicate conflicting (−) SNPs in the ancient Elephantidae environmental mitochondrial genome overlapping the reference SNPs assigned to the respective edge. There is a placement for the ancient Elephantidae environmental mitochondrial genome on the edge marked +2/−1, identifying it as basal to the mastodon (*Mammut americanum*) clade, which contains most of all mastodon reference mitochondrial genomes. Please note that this placement is based on two transition SNPs with a read depth of three reads per SNP.

**Extended Data Fig. 5:**
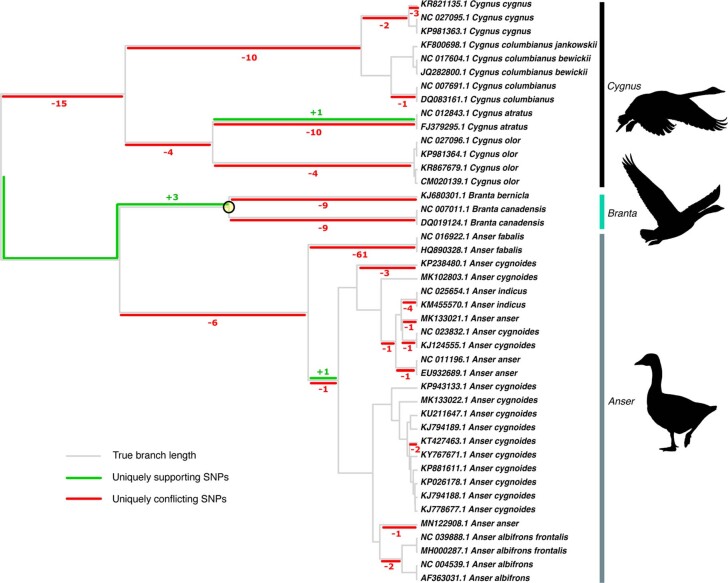
Phylogenetic placement of mitochondrial reads assigned within Anatidae and placed with representatives of the Anatidae, using transversion SNPs only. Reads have been merged from all layers and sites. The green numbers on each edge represent the number of supporting (+) SNPs, whereas the red numbers indicate conflicting (−) SNPs in the ancient Anatidae environmental mitochondrial genome overlapping the reference SNPs assigned to the respective edge. There is a clear placement for the ancient Anatidae environmental mitochondrial genome on the edge marked +3, basal to the *Branta* genus.

**Extended Data Fig. 6:**
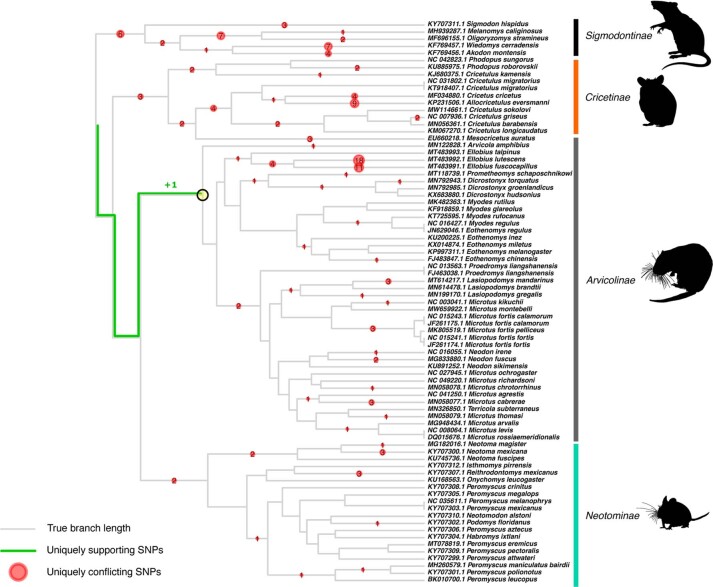
Phylogenetic placement results of Cricetidae mitochondrial reads, using transversion SNPs only. Reads have been merged from all layers and sites. The green numbers on each edge represent the number of supporting (+) SNPs, whereas the red numbers in the red circles indicate conflicting (−) SNPs in the ancient Cricetidae environmental mitochondrial genome overlapping the reference SNPs assigned to the respective edge. There is a placement for the ancient Cricetidae environmental mitochondrial genome on the edge marked +1, basal to the Arvicolinae subfamily.

**Extended Data Fig. 7:**
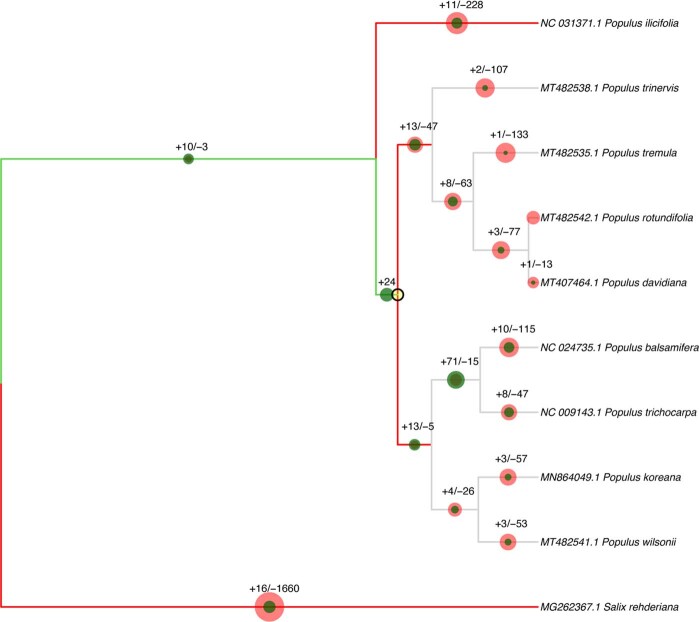
Phylogenetic placement results for our *Populus* chloroplast reads, using both transition and transversion SNPs, and using reads merged from all layers and sites. The numbers on each edge represent the number of supporting (+) and conflicting (−) SNPs in the ancient *Populus* environmental genome overlapping the reference SNPs assigned to that edge. The ancient *Populus* environmental genome clearly contains a mixture of different species. The most likely placement is on the edge above *Populus trichocarpa* (NC 009143.1) and *Populus balsamifera* (NC 024735.1), with +71/−15 supporting and conflicting SNPs. However, we find some support for both branches directly leading to these species as well. *Populus balsamifera* and *P. trichocarpa* are considered sister species. They are both distributed in North America, as far North as Alaska, are known to hybridise both among themselves and other *Populus* species and are morphologically very similar^93,99,100^. Previous analyses found a very recent nuclear genome divergence time of only 75000 years ago for *Populus trichocarpa* and *P. balsamifera*^100^, but a deep chloroplast genome divergence time of at least 6-7 Ma^99^, which is an uncommon pattern in plants. Our ancient *Populus* sample could contain individuals either ancestral to, or hybridized from, the modern *Populus trichocarpa* and *P. balsamifera* species.

**Extended Data Fig. 8:**
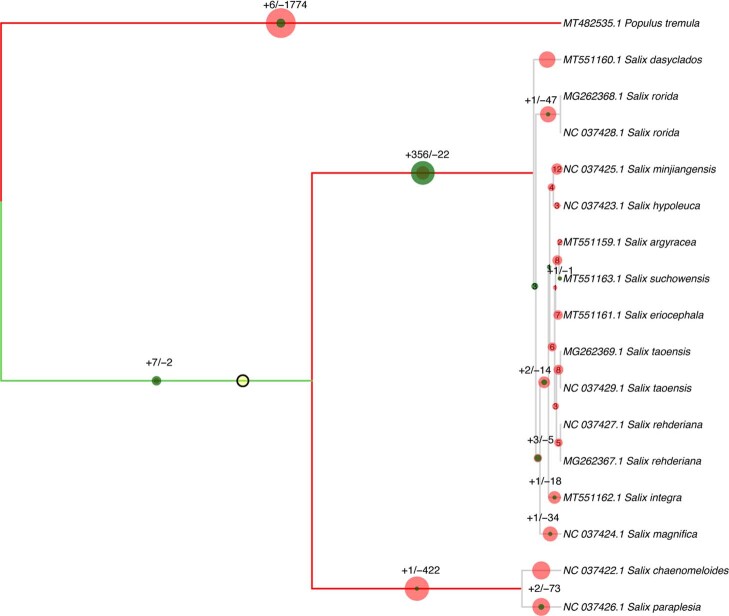
Phylogenetic placement results for our *Salix* chloroplast reads, using both transition and transversion SNPs, and using reads merged from all layers and sites. The numbers on each edge represent the number of supporting (+) and conflicting (−) SNPs in the ancient *Salix* environmental genome overlapping the reference SNPs assigned to that edge. The ancient *Salix* environmental genome falls basal to a main *Salix* clade. Our ancient *Salix* sample is phylogenetically placed, with 356 supporting SNPs and 22 conflicting SNPs, on a basal branch leading to the main clade. Although the *Salix* chloroplast phylogeny is not considered fully resolved^93^, the difficulties in resolution lie underneath our placement branch, and this along with the high number of SNPs on the placement branch allow us to be confident in the placement position. Our chloroplast phylogeny agrees roughly with^101^, which estimated a divergence date between these two main *Salix* clades at 16.9 Ma, and a root age of the first clade, to which our ancient sample is basal to, of 8.1 Ma. It is reasonable, then, to conclude that our ancient *Salix* sample is at least 8.1 Ma diverged from modern *Salix* species, and probably represents an extinct species, or extant species without a reference genome sequenced, or a pool thereof.

Similarly, reads assigned to the Cervidae support a basal placement on the *Rangifer* (reindeer and caribou) branch (Extended Data Fig. 3). Mitochondrial reads mapping to Leporidae (hares and rabbits) place near the base to the Eurasian hare clade (Extended Data Fig. 2), which is the only mammal found in the fossil record^7^. *Lepus*, specifically *Lepus arcticus*, is also the only genus in the Leporidae living in Greenland today. Mitochondrial reads assigned to Cricetidae cover only one informative transversion SNP, which places them as deriving from the subfamily Arvicolinae (voles, lemmings and muskrats) (Extended Data Fig. 6). For the only avian taxon represented in our dataset—Anatidae, the family of geese and swans—we found a robust basal placement to the genus *Branta* of black geese, supported by three transversion SNPs with read depths ranging between two and four (Extended Data Fig. 5). The refined vertebrate assignments based on mitochondrial references are more biogeographically conserved than for plants. *Dicrostonyx*—specifically *Dicrostonyx groenlandicus* (the Nearctic collared lemming)—is the only genus of the Cricetidae native to Greenland today, just as *Rangifer*—specifically *Rangifer tarandus groenlandicus* (the barren-ground caribou)—is the only member of the Cervidae. The mastodon is the exception, as no member of the Elephantidae lives in present-day Greenland.

## Ancient DNA from marine organisms

The other metazoan taxa identified in the DNA record were a single reef-building coral (Merulinidae) and several arthropods, with matches to two insects—Formicidae (ants) and Pulicidae (fleas)—and one marine family—Limulidae (horseshoe crabs). This is somewhat unexpected, given the rich insect macrofossil record from the Kap København Formation, which comprises more than 200 species, including *Formica* sp. The marine taxa are less abundant than the terrestrial taxa, and no mitochondrial DNA was identified from marine metazoans. The read lengths, DNA damage and the fact that the reads assigned distribute evenly across the reference genomes suggests that these are not artefacts but may be over-matched DNA sequences of closely related, potentially extinct species within the families that are currently absent from our reference databases owing to poor taxonomic representation. By contrast, Limulidae, in the subphylum *Chelicerata*, is unlikely to be misidentified as this distinct genus is the only surviving member within its order and thus deeply diverged from other extant organisms.

**Fig. 5:**
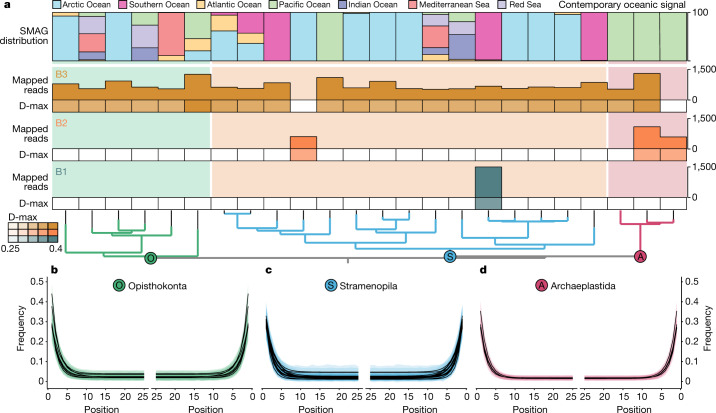
Marine planktonic eukaryotes identified at the Kap København Formation. **a**, Detection of SMAGs and average damage (D-max) of a SMAG within a member unit. Top, the SMAG distribution in contemporary oceans based on the data of Delmont et al.^63^. The SMAGs are ordered on the basis of phylogenomic inference from Delmont et al.^63^. **b**–**d**, Distribution of DNA damage among the taxonomic supergroup Opisthokonta (**b**), Stramenopila (**c**) and Archaeplastida (**d**) (Source Data 1).

The probable source of these reads is a population of *Limulus polyphemus*, the only Atlantic member of the genus, which would have spawned directly onto the sediment as it accumulated. Today this genus does not spawn north of the Bay of Fundy (about 45° N), suggesting warmer surface water conditions in the Early Pleistocene at Kap København consistent with the +8 °C annual sea surface temperature anomaly reconstructed for the Pleistocene of the coast of northeast Greenland^50^. By aligning our reads against the *Tara* Oceans eukaryotic metagenomic assembled genomes (SMAGs) data (Methods), we further reveal the presence of 24 marine planktonic taxa in 14 samples, covering both zooplankton and phytoplankton (Fig. 5). These detected SMAGs belong to the supergroups Opisthokonta (6), Stramenopila (15) and Archaeplastida (3). The majority of these signals are from SMAGs associated with cold regions in the modern ocean (that is, the Arctic Ocean and Southern Ocean), such as diatoms (Bacillariophyta), Chrysophyceae and the MAST-4 group (Supplementary Table 6.11.1), as we expected. However, a few are cosmopolitan, whereas others, such as Archaeplastida (green microalgae), have an oceanic signal that is today confined to more temperate waters in the Pacific Ocean (Fig. 5). Although we do not know whether modern day ecologies can be extrapolated to ancient ecosystems, the abundance of green microalgae is believed to be increasing in Arctic regions, which tends to be associated with warming surface waters.

## Discussion

The Kap København ancient eDNA record is extraordinary for several reasons; the upper limit of the 95% highest posterior density of the estimated molecular age is 2.0 Myr and independently supports a geological age of approximately 2 Myr (Fig. 2). This implies that the DNA is considerably older than any previously sequenced DNA^21^. Our DNA results detected five times as many plant genera as previous studies using shotgun sequencing of ancient sediments^29,34,51,52^, which is well within the range of the richest northern boreal metabarcoding records^53^. The accuracy of the assignments is strengthened by the observation that 76% of the taxa identified to the level of genus or family also occurred in macrofossil and/or pollen assemblages from the same units. Our results demonstrate the potential of ancient environmental metagenomics to reconstruct ancient environments, phylogenetically place and date ancient lineages from diverse taxa from around 2 Ma (Supplementary Information, section 6). Finally, the DNA identified a set of additional plant genera, which occur as macrofossils at other Arctic Late Pliocene and Early Pleistocene sites (Figs. 1 and 3 and Supplementary Information, section 5) but not as fossils at Kap København, thereby expanding the spatiotemporal distribution of these ancient floras.

Of note, the detection of both *Rangifer* (reindeer and caribou) and *Mammut* (mastodon) forces a revision of earlier palaeoenvironmental reconstructions based on the site’s relatively impoverished faunal record, entailing both higher productivity and habitat diversity for much of the deposition period. Because all the vertebrate taxa identified by DNA are herbivores, their representation may be a function of relative biomass (see discussion on taphonomy in Supplementary Information, section 6). Caribou, geese, hares and rodents can all be abundant, at least seasonally, in boreal environments. Additionally, the excrement of large herbivores (such as caribou and particularly mastodons) can be a significant component of sediments^34^. By contrast, carnivores are not represented, consistent with their smaller total biomass. This dynamic also explains the dominance of plant reads over metazoans and to some extent differences in representation of various plant genera (Supplementary Information, section 6). In the general absence of fossils, DNA may prove the most effective tool for reconstructing the biogeography of vertebrates through the Early Pleistocene. DNA from mastodon must imply a viable population of this large browsing megaherbivore, which would require a more productive boreal habitat than that inferred in earlier reconstructions based primarily on plant macrofossils^7^. Mastodon dung from a site in central Nova Scotia from around 75,000 years ago contained macrofossils from sedges, cattail, bulrush, bryophytes and even charophytes, but was dominated by spruce needles and birch samaras^54^. The Kap København units with mastodon DNA yielded macrofossils and DNA from *Betula* as well as more thermophilic arboreal taxa including *Thuja*, *Taxus*, *Cornus* and *Viburnum*, none of which range into Greenland’s hydric Arctic tundra or polar deserts today. The co-occurrence of these taxa in multiple units compels a revision of previous temperature estimates as well as the presence of permafrost.

No single modern plant community or habitat includes the range of taxa represented in many of the macrofossil and DNA samples from Kap København. The community assemblage represents a mixture of modern boreal and Arctic taxa, which has no analogue in modern vegetation^10,15^. To some degree, this is expected, as the ecological amplitudes of modern members of these genera have been modified by evolution^55^. Furthermore, the combination of the High Arctic photoperiod with warmer conditions and lower atmospheric CO_2_ concentrations^56^ made the Early Pleistocene climate of North Greenland very different from today. The mixed character of the terrestrial assemblage is also reflected in the marine record, where Arctic and more cosmopolitan SMAGs of Opistokonta and Stramenopila are found together with horseshoe crabs, corals and green microalgae (Archaeplastida), which today inhabit warmer waters at more southern latitudes.

Megaherbivores, particularly mastodons, could have had a significant impact on an interglacial taiga environment, even providing a top-down trophic control on vegetation structure and composition at this high latitude. The presence of mastodons^57,58^ coupled with the absence of anthropogenic fire, which has had a role in some Holocene boreal habitats^59^, are important differences. Another important factor is the proximity and biotic richness of the refugia from which pioneer species were able to disperse into North Greenland when conditions became favourable at the beginning of interglacials. The shorter duration of Early Pleistocene glaciations produced less extensive ice sheets allowing colonization from relatively species-rich coniferous-deciduous woodlands in northeastern Canada^12,60^. More extensive glaciation later in the Pleistocene increasingly isolated North Greenland and later re-colonizations were from increasingly distant and/or less diverse refugia.

In summary, we show the power of ancient eDNA to add substantial detail to our knowledge of this unique, ancient open boreal forest community intermixed with Arctic species, a community composition that has no modern analogues and included mastodons and reindeer, among others. Similar detailed flora and vertebrate DNA records may survive at other localities. If recovered, these would advance our understanding of the variability of climate and biotic interactions during the warmer Early Pleistocene epochs across the High Arctic.

## Methods

### Sampling

Sediment samples were obtained from the Kap København Formation in North Greenland (82° 24′ 00″ N 22° 12′ 00″ W) in the summers of 2006, 2012 and 2016 (see Supplementary Table 3.1.1). Sampled material consisted of organic-rich permafrost and dry permafrost. Prior to sampling, profiles were cleaned to expose fresh material. Samples were hereafter collected vertically from the slope of the hills either using a 10 cm diameter diamond headed drill bit or cutting out ~40 × 40 × 40 cm blocks. Sediments were kept frozen in the field and during transportation to the lab facility in Copenhagen. Disposable gloves and scalpels were used and changed between each sample to avoid cross-contamination. In a controlled laboratory environment, the cores and blocks were further sub-sampled for material taking only the inner part of sediment cores, leaving 1.5–2 cm between the inner core and the surface that provided a subsample of approximately 6–10 g. Subsequently, all samples were stored at temperatures below −22 °C.

We sampled organic-rich sediment by taking samples and biological replicates across the three stratigraphic units B1, B2 and B3, spanning 5 different sites, site: 50 (B3), 69 (B2), 74a (B1), 74b (B1) and 119 (B3). Each biological replicate from each unit at each site was further sampled in different sublayers (numbered L0–L4, Source Data 1, sheet 1).

### Absolute age dating

In 2014, Be and Al oxide targets from 8× 1 kg quartz-rich sand samples collected at modern depths ranging from 3 to 21 m below stream cut terraces were analysed by accelerator mass spectrometry and the cosmogenic isotope concentrations interpreted as maximum ages using a simple burial dating approach^1^ (^26^Al:^10^Be versus normalized ^10^Be). The ^26^Al and ^10^Be isotopes were produced by cosmic ray interactions with exposed quartz in regolith and bedrock surfaces in the mountains above Kap København prior to deposition. We assume that the ^26^Al:^10^Be was uniform and steady for long time periods in the upper few metres of these gradually eroding palaeo-surfaces. Once eroded by streams and hillslope processes, the quartz sand was deposited in sandy braided stream sediment, deltaic distributary systems, or the near-shore environment and remained effectively shielded from cosmic ray nucleons buried (many tens of metres) under sediment, intermittent ice shelf or ice sheet cover, and—at least during interglacials—the marine water column until final emergence. The simple burial dating approach assumes that the sand grains experienced only one burial event. If multiple burial events separated by periods of re-exposure occurred, then the starting ^26^Al:^10^Be before the last burial event would be less than the initial production ratio (6.75 to 7.42, see discussion below) owing to the relatively faster decay of ^26^Al during burial, and therefore the calculated burial age would be a maximum limiting age. Multiple burial events can be caused by shielding by thick glacier ice in the source area, or by sediment storage in the catchment prior to final deposition. These shielding events mean that the ^26^Al:^10^Be is lower, and therefore a calculated burial age assuming the initial production ratio would overestimate the final burial duration. We also consider that once buried, the sand grains may have been exposed to secondary cosmogenic muons (their depth would be too great for submarine nucleonic production). As sedimentation rates in these glaciated near-shore environments are relatively rapid, we show that even the muonic production would be negligible (see Supplemental Information). However, once the marine sediments emerged above sea level, in-situ production by both nucleogenic and muogenic production could alter the ^26^Al:^10^Be. The ^26^Al versus ^10^Be isochron plot reveals this complex burial history (Supplementary Information, section 3) and the concentration versus depth composite profiles for both ^26^Al and ^10^Be reveal that the shallowest samples may have been exposed during a period of time (~15,000 years ago) that is consistent with deglaciation in the area (Supplemental Information). While we interpret the individual simple burial age of all samples as a maximum limiting age of deposition of the Kap København Formation Member B, we recommend using the three most deeply shielded samples in a single depth profile to minimize the effect of post-depositional production. We then calculate a convolved probability distribution age for these three samples (KK06A, B and C). However, this calculation depends on the ^26^Al:^10^Be production ratio we use (that is, between 6.75 and 7.42) and on whether we adjust for erosion in the catchment. So, we repeat the convolved probability distribution function age for the lowest and highest production ratio and zero to maximum possible erosion rate, to obtain the minimum and maximum limiting age range at 1*σ* confidence (Supplementary Information, section 3). Taking the midpoint between the negative and positive 3*σ* confidence limits, we obtain a maximum burial age of 2.70 ± 0.46 Myr. This age is also supported by the position of those three samples on the isochron plot, which suggests the true age may not be significantly different that this maximum limiting age.

### Thermal age

The extent of thermal degradation of the Kap København DNA was compared to the DNA from the Krestovka Mammoth molar. Published kinetic parameters for DNA degradation^64^ were used to calculate the relative rate difference over a given interval of the long-term temperature record and to quantify the offset from the reference temperature of 10 °C, thus estimating the thermal age in years at 10 °C for each sample (Supplementary Information, section 4). The mean annual air temperature (MAT) for the the Kap København sediment was taken from Funder et al. (2001)^6^ and for the Krestovka Mammoth the MAT was calculated using temperature data from the Cerskij Weather Station (WMO no. 251230) 68.80° N 161.28° E, 32 m from the International Research Institute Data Library (https://iri.columbia.edu/) (Supplementary Table 4.4.1).

We did not correct for seasonal fluctuation for the thermal age calculation of the Kap København sediments or from the Krestovka Mammoth. We do provide theoretical average fragment length for four different thermal scenarios for the DNA in the Kap København sediments (Supplementary Table 4.4.2). A correction in the thermal age calculation was applied for altitude using the environmental lapse rate (6.49 °C km^−1^). We scaled the long-term temperature model of Hansen et al. (2013)^65^ to local estimates of current MATs by a scaling factor sufficient to account for the estimates of the local temperature decline at the last glacial maximum and then estimated the integrated rate using an activation energy (Ea) of 127 kJ mol^−1^ (ref. ^64^).

### Mineralogic composition

The minerals in each of the Kap København sediment samples were identified using X-ray diffraction and their proportions were quantified using Rietveld refinement. The samples were homogenized by grinding ~1 g of sediment with ethanol for 10 min in a McCrone Mill. The samples were dried at 60 °C and added corundum (CR-1, Baikowski) as the internal standard to a final concentration of 20.0 wt%. Diffractograms were collected using a Bruker D8 Advance (Θ–Θ geometry) and the LynxEye detector (opening 2.71°), with Cu *K*_α1,2_ radiation (1.54 Å; 40 kV, 40 mA) using a Ni-filter with thickness of 0.2 mm on the diffracted beam and a beam knife set at 3 mm. We scanned from 5–90° 2θ with a step size of 0.1° and a step time of 4 s while the sample was spun at 20 rpm. The opening of the divergence slit was 0.3° and of the antiscatter slit 3°. Primary and secondary Soller slits had an opening of 2.5° and the opening of the detector window was 2.71°. For the Rietveld analysis, we used the Profex interface for the BGMN software^66,67^. The instrumental parameters and peak broadening were determined by the fundamental parameters ray-tracing procedure^68^. A detailed description of identification of clay minerals can be found in the supporting information.

### Adsorption

We used pure or purified minerals for adsorption studies. The minerals used and treatments for purifying them are listed in Supplementary Table 4.2.6. The purity of minerals was checked using X-ray diffraction with the same instrumental parameters and procedures as listed in the above section i.e., mineralogical composition. Notes on the origin, purification and impurities can be found in the Supplementary Information section 4. We used artificial seawater^69^ and salmon sperm DNA (low molecular weight, lyophilized powder, Sigma Aldrich) as a model for eDNA adsorption. A known amount of mineral powder was mixed with seawater and sonicated in an ultrasonic bath for 15 min. The DNA stock was then added to the suspension to reach a final concentration between 20–800 μg ml^−1^. The suspensions were equilibrated on a rotary shaker for 4 h. The samples were then centrifuged and the DNA concentration in the supernatant determined with UV spectrometry (Biophotometer, Eppendorf), with both positive and negative controls. All measurements were done in triplicates, and we made five to eight DNA concentrations per mineral. We used Langmuir and Freundlich equations to fit the model to the experimental isotherm and to obtain adsorption capacity of a mineral at a given equilibrium concentration.

### Pollen

The pollen samples were extracted using the modified Grischuk protocol adopted in the Geological Institute of the Russian Academy of Science which utilizes sodium pyrophosphate and hydrofluoric acid^70^. Slides prepared from 6 samples were scanned at 400× magnification with a Motic BA 400 compound microscope and photographed using a Moticam 2300 camera. Pollen percentages were calculated as a proportion of the total palynomorphs including the unidentified grains. Only 4 of the 6 samples yielded terrestrial pollen counts ≥50. In these, the total palynomorphs identified ranged from 225 to 71 (mean = 170.25; median = 192.5). Identifications were made using several published keys^71,72^. The pollen diagram was initially compiled using Tilia version 1.5.12^73^ but replotted for this study using Psimpoll 4.10^74^.

### DNA recovery

For recovery calculation, we saturated mineral surfaces with DNA. For this, we used the same protocol as for the determination of adsorption isotherms with an added step to remove DNA not adsorbed but only trapped in the interstitial pores of wet paste. This step was important because interstitial DNA would increase the amount of apparently adsorbed DNA and overestimate the recovery. To remove trapped DNA after adsorption, we redispersed the minerals in seawater. The process of redispersing the wet paste in seawater, ultracentrifugation and removal of supernatant lasted less than 2.5 min. After the second centrifugation, the wet pastes were kept frozen until extraction. We used the same extraction protocol as for the Kap København sediments. After the extraction, the DNA concentration was again determined using UV spectrometry.

### Metagenomes

A total of 41 samples were extracted for DNA^75^ and converted to 65 dual-indexed Illumina sequencing libraries (including 13 negative extraction- and library controls)^30^. 34 libraries were thereafter subjected to ddPCR using a QX200 AutoDG Droplet Digital PCR System (Bio-Rad) following manufacturer’s protocol. Assays for ddPCR include a P7 index primer (5′-AGCAGAAGACGGCATAC-3′) (900nM), gene-targeting primer (900 nM), and a gene-targeting probe (250nM). We screened for Viridiplantae psbD (primer: 5′-TCATAATTGGACGTTGAACC-3′, probe: 5′-(FAM)ACTCCCATCATATGAAA(BHQ1)-3′) and Poaceae psbA (primer: 5′-CTCACAACTTCCCTCTAGAC-3′, probe 5′-(HEX)AGCTGCTGTTGAAGTTC(BHQ1)-3′). Additionally, 34 of the 65 libraries were enriched using targeted capture enrichment, for mammalian mitochondrial DNA using the PaleoChip Arctic1.0 bait-set^31^ and all libraries were hereafter sequenced on an Illumina HiSeq 4000 80 bp PE or a NovaSeq 6000 100 bp PE. We sequenced a total of 16,882,114,068 reads which, after low complexity filtering (Dust = 1), quality trimming (*q* ≥ 25), duplicate removal and filtering for reads longer than 29 bp (only paired read mates for NovaSeq data) resulted in 2,873,998,429 reads that were parsed for further downstream analysis. We next estimated *k*mer similarity between all samples using simka^32^ (setting heuristic count for max number of reads (-max-reads 0) and a *k*mer size of 31 (-kmer-size 31)), and performed a principal component analysis (PCA) on the obtained distance matrix (see Supplementary Information, ‘DNA’). We hereafter parsed all QC reads through HOLI^33^ for taxonomic assignment. To increase resolution and sensitivity of our taxonomic assignment, we supplemented the RefSeq (92 excluding bacteria) and the nucleotide database (NCBI) with a recently published Arctic-boreal plant database (PhyloNorway) and Arctic animal database^34^ as well as searched the NCBI SRA for 139 genomes of boreal animal taxa (March 2020) of which 16 partial-full genomes were found and added (Source Data 1, sheet 4) and used the GTDB microbial database version 95 as decoy. All alignments were hereafter merged using samtools and sorted using gz-sort (v. 1). Cytosine deamination frequencies were then estimated using the newly developed metaDMG, by first finding the lowest common ancestor across all possible alignments for each read and then calculating damage patterns for each taxonomic level^36^ (Supplementary Information, section 6). In parallel, we computed the mean read length as well as number of reads per taxonomic node (Supplementary Information, section 6). Our analysis of the DNA damage across all taxonomic levels pointed to a minimum filter for all samples at all taxonomic levels with a D-max ≥ 25% and a likelihood ratio (λ-LR) ≥ 1.5. This ensured that only taxa showing ancient DNA characteristics were parsed for downstream profiling and analysis and resulted in no taxa within any controls being found (Supplementary Information, section 6).

### Marine eukaryotic metagenome

We sought to identify marine eukaryotes by first taxonomically labelling all quality-controlled reads as Eukaryota, Archaea, Bacteria or Virus using Kraken 2^76^ with the parameters ‘--confidence 0.5 --minimum-hit-groups 3’ combined with an extra filtering step that only kept those reads with root-to-leaf score >0.25. For the initial Kraken 2 search, we used a coarse database created by the taxdb-integration workflow (https://github.com/aMG-tk/taxdb-integration) covering all domains of life and including a genomic database of marine planktonic eukaryotes^63^ that contain 683 metagenome-assembled genomes (MAGs) and 30 single-cell genomes (SAGs) from *Tara* Oceans^77^, following the naming convention in Delmont et al.^63^, we will refer to them as SMAGs. Reads labelled as root, unclassified, archaea, bacteria and virus were refined through a second Kraken 2 labelling step using a high-resolution database containing archaea, bacteria and virus created by the taxdb-integration workflow. We used the same Kraken 2 parameters and filtering thresholds as the initial search. Both Kraken 2 databases were built with parameters optimized for the study read length (--kmer-len 25 --minimizer-len 23 --minimizer-spaces 4).

Reads labelled as eukaryota, root and unclassified were hereafter mapped with Bowtie2^78^ against the SMAGs. We used MarkDuplicates from Picard (https://github.com/broadinstitute/picard) to remove duplicates and then we calculated the mapping statistics for each SMAG in the BAM files with the filterBAM program (https://github.com/aMG-tk/bam-filter). We furthermore estimated the postmortem damage of the filtered BAM files with the Bayesian methods in metaDMG and selected those SMAGs with a D-max ≥ 0.25 and a fit quality (λ**-**LR) higher than 1.5. The SMAGs with fewer than 500 reads mapped, a mean read average nucleotide identity (ANI) of less than than 93% and a breadth of coverage ratio and coverage evenness of less than 0.75 were removed. We followed a data-driven approach to select the mean read ANI threshold, where we explored the variation of mapped reads as a function of the mean read ANI values from 90% to 100% and identified the elbow point in the curve (Supplementary Fig. 6.11.1). We used anvi’o^79^ in manual mode to plot the mapping and damage results using the SMAGs phylogenomic tree inferred by Delmont et al. as reference. We used the oceanic signal of Delmont et al. as a proxy to the contemporary distribution of the SMAGs in each ocean and sea (Fig. 5 and Supplementary Information, section 6).

### Comparison of DNA, macrofossil and pollen

To allow comparison between records in DNA, macrofossil and pollen, the taxonomy was harmonized following the Pan Arctic Flora checklist^43^ and NCBI. For example, since Bennike (1990)^18^, *Potamogeton* has been split into *Potamogeton* and *Stuckenia*, *Polygonym* has been split to *Polygonum* and *Bistorta*, and *Saxifraga* was split to *Saxifraga* and *Micranthes*, whereas others have been merged, such as *Melandrium* with *Silene*^40^. Plant families have changed names—for instance, Gramineae is now called Poaceae and Scrophulariaceae has been re-circumscribed to exclude Plantaginaceae and Orobancheae^80^. We then classified the taxa into the following: category 1 all identical genus recorded by DNA and macrofossils or pollen, category 2 genera recorded by DNA also found by macrofossils or pollen including genus contained within family level classifications, category 3 taxa only recorded by DNA, category 4 taxa only recorded by macrofossils or pollen (Source Data 1).

### Phylogenetic placement

We sought to phylogenetically place the set of ancient taxa with the most abundant number of reads assigned, and with a sufficient number of reference sequences to build a phylogeny. These taxa include reads mapped to the chloroplast genomes of the flora genera *Salix*, *Populus* and *Betula*, and to the mitochondrial genomes of the fauna families Elephantidae, Cricetidae, Leporidae, as well as the subfamilies Capreolinae and Anserinae. Although the evolution of the chloroplast genome is somewhat less stable than that of the plant mitochondrial genome, it has a faster rate of evolution, and is non-recombining, and hence is more likely to contain more informative sites for our analysis than the plant mitochondria^81^. Like the mitochondrial genome, the chloroplast genome also has a high copy number, so that we would expect a high number of sedimentary reads mapping to it.

For each of these taxa, we downloaded a representative set of either whole chloroplast or whole mitochondrial genome fasta sequences from NCBI Genbank^82^, including a single representative sequence from a recently diverged outgroup. For the *Betula* genus, we also included three chloroplast genomes from the PhyloNorway database^34,83^. We changed all ambiguous bases in the fasta files to N. We used MAFFT^84^ to align each of these sets of reference sequences, and inspected multiple sequence alignments in NCBI MSAViewer to confirm quality^85^. We trimmed mitochondrial alignments with insufficient quality due to highly variable control regions for Leporidae, Cricetidae and Anserinae by removing the d-loop in MegaX^86^.

The BEAST suite^49^ was used with default parameters to create ultrametric phylogenetic trees for each of the five sets of taxa from the multiple sequence alignments (MSAs) of reference sequences, which were converted from Nexus to Newick format in Figtree (https://github.com/rambaut/figtree). We then passed the multiple sequence alignments to the Python module AlignIO from BioPython^87^ to create a reference consensus fasta sequence for each set of taxa. Furthermore, we used SNPSites^88^ to create a vcf file from each of the MSAs. Since SNPSites outputs a slightly different format for missing data than needed for downstream analysis, we used a custom R script to modify the vcf format appropriately. We also filtered out non-biallelic SNPs.

From the damage filtered ngsLCA output, we extracted all readIDs uniquely classified to reference sequences within these respective taxa or assigned to any common ancestor inside the taxonomic group and converted these back to fastq files using seqtk (https://github.com/lh3/seqtk). We merged reads from all sites and layers to create a single read set for each respective taxon. Next, since these extracted reads were mapped against a reference database including multiple sequences from each taxon, the output files were not on the same coordinate system. To circumvent this issue and avoid mapping bias, we re-mapped each read set to the consensus sequence generated above for that taxon using bwa^89^ with ancient DNA parameters (bwa aln -n 0.001). We converted these reads to bam files, removed unmapped reads, and filtered for mapping quality > 25 using samtools^90^. This produced 103,042, 39,306, 91,272, 182 and 129 reads for *Salix*, *Populus*, *Betula*, Elephantidae and Capreolinae, respectively.

We next used pathPhynder^62^, a phylogenetic placement algorithm that identifies informative markers on a phylogeny from a reference panel, evaluates SNPs in the ancient sample overlapping these markers, and traverses the tree to place the ancient sample according to its derived and ancestral SNPs on each branch. We used the transversions-only filter to avoid errors due to deamination, except for *Betula*, *Salix* and *Populus* in which we used no filter due to sufficiently high coverage. Last, we investigated the pathPhynder output in each taxon set to determine the phylogenetic placement of our ancient samples (see Supplementary Information for discussion on phylogenetic placement).

Based on the analysis described above we further investigated the phylogenetic placement within the genus *Mammut*, or mastodons. To avoid mapping reference biases in the downstream results, we first built a consensus sequence from all comparative mitochondrial genomes used in said analysis and mapped the reads identified in ngsLCA as Elephantidae to the consensus sequence. Consensus sequences were constructed by first aligning all sequences of interest using MAFFT^84^ and taking a majority rule consensus base in Geneious v2020.0.5 (https://www.geneious.com). We performed three analyses for phylogenetic placement of our sequence: (1) Comparison against a single representative from each Elephantidae species including the sea cow (*Dugong dugon*) as outgroup, (2) Comparison against a single representative from each Elephantidae species, and (3) Comparison against all published mastodon mitochondrial genomes including the Asian elephant *as* outgroup.

For each of these analyses we first built a new reference tree using BEAST v1.10.4 (ref. ^47^) and repeated the previously described pathPhynder steps, with the exception that the pathPhynder tree path analysis for the *Mammut* SNPs was based on transitions and transversions, not restricting to only transversions due to low coverage.

#### *Mammut americanum*

We confirmed the phylogenetic placement of our sequence using a selection of Elephantidae mitochondrial reference sequences, GTR+G, strict clock, a birth-death substitution model, and ran the MCMC chain for 20,000,000 runs, sampling every 20,000 steps. Convergence was assessed using Tracer^91^ v1.7.2 and an effective sample size (ESS) > 200. To determine the approximate age of our recovered mastodon mitogenome we performed a molecular dating analysis with BEAST^47^ v1.10.4. We used two separate approaches when dating our mastodon mitogenome, as demonstrated in a recent publication^92^. First, we determined the age of our sequence by comparing against a dataset of radiocarbon-dated specimens (*n* = 13) only. Secondly, we estimated the age of our sequence including both molecularly (*n* = 22) and radiocarbon-dated (*n* = 13) specimens using the molecular dates previously determined^92^. We utilized the same BEAST parameters as Karpinski et al.^92^ and set the age of our sample with a gamma distribution (5% quantile: 8.72 × 10^4^, Median: 1.178 × 10^6^; 95% quantile: 5.093 × 10^6^; initial value: 74,900; shape: 1; scale: 1,700,000). In short, we specified a substitution model of GTR+G4, a strict clock, constant population size, and ran the Markov Chain Monte Carlo chain for 50,000,000 runs, sampling every 50,000 steps. Convergence of the run was again determined using Tracer.

### Molecular dating methods

In this section, we describe molecular dating of the ancient birch (*Betula*) chloroplast genome using BEAST v1.10.4 (ref. ^47^). In principle, the genera *Betula*, *Populus* and *Salix* had both sufficiently high chloroplast genome coverage (with mean depth 24.16×, 57.06× and 27.04×, respectively, although this coverage is highly uneven across the chloroplast genome) and enough reference sequences to attempt molecular dating on these samples. Notably, this is one of the reasons we included a recently diverged outgroup with a divergence time estimate in each of these phylogenetic trees. However, our *Populus* sample clearly contained a mixture of different species, as seen from its inconsistent placement in the pathPhynder output. In particular, there were multiple supporting SNPs to both *Populus balsamifera* and *Populus trichocarpa*, and both supporting and conflicting SNPs on branches above. Furthermore, upon inspection, our *Salix* sample contained a surprisingly high number of private SNPs which is inconsistent with any ancient or even modern age, especially considering the number of SNPs assigned to the edges of the phylogenetic tree leading to other *Salix* sequences. We are unsure what causes this inconsistency but hypothesize that our *Salix* sample is also a mixed sample, containing multiple *Salix* species that diverged from the same placement branch on the phylogenetic tree at different time periods. This is supported by looking at all the reads that cover these private SNP sites, which generally appear to be from a mixed sample, with reads containing both alternate and reference alleles present at a high proportion in many cases. Alternatively, or potentially jointly in parallel, this could be a consequence of the high number of nuclear plastid DNA sequences (NUPTs) in *Salix*^93^. Because of this, we continued with only *Betula*.

First, we downloaded 27 complete reference *Betula* chloroplast genome sequences and a single Alnus chloroplast genome sequence to use as an outgroup from the NCBI Genbank repository, and supplemented this with three *Betula* chloroplast sequences from the PhyloNorway database generated in a recent study^29^, for a total of 31 reference sequences. Since chloroplast sequences are circular, downloaded sequences may not always be in the same orientation or at the same starting point as is necessary for alignment, so we used custom code (https://github.com/miwipe/KapCopenhagen) that uses an anchor string to rotate the reference sequences to the same orientation and start them all from the same point. We created a MSA of these transformed reference sequences with Mafft^84^ and checked the quality of our alignment by eye in Seqotron^94^ and NCBI MsaViewer. Next, we called a consensus sequence from this MSA using the BioAlign consensus function^87^ in Python, which is a majority rule consensus caller. We will use this consensus sequence to map the ancient *Betula* reads to, both to avoid reference bias and to get the ancient *Betula* sample on the same coordinates as the reference MSA.

From the last common ancestor output in metaDMG^36^, we extracted read sets for all units, sites and levels that were uniquely classified to the taxonomic level of *Betula* or lower, with at a minimum sequence similarity of 90% or higher to any *Betula* sequence, using Seqtk^95^. We mapped these read sets against the consensus *Betula* chloroplast genome using BWA^89^ with ancient DNA parameters (-o 2 -n 0.001 -t 20), then removed unmapped reads, quality filtered for read quality ≥25, and sorted the resulting bam files using samtools^89^. For the purpose of molecular dating, it is appropriate to consider these read sets as a single sample, and so we merged the resulting bam files into one sample using samtools. We used bcftools^89^ to make an mpileup and call a vcf file, using options for haploidy and disabling the default calling algorithm, which can slightly biases the calls towards the reference sequence, in favour of a majority call on bases that passed the default base quality cut-off of 13. We included the default option using base alignment qualities^96^, which we found greatly reduced the read depths of some bases and removed spurious SNPs around indel regions. Lastly, we filtered the vcf file to include only single nucleotide variants, because we do not believe other variants such as insertions or deletions in an ancient environmental sample of this type to be of sufficiently high confidence to include in molecular dating.

We downloaded the gff3 annotation file for the longest *Betula* reference sequence, MG386368.1, from NCBI. Using custom R code^97^, we parsed this file and the associated fasta to label individual sites as protein-coding regions (in which we labelled the base with its position in the codon according to the phase and strand noted in the gff3 file), RNA, or neither coding nor RNA. We extracted the coding regions and checked in Seqotron^94^ and R that they translated to a protein alignment well (for example, no premature stop codons), both in the reference sequence and the associated positions in the ancient sequence. Though the modern reference sequence’s coding regions translated to a high-quality protein alignment, translating the associated positions in the ancient sequence with no depth cut-off leads to premature stop codons and an overall poor quality protein alignment. On the other hand, when using a depth cut-off of 20 and replacing sites in the ancient sequence which did not meet this filter with N, we see a high-quality protein alignment (except for the N sites). We also interrogated any positions in the ancient sequence which differed from the consensus, and found that any suspicious regions (for example, with multiple SNPs clustered closely together spatially in the genome) were removed with a depth cut-off of 20. Because of this, we moved forward only with sites in both the ancient and modern samples which met a depth cut-off of at least 20 in the ancient sample, which consisted of about 30% of the total sites.

**Extended Data Fig. 9:**
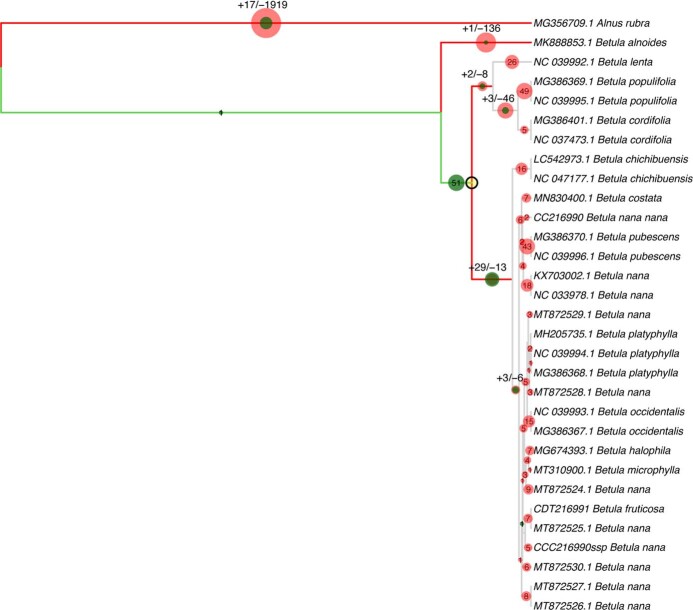
Phylogenetic placement results for our *Betula* chloroplast reads, using both transition and transversion SNPs, and using reads merged from all layers and sites. Our ancient *Betula* sample was placed basal to a main *Betula* clade, based on 29 supporting (green) and 13 conflicting (red) SNPs on its placement branch, and with very low numbers of supporting SNPs elsewhere in the tree other than those leading to this branch. This placement agrees with the BEAST molecular dating analysis (see Molecular Dating Methods).

**Extended Data Fig 10:**
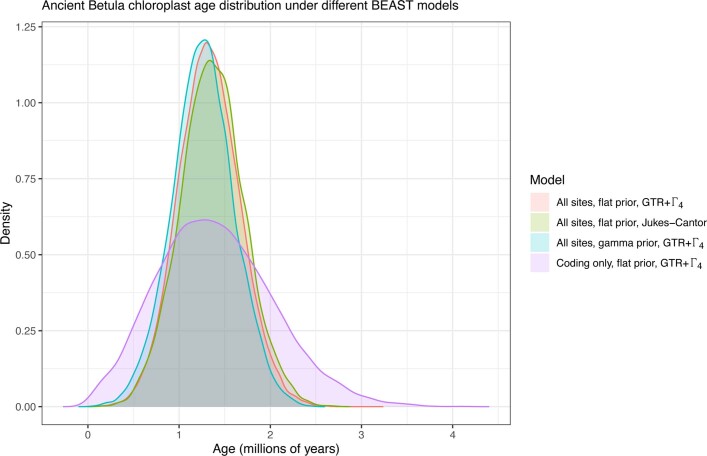
Molecular age distribution. Results of running the ancient *Betula* chloroplast molecular dating analysis BEAST v1.10.4 (ref. ^47^) with different priors and nucleotide substitution models. Using only coding regions, and therefore fewer total sites, gives a larger confidence interval as expected. Results reported in the text are for the red curve, with a flat prior and a GTR+Γ_4_ substitution model.

Next, we parsed this annotation through the multiple sequence alignment to create partitions for BEAST^47^. After checking how many polymorphic and total sites were in each, we decided to use four partitions: (1) sites belonging to protein-coding positions 1 and 2, (2) coding position 3, (3) RNA, or (4) non-coding and non-RNA. To ensure that these were high confidence sites, each partition also only included those positions which had at least depth 20 in the ancient sequence and had less than 3 total gaps in the multiple sequence alignment. This gave partitions which had 11,668, 5,828, 2,690 and 29,538 sites, respectively. We used these four partitions to run BEAST^47^ v1.10.4, with unlinked substitution models for each partition and a strict clock, with a different relative rate for each partition. (There was insufficient information in these data to infer between-lineage rate variation from a single calibration). We assigned an age of 0 to all of the reference sequences, and used a normal distribution prior with mean 61.1 Myr and standard deviation 1.633 Myr for the root height^48^; standard deviation was obtained by conservatively converting the 95% HPD to *z*-scores. For the overall tree prior, we selected the coalescent model. The age of the ancient sequence was estimated following the overall procedures of Shapiro et al. (2011)^98^. To assess sensitivity to prior choice for this unknown date, we used two different priors, namely a gamma distribution metric towards a younger age (shape = 1, scale = 1.7); and a uniform prior on the range (0, 10 Myr). We also compared two different models of rate variation among sites and substitution types within each partition, namely a GTR+G with four rate categories, and base frequencies estimated from the data, and the much simpler Jukes Cantor model, which assumed no variation between substitution types nor sites within each partition. All other priors were set at their defaults. Neither rate model nor prior choice had a qualitative effect on results (Extended Data Fig. 10). We also ran the coding regions alone, since they translated correctly and are therefore highly reliable sites and found that they gave the same median and a much larger confidence interval, as expected when using fewer sites (Extended Data Fig. 10). We ran each Markov chain Monte Carlo for a total of 100 million iterations. After removing a burn-in of the first 10%, we verified convergence in Tracer^91^ v1.7.2 (apparent stationarity of traces, and all parameters having an Effective Sample Size > 100). We also verified that the resulting MCC tree from TreeAnnotator^47^ had placed the ancient sequence phylogenetically identically to pathPhynder^62^ placement, which is shown in Extended Data Fig. 9. For our major results, we report the uniform ancient age prior, and the GTR+G_4_ model applied to each of the four partitions. The associated XML is given in Source Data 3. The 95% HPD was (2.0172,0.6786) for the age of the ancient *Betula* chloroplast sequence, with a median estimate of 1.323 Myr, as shown in Fig. 2.

### Reporting summary

Further information on research design is available in the Nature Portfolio Reporting Summary linked to this article.

## Online content

Any methods, additional references, Nature Portfolio reporting summaries, source data, extended data, supplementary information, acknowledgements, peer review information; details of author contributions and competing interests; and statements of data and code availability are available at 10.1038/s41586-022-05453-y.

### Supplementary information

Supplementary Information This file contains background as well as additional information and discussion on the methods and intermediate results related to the main findings of Palaeomagnetism, previous age control, cosmogenic dating, minerology, micro- and macrofossil analysis, and eDNA presented in the main text. It also lists the PhyloNorway consortium members.

Reporting Summary

Supplementary Data 1 This file contains tables with the DNA sequence metadata and sample-geological unit information, the comparisons between DNA, macrofossils, phylogenetic placements, additional references added for the genomic databases, the digital droplet PCR results, and the tables used for plotting the taxonomic profiles (both raw counts and the percentages).

Supplementary Data 2 Supplementary Data 2 contains all X-ray diffraction results, mineral experiments treatments and adsorption, as well as the thermal age calculation.

Supplementary Data 3 Supplementary Data 3 is the .xml file with the *Betula* molecular age estimate.

Supplementary Data 4 The individual X-ray diffraction profiles obtained.

Supplementary Data 5 Supplementary Data 5 are the calculated intensity and their differences as .tiff files.

## Data Availability

Raw sequence data (13,135,646,556 reads following adapter trimming) are available through the ENA project accession PRJEB55522. Pollen counts are available through https://github.com/miwipe/KapCopenhagen.git. Source data are provided with this paper.

## Code availability

All code used is available at https://github.com/miwipe/KapCopenhagen.git.
