# Supplementary material for: A 2-million-year-old ecosystem in Greenland uncovered by environmental DNA
Source: Nature. 2022 Dec 7;612(7939):283–91. doi: 10.1038/s41586-022-05453-y (PMC9729109; doi:10.1038/s41586-022-05453-y)
Supplement: Supplementary file 1 — This file contains background as well as additional information and discussion on the methods and intermediate results related to the main findings of Palaeomagnetism, previous age control, cosmogenic dating, minerology, micro- and macrofossil analysis, and eDNA presented in the main text. It also lists the PhyloNorway consortium members. [file 41586_2022_5453_MOESM1_ESM.pdf]

nature portfolio

https://doi.org/10.1038/s41586-022-05453-y

Supplementary information

A 2-million-year-old ecosystem in Greenland uncovered by environmental DNA

In the format provided by the authors and unedited

Supplementary information for "A 2-Million-year-old ecosystem in Greenland uncovered by Environmental DNA" Kjær et al.

Contents:

1. Paleomagnetic dating of Kap København Formation 2
   1.2 Method background and description 3
   1.3 Experimental setup 3
   1.4 Paleomagnetic results 4
   1.5 Interpretation and discussion 8
2. Previous age control of Kap København Formation 9
3. Cosmogenic nuclide burial dating 13
   3.1 Field constraints on post-depositional exposure 15
   3.2 TCN analytical methods 18
   3.3 Simple burial age calculation 23
   3.4 TCN concentration depth profiles 31
   3.5 ^{26}Al vs ^{10}Be isochron 39
   3.6 Favoured interpretation of the burial age of the Kap København Formation 41
4. Mineralogy 43
   4.1 Clay mineral characterization 43
   4.2 Mineralogic composition 44
   4.3 Adsorption 50
      4.3.1 Adsorption isotherms 50
   4.4 Thermal age 51
      4.4.1 Chain scission 52
5. Kap København plant micro and macrofossils 53
   5.1 Microfossils (pollen and spores) 53
   5.2 Macrofossils 54
6. DNA 54
   6.1 DNA sample metadata 54
   6.2 Database assemblage and taxonomic classification 54
   6.3 Sequence similarity comparison between samples in kmer space 55
   6.4 Ancient DNA authenticity 56

6.4.1 Library construct strategy 56
6.4.2 DNA damage and fragmentation 57
6.5 Capture enrichment 74
6.6 Comparing plant taxonomic profiles 78
6.7 Sensitivity and coverage analysis for the phylogenetically placed taxa. 86
6.7.1 General discussion on the phylogenetic placements 91
6.8 Phylogenetic placement of the *Mammut* 91
6.9 Plant taxa discussion Plio-Pleistocene DNA, fossil comparison 97
6.10 DNA taphonomy 97
6.11 Marine eukaryotic profiling. 99
6.12. Droplet digital PCR 102
7. PhyloNorway consortium 102
8. References 103

1. Paleomagnetic dating of Kap København Formation

Magnetostratigraphy can be used as a relative dating method during the Quaternary Period. Reversed natural remanent magnetizations (NRM) can be confidently assigned ages that are older than the boundary between the current normal polarity Bruhnes chron (C1n 0.733-0 Ma) and the preceding reversed polarity Matuyama chron (C1r 2.595-0.733 Ma)[104]. Relatively short normal polarity events during the Matuyama chron, namely the Feni (2.140-2.116 Ma), Olduvai (1.934-1.775 Ma), (Cobb Mountain (1.215-1.180 Ma), and Jaramillo (1.070-0.990) can also give rise to normal natural remanent magnetizations during the Quaternary Period[104].

A previous paleomagnetic study of Kap København sediment unit B2 relied on AF demagnetization at 10 and 20 milliTesla (mT) to determine the polarity[17], and a study of the formation at Store Koldeway applied the same procedure[105]. The data obtained from these earlier paleomagnetic studies were not sufficient to isolate characteristic remanent magnetizations (ChRMs) using principal component analysis (PCA) and paleomagnetic directions altered significantly between the demagnetization steps. Note that Abrahamsen and Marcussen (1986)[17] reconstructed scattered declinations of paleomagnetic samples taken from unit B2, which was assigned to the high

geomagnetic latitude of the site. At high latitudes, where the objective is to use sediment-based paleomagnetic data for magnetostratigraphic purposes, the angle of paleomagnetic inclination is the best available means of determining the polarity of the Earth's geomagnetic field close to the time of sediment deposition. Post-depositional formation of magnetic minerals in sediments, caused by chemical changes, can give rise to secondary magnetizations that must be identified[106]. Aborted attempts by the Earth's magnetic field to reverse polarity are termed 'geomagnetic excursions' and take place on timescales of a few millennia. Such short duration excursions are unlikely to be recorded in the Kap København Formation due to the relatively low sediment accumulation rate.

1.2 Method background and description

A total of sixty-nine samples were collected for determination of the polarity of NRM of Member A (48 samples) and fine-grained layers in unit B2 (21 samples). Standard plastic paleomagnetic sampling boxes, approximately cubic with an internal volume of 7 cm^3, were used to sample the sediment in-situ during fieldwork and the orientation of each sample was measured with a Silva magnetic compass.

1.3 Experimental setup

Measurements of the NRMs and progressive alternating field (AF) demagnetization of the samples were undertaken using a 2G-Enterprises 760-SRM equipped with in-line AF coils and magnetic shield extensions at the Palaeomagnetic and Mineral Magnetic Laboratory at the University of Lund, Sweden. After measurement of the NRM the 69 samples were progressively demagnetized in three axes in the following sequence: 5, 10, 15, 20, 30, 40 50, 60 and 100 mT AF. The ChRM of each sample was determined using the principal component analysis routine provided by the *PuffinPlot* paleomagnetic software[107]. The efficiency of demagnetization varied between samples, and it was necessary to use a different range of AF steps to identify the ChRM with a minimum of three successive points and obtain a maximum angular deviation (MAD) below the generally accepted limit of 5°.

1.4 Paleomagnetic results

The results of the paleomagnetic analysis are shown in Table S1.4.1. The intensity of the NRM varies between 1.95E-2 A/m and 8.11E-1 A/m, which is relatively strong for natural sediments. Most samples contained a weak viscous component that was removed by AF < 10 mT. A principal component of magnetization was generally identified between alternating fields of 10 and 50 mT, and in some cases 5 and 60 mT. It was possible to identify ChRMs with a MAD < 5° in 64 samples. Samples taken from Member A contain a principal component that has a reversed magnetization. Samples taken from unit B2 contain a principal component that has a normal magnetization.

Table S1.4.1: Summary of Paleomagnetic data from Kap København Formation

Sample	Locality	Me-mber	NRM Intensity (A/m)	ChRM inclination (°)	MAD (°)	AF steps used for PCA (mT)	Polarity of ChRM
A71-0	120	A	1.95E-02	-20.94	5.25	20-50	Not defined
A71-2	120	A	1.34E-01	-51.62	3.02	10-50	REVERSED
A71-5	120	A	2.61E-01	-76.32	1.48	10-50	REVERSED
A72-4	120	A	4.60E-01	-71.41	1.02	10-50	REVERSED
A72-8	120	A	1.28E-01	-65.36	2.33	10-50	REVERSED
A72-9	120	A	3.09E-01	-69.15	1.95	10-50	REVERSED
B73-1	120	A	2.38E-01	-52.9	2.15	10-50	REVERSED
B73-2	120	A	3.54E-01	-80.82	3.5	10-50	REVERSED
B73-3	120	A	7.88E-02	-37.71	5.01	10-50	REVERSED

B73-4	120	A	1.28E-01	-38.54	3	10-40	REVERSED
B74-2	120	A	1.12E-01	8.36	3.45	10-40	Not defined
B75-3	120	A	3.08E-01	N/A	N/A	N/A	Not defined
B75-6	120	A	2.50E-01	-41.53	1.34	10-50	Not defined
B76-2	120	A	1.12E-01	-80.11	1.3	20-50	REVERSED
B76-3	120	A	2.96E-01	-59.5	1.61	15-50	REVERSED
B76-6	120	A	1.67E-01	-56.87	2.59	10-50	REVERSED
C71-7	120	A	3.07E-01	-9.36	2.8	10-50	Not defined
C72-0	120	A	2.67E-01	-52.43	2.48	10-50	REVERSED
C72-2	120	A	4.52E-01	-59.4	0.73	10-50	REVERSED
C72-3	120	A	3.95E-01	-76.59	0.59	10-50	REVERSED
C72-4	120	A	4.46E-01	-73.72	2.87	10-50	REVERSED
C72-7	120	A	1.86E-01	-38.26	1.04	10-50	REVERSED
C73-0	120	A	5.57E-01	-75.72	1.18	15-50	REVERSED
C73-1	120	A	5.26E-01	-77.1	0.81	10-50	REVERSED
D79-8	120	A	3.48E-01	-65.63	0.63	10-50	REVERSED
D79-9	120	A	2.78E-01	-78.45	1.29	10-50	REVERSED
D80-0	120	A	2.64E-01	-76.57	2.9	0-20	REVERSED
D80-1	120	A	1.36E-01	-69.83	4.04	0-20	REVERSED
D80-3	120	A	4.02E-01	-84.22	0.43	10-40	REVERSED
D80-4	120	A	5.87E-01	-70.21	0.49	10-50	REVERSED

Sample	Locality	Member	NRM Intensity (A/m)	ChRM inclination (°)	MAD (°)	AF steps used for PCA (mT)	Polarity of ChRM
D80-5	120	A	3.36E-01	-78.07	1.11	10-50	REVERSED
D80-6	120	A	2.86E-01	-78.28	0.5	10-50	REVERSED
D80-7	120	A	8.11E-01	-80.74	1.85	10-50	REVERSED
KK1-97-1	69	B2	5.81E-02	76.13	4.97	5-60	NORMAL
KK1-97-2	69	B2	6.79E-02	63	2.46	5-60	NORMAL
KK1-97-3	69	B2	2.55E-01	50.75	6.4	5-60	NORMAL
KK1-97-4	69	B2	1.94E-01	47.67	5.39	5-30	NORMAL
KK1-97-5	69	B2	1.36E-01	57.45	2.05	5-30	NORMAL
KK1-97-6	69	B2	1.88E-01	49.96	3.51	5-30	NORMAL
KK1-97-7	69	B2	1.28E-01	71.93	4.1	5-50	NORMAL
KK1-97-8	69	B2	1.29E-01	67.49	3.37	5-30	NORMAL
KK1-97-9	69	B2	1.43E-01	76.61	4.12	5-60	NORMAL
KK1-98-0	69	B2	1.90E-01	71.89	3.24	5-40	NORMAL
KK1-98-1	69	B2	1.74E-01	83.1	0.95	5-60	NORMAL
KK1-98-2	69	B2	1.85E-01	81.3	0.82	5-60	NORMAL
KK1-98-3	69	B2	2.35E-01	65.99	2.55	5-40	NORMAL
KK1-98-4	69	B2	2.46E-01	68.39	2.52	5-40	NORMAL

KK1-98-5	69	B2	3.13E-01	66.13	3.09	5-40	NORMAL
KK1-104-55	69	B2	1.69E-01	43.39	3.03	5-40	Not defined
KK1-98-6	69	B2	1.82E-01	85.49	0.4	5-60	NORMAL
KK1-104-6	69	B2	1.27E-01	62.52	3.62	5-40	NORMAL
KK1-104-8	69	B2	1.17E-01	55.25	4.22	5-40	NORMAL
KK1-104-9	69	B2	1.77E-01	68.18	1.75	5-40	NORMAL
KK1-105-0	69	B2	6.96E-02	83.2	1.07	5-50	NORMAL
KK4-53-0	173	A	1.56E-01	-82.88	1.31	15-60	REVERSED
KK4-53-1	173	A	2.76E-01	-80.84	0.96	15-60	REVERSED
KK4-53-3	173	A	6.90E-02	-72.17	8.77	20-50	Not defined
KK4-53-4	173	A	2.80E-01	-78.66	0.69	15-60	REVERSED
KK4-53-5	173	A	2.62E-02	-76.24	1.87	15-60	REVERSED
KK4-53-6	173	A	4.68E-01	-69.27	0.86	15-60	REVERSED
KK4-53-8	173	A	2.31E-01	-41.93	2.67	15-60	REVERSED
KK4-54-1	173	A	1.27E-01	-52.65	1.68	15-40	REVERSED
KK4-54-3	173	A	7.48E-02	63.6	9.6	5-40	NORMAL
KK4-54-5	173	A	5.07E-01	-77.43	0.72	10-50	REVERSED
KK4-54-9	173	A	3.39E-01	-87.23	2.87	5-60	REVERSED

KK4-55-2	173	A	4.53E-01	-87.11	0.56	15-60	REVERSED
KK4-55-3	173	A	2.50E-01	-81.91	1.91	15-60	REVERSED
KK4-55-7	173	A	2.43E-01	-51.98	0.75	15-50	REVERSED
KK4-56-2	173	A	5.65E-01	-80.87	0.94	15-60	REVERSED

A significant feature of the paleomagnetic data set is that the samples from unit B2 alter polarity when demagnetized at Afs > 60 mT. In most of the samples from unit B2 the intensity of the magnetization increased between 60 and 100 mT AF. Simple reorientation tests indicated that the magnetization remaining at 100 mT AF was not related to the geometry of the demagnetization coils. Thus, this stable magnetization is not the artefact of an uncontrolled anhysteretic remanent magnetization due to imperfect shielding, but a real component of the natural remanent magnetization.

1.5 Interpretation and discussion

The natural remanent magnetization data and AF demagnetization spectra reveal a stable component that is removed during demagnetization up to 60 mT, and thus most likely carried by pseudo-single-domain and multi-domain titanomagnetite ($Fe3_{-x}Ti_xO_4$). Our interpretation of the ChRMs obtained through PCA of this component implies that Member A was deposited prior to the Matuyama-Brunhes polarity reversal, i.e., these sediments were deposited before 0.733 Ma. UnitB2 has a dominant normal polarity ChRM that is carried by a mineral that has ferrimagnetic properties similar to the mineral that carries the reversed magnetization in Member A.

If the depositional sequence was formed during the Quaternary the reversed polarity magnetizations characteristic of Member A must have been acquired during the Matayama main polarity chron (comprising C1r.1r, C1r.2r, C1r.3r, C2r.1r and C2r.2r), while the normal polarity magnetizations characteristic of Unit B2 could have formed during the Jaramillo (C1r.1n), Cobb Mountain (C1r.2n), Olduvai (C2n) subchrons or possibly the Feni (C2r.1n) cryptochron. If the depositional sequence is older, Member B could have formed during the upper part of Gauss (C2An.1n) and Member A in Keana (C2An.1r). There are different palaeomagnetic scenarios with different likelihoods. The most probable scenario, given the other geochronologic information available, is that Member A was

deposited during C2An.1r (Keana) and that unit B2 was deposited during C2An.1n (Gauss). A slightly younger scenario is that Member A is C2r.2r and Member B is C2r.1n (Feni).

2. Previous age control of Kap København Formation

Since the discovery of Kap København Formation in 1978, much effort has been dedicated to study the rich macro- and microfossil content and in acquiring an age for this sequence[8]. The 10-90 m thick sequence has been investigated at more than 170 sites and it has formally been divided into two members. The lower Member A consists of laminated mud, and it is overlain by 40-50 m of horizontally laminated and mega-scale cross bedded sand[18]. Most studies interpret the sequence as being deposited in a shallow marine environment without any breaks in the sedimentation. In the most recent review about the Kap København Formation, the sequence was furthermore interpreted to represent a glacial-interglacial cycle as evidenced by the sea-level history, but also matched by the terrestrial flora and insects fauna as well as the marine foraminifera and molluscan fauna[7].
Conceptually, the sea-level history derived from the Kap København sequence reflects a single isostatic-eustatic cycle following a major deglaciation[7] (Fig. 1). Succeeding an ice sheet advance which left a glacial till, 50 m of laminated mud (Member A) accumulated offshore during a forced regression (Fig. 1). Marine microfossils and molluscs indicate deposition during Arctic glaciomarine conditions with high sedimentation rates[14]. Following this low-stand phase, 40-50 m of horizontally laminated and mega-scale cross bedded sand (Member B) was deposited during a transgression phase. The transition from unit A into B is characterised by a coarsening upward sequence most clearly seen at type locality 50 (Fig.1). Three units, B1, B2 and B3 represent a transition towards a high-stand maximum associated with a climatic change from low Arctic to boreal conditions. During most of the deposition of Member B, sedimentation was sufficient to adjust accommodation space to the sea level rise, except for unit B2 where sea level rise overtook the sediment supply and the water depth increased in the basin. Thus, unit B2 tends to be finer grained than units B1 and B3, which are dominated, respectively by lower and upper shoreface facies[7].

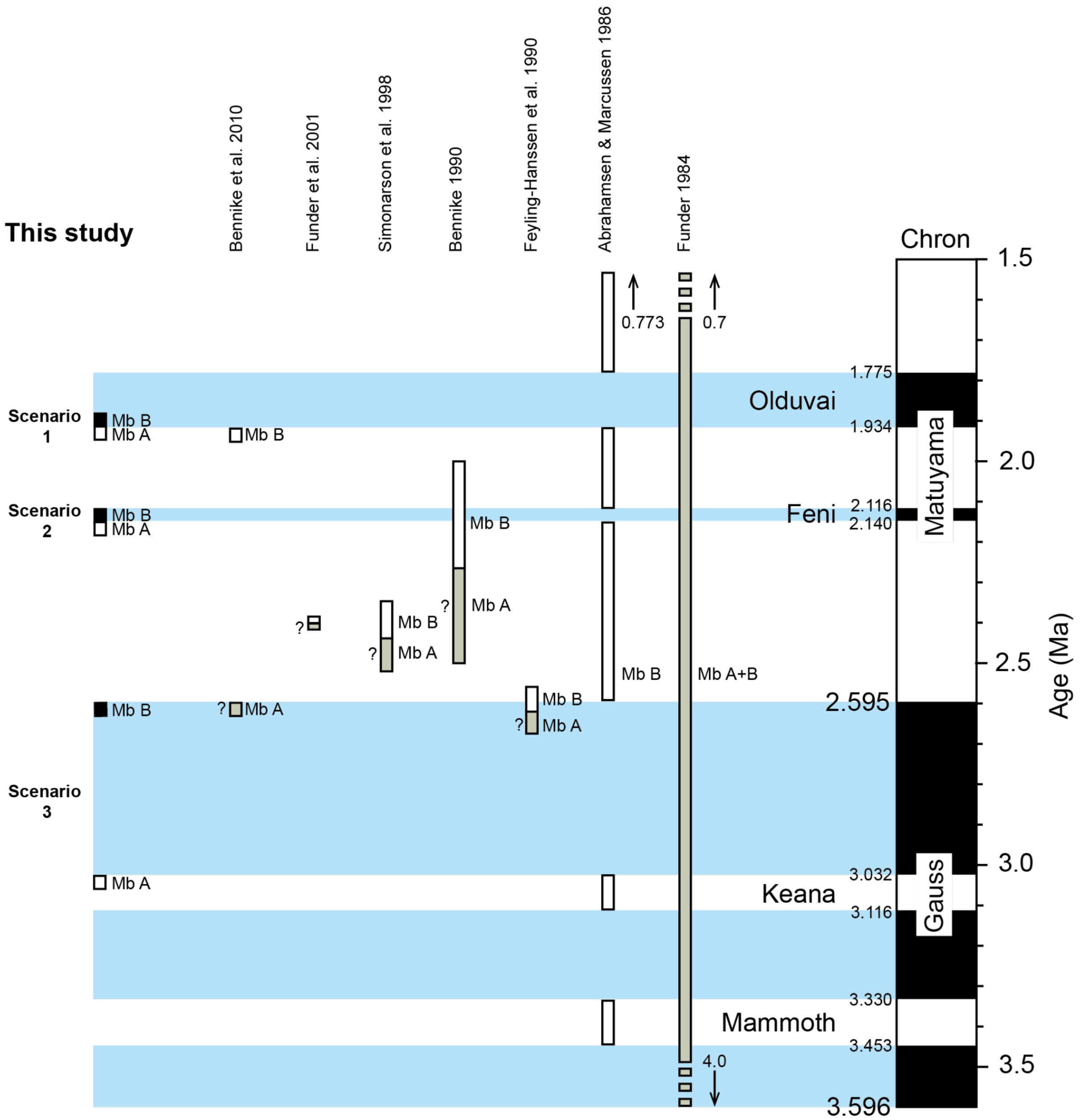

Fig. S2.1. Compilation of the previous age determinations of the Kap København Formation. Mb: Member.

Fig. S2.1. shows age estimates provided from previous studies of the Kap København Formation. Using paleomagnetic measurements in Member B and biostratigraphy the age of the Kap København Formation was tentatively suggested to be between 0.7 and 4.0 Ma[16,17]. Foraminifer stratigraphic analyses placed the upper part of the sequence (unit B2 at loc. 50) at the Late Pliocene to Early Pleistocene boundary[16,108]. Later, the Plio-Pleistocene age was reconfirmed i.e., at a relatively narrow interval across Pliocene-Pleistocene transition[19]. The presence of the foraminifera indicator species *Cibicides grossus* in Member A suggests an Early Pleistocene age as it became extinct at about 2.3–

2.5 Ma in Arctic Canada and East Greenland[109,110] (McNeil 1990). Combining, the different occurrences of small mammals, foraminifers, molluscs, ostracod assemblies and their long distance correlatives as well as paleomagnetism, Bennike (1990) (ref. [18]) concluded that the Kap København Formation was probably deposited between 2 and 2.5 Ma ago. Based mainly on first and last appearance datums for species found in the sediments and age restraints provided by the previous studies, Símonarson et al. (1998) (ref. [111]) correlated the lowermost diamict of Member A with the Praetiglian of northern Europe. Using the orbitally tuned oxygen isotope record it was further suggested that the Kap København Formation was deposited within isotope stage 100-91, which equals 2.52-2.34 Ma[14,112] . Consequently, Funder et al. (2001) (ref. [7]) estimated a sedimentation duration of about half of a 41,000-year obliquity cycle i.e 20,000 years for the entire glacial-interglacial deposition. Thus, the age estimate provided by Símonarson et al. (1998) (ref. [14]) was further confined to a consensus age of 2.4 Ma[7]. Some credibility for this narrower age bracket came from amino acid racemization in mollusc shells and the marine fauna containing the *Arctica islandica* mollusc (presently not in situ) and the extinct *Cibicides grossus* foraminifera[7]. The reasoning behind this interpretation is slightly complicated because these thermophilous fauna assemblies are found in a horizon above Member A sediments in an otherwise cold offshore glaciomarine environment. Thus, it was considered to be reworked[7,14]. On the other hand, amino acid ratios show that no large age difference seems to exist between the København Formation and the allochthonous fauna.

An exception to the other age studies of Kap København is the work by Bennike et al (2010) (ref. [105]). To cross-correlate Kap København with three other Early Pleistocene sediment sequences in Northeast and East Greenland, Members A and B were divided in time with a c. 700,000-year hiatus. Member A was assigned to the normal polarity at the beginning of Gauss at 2.6 Ma based on the foraminifera content, while Member B was placed immediately below the Olduvai subchron around 1.9 Ma ago. Besides matching the biostratigraphy between these sites, this age model is dependent on the paleomagnetic pattern - Member B being reverse polarity and a reinterpretation of the marine fauna of Member B becoming autochthonous. Thus, relying on the last appearance datum of the *Cibicides grossus* fauna in Member B older than 2.5 Ma. However, this re-interpretation is implicit and remains tentative as no argumentation is provided by Bennike et al (2010) (ref. [105]).

Our new paleomagnetic data show that Member A has reversed magnetic polarity and the main part of the overlying unit B2 has normal magnetic polarity. In the context of previous work, this is

consistent with several magnetostratigraphic matches, including the 2.58-Ma (Scenario 3), 2.14-Ma (Scenario 2) and 1.93-Ma (Scenario 1) options (Fig. S2.1). The late-Gauss (Scenario 3) interpretation of the paleomagnetic data implies that a long time has elapsed between the deposition of the reversed Member A and normal Member B (unit B2). We find no reason to question the evidence for a continuous unbroken sedimentation between Member A and B as given earlier[16] (Funder et al., 1984)[7,10,14,19]. No paleosols, increase in bioturbation intensity, or significant diagenesis consistent with a million-year stasis have been observed. Also, when the glacial-glaciomarine to shoreface sequence is emplaced during a single interglaciation, then a single interglaciation must coincide with a R-to-N succession. This is possible for the Olduvai and Feni subchrons, but not for the latest Gauss - a 2.58 Ma age for the normal unit would require that the lower unit be 0.4 Ma older to get to the Kaena, and that the sequence span several glacial-interglacial cycles. So, the paleomagnetic data by themselves are consistent with various ages but tend to suggest one of the younger options. A critical supporting factor is the last appearance datums of the mammals, foraminifera, and molluscs in the stratigraphic record.

We inspected the lagomorph fossils from Kap København and concluded that a single form, *Hypolagus* sp., is present in the material. It is indicated by the left P2 premolar with specific *Hypolagus* morphology with the hypercone of simple morphology without hypoflexus. The presumed *Lepus* bones[113], determined by Repenning (1987) (and referred to as "a fragment of a mandible") are in fact represented by fragmented parts of an upper cranial bones, maxilae and squamosum. One maxillar piece shows an alveolus for the premolar. The left P2 of *Hypolagus* fits the left alveolus for P2 making them a couple. Another identifiable piece of the same preservation is a fragment of a left jugal process of the squamosal bone. In fact, the Kap København lagomorph record likely represents a cranial fragment of a single individual that later or at the time of excavation fell apart and were placed under different genus names. The presence of only *Hypolagus* in the Member B sequence enables us to impose biochronological constraints on the enclosing deposits.

According to Bell et al., 2004, (ref. [114]) *Hypolagus* is generally found in the Blancan mammal stage prior to 2 Ma. The latest record of *Hypolagus* is reported from the Froman Ferry Formation section believed to be constrained between ca. 1.5 Ma - age of the overlying ash, and 1.77 Ma - top of Olduvai believed to be below[115,116]. However, because no external age control was provided for the reversely magnetised lower part of the section, this presumably youngest record remains ambiguous[115]. Martin

(2007) (in ref. [117]) argued the presumed age of the Froman Ferry Local Fauna was based on the assumption that the reversely magnetised sediments containing the fauna from beneath the Pickles Butte ash, dated at 1.58 Ma, were deposited within the middle of the Matuyama Chron during the Olduvai normal event was not recorded in sediments beneath the fossiliferous layers[115]. Thus, it remains very possible that the fossils were deposited earlier, perhaps during an early phase of the Matuyama Chron between 2 and 2.6 Ma. Given the lack of age control of the Froman Ferry Formation, the second youngest record of *Hypolagus* is Borchers locality with the age close to 2.0 Ma[115,118].

Concerning the age restriction imposed by the *Cibicides grossus* in Member A with a last appearance datum between 2.3-2.5 Ma, it is complicated by its possible survival until the end of the Olduvai ca. 1.8 Ma in the deeper waters of the northern North Sea, and off the coast of Norway[119,120]. It might very well be a time transgressive extinction beginning in the Arctic, terminating in the North Sea, which prevents a firm last appearance datum. In addition, it is difficult to use the last appearance of *Cibicides grossus* in Member A to determine the age as the fauna has been re-deposited.

3. Cosmogenic nuclide burial dating

Terrestrial cosmogenic isotopes (TCN) are produced in minerals when secondary cosmic radiation interacts with exposed elements such as Si and O in quartz. While hundreds of cosmogenic isotopes are produced, only a few with sufficiently slow decay rates and low non-cosmogenic natural abundances are suitable for measurement. The two most commonly used TCNs for burial dating are ^{10}Be (decay constant 4.997 ± 0.043) × 10^{-7} per year[121,122] and ^{26}Al (9.830 ± 0.250) × 10^{-7} (ref. [123,124]). The sea level high latitude (SLHL) production rates in quartz are 3.92, 0.012, and 0.039 atoms $g^{-1}a^{-1}$ for nucleonic, stopped muonic, and fast muonic ^{10}Be production respectively[125,126]. The SLHL production rates for ^{26}Al are 28.54, 0.84, and 0.081 atoms $g^{-1}a^{-1}$ for nucleonic, stopped muonic, and fast muonic production respectively[126]. These production rates are based on measurements from globally distributed calibration sites and have poorly constrained uncertainties. One estimate (^{10}Be 8.3% and ^{26}Al 7.1%) is the RMS error of the ratio of the exposure age using these production rates to the independently determined age at CRONUS calibration sites[126]. While production rates from a few high Arctic production rate calibration sites can be used, their departures from the global averages

are not significant and have not been explained in terms of atmospheric or magnetic field anomalies. Therefore, we have chosen to use the above global averages.

While at or near the surface, quartz is exposed to cosmic radiation while it is in solid bedrock, regolith, or sediments, or during transportation as sand in stream beds. The production ratio of ^{26}Al to ^{10}Be over hundreds of thousands of years of exposure is approximately steady at ~7 atoms/atoms (discussed below). The $^{26}Al/^{10}Be$ will decrease over time according to their decay rates. By comparing the initial assumed surface production ratio with the measured ratio after decay in the buried sample, the burial duration can be calculated[127]. The TCN production rates vary in complex ways[128] for each isotope with geomagnetic latitude, longitude, and elevation (atmospheric depth). Once the sediment is deposited in the near shore environment and buried under decametres of other sediment, water, or glacier ice, post-depositional TCN production in the sand grains is non-zero but small, contributing a small fraction of a percent of the concentrations at the time of deposition (by muogenic interactions only, at rates that are less than 3.1% and 1.2% of the total production of ^{26}Al and ^{10}Be at the surface). Therefore, we ignore any production between the time the sediment was buried offshore to the time it emerged from the water to its present location. To adjust for post-emergence production, and for all other burial calculations, we use the Lifton et al ([129]; referred to as 'SA' or 'LSD' scaling in some online calculators) approach, modified to include model 1A of Balco (2017)[130], a mean attenuation length for nucleons at 150 g cm^2 (ref. [131]), to scale from SLHL (spallogenic production rate for ^{10}Be at SLHL is 4.0 atoms g^{-1}yr^{-1}) to our actual sample sites in order to correct for any production since deposition and emergence. LSD is the same scaling method used by Borchers et al (2016) (ref. [126]) for normalising production rates from the global calibration dataset to SLHL. There is a relatively large (of order ~10%) uncertainty in the $^{26}Al/^{10}Be$ production ratio at the surface of the Earth. A widely used production ratio is 6.75 (atoms/atoms), the production rates from the CRONUS global dataset[126] yield a ratio of 7.42 (atoms/atoms), and recently a production ratio of 7.3 ± 0.3 (atoms/atoms) has been reported for Greenland[132]. As the jury is still out, we will use the commonly assumed 6.75 atoms/atoms. The effect of using 6.75 is that the ages we report may underestimate burial duration if the actual initial production ratio should be slightly higher. For comparison (details below), we recalculated the most probable burial age with the highest production ratio (7.42) and obtained ages that were only 0.2 Ma older than the ages using a ratio of 6.75, and within its 1σ uncertainty.

3.1 Field constraints on post-depositional exposure

While very little post-depositional production occurs when the sediment is buried under younger strata, water, or ice, there may be a non-trivial amount produced once the Kap København Formation isostatically emerged from the water. Therefore, we take the precaution to first characterise the possibility of post-emergence production by spallogenic and muogenic interactions, and then adjust the measured concentrations accordingly before calculating burial durations. The sediments dated are from Units 1 and 2 of Member B of Kap København Formation[7]. In stratigraphic order in a 90 m composite section, the marine sediments are interpreted to be a Late Pliocene to Pleistocene sequence of foreshore (KK06A), upper shoreface (KKI06B, KK01C, KK06C), and lower shoreface (KK05B, KK05A, and KK03B) facies. Samples were collected below naturally stream- or marine-cut terraces at different locations (Table S3.1.1, Fig. S3.1.1), making it difficult to know their exact individual history of post-emergence exposure.

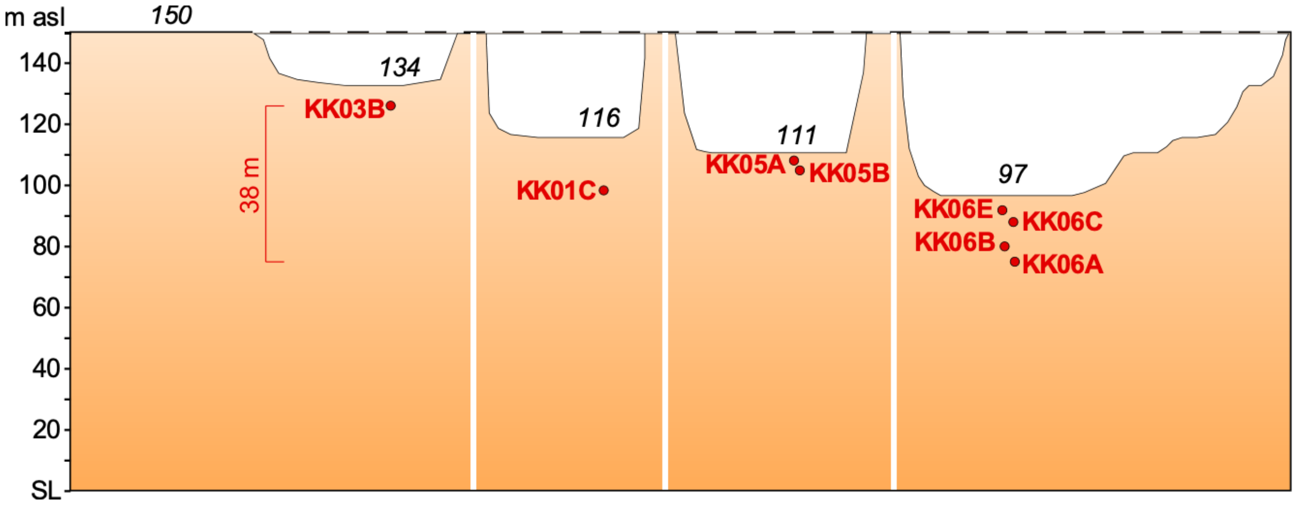

Fig. S3.1.1. Relative positions of the samples and their depth relative to the modern surface and the interpreted pre-incision surface (150 m dashed line). This is not a topographic profile. The four cut terraces are placed in order of decreasing elevation to aid in interpretation of the TCN data. Black numbers are elevations above SL, red dots are the eight samples with sample IDs, and the red bar is the range of depth between the highest sample (KK03B, 126 m asl) and lowest (KK06A 75 m asl). KK05A is the shallowest sample at 3 m below the 111-m terrace.

Their actual time-integrated elevation varied over >200 m, from their shielded depth many decametres below sea level at the time of deposition to their present elevation. Processes causing this elevation change may include regional glacio-isostatic cycles of subsidence and uplift over the past millions of years, isostatic uplift of the coast caused by local incision, and lithospheric flexure owing to sediment loading offshore. Additionally, 'eustatic' sea level has risen and fallen over dozens of glacial cycles since burial which may not be in phase with the regional processes. Surface uplift caused by tectonic or other dynamic topography processes is unconstrained. In summary, it is not possible to determine the actual time-averaged elevation and erosion history of these sediments over the entire post-depositional time. Fortunately, because many of the samples were sufficiently shielded under sediment, the effect of these elevation and shielding changes is minimized because the production rate at that shielded depth will be low (e.g., <10% of the muogenic production rate at the surface, which is only 3% (^{26}Al) or 2% (^{10}Be) of the total TCN production rate at the surface). Furthermore, the sediments were initially deposited some distance offshore in some uncertain water depth, for an unknown time, and also covered by ice sheet or shelf ice of unknown thickness during multiple glaciations since the sediment deposition. Those shielding masses (water and ice) further reduce the post-deposition production and therefore the effect of the uncertainty in elevation of sediments on the burial age uncertainty.

Table S3.1.1. Site Information

Field ID	Stratigraphic Position	Year Sampled	Latitude	Longitude	Sample depth	Surface elevation
		yr	d.d	d.d	m	m
KKO1C	Unit B1 Loc.69	2012	82.4942	-21.6061	18	116
KK03B	Unit B2 Loc. 119 above DNA	2012	82.496	-21.5591	8	134

KK05A-upper	Unit B2 Loc. 175 above DNA	2012	82.494	-21.4779	3	111
KK05B-lower	UnitB2 Loc. 175 below DNA	2012	82.494	-21.4779	6	111
KK06A	Unit B1 Loc.77 lower	2012	82.4961	-21.231	21	96
KK06B	Unit B1 Loc.77 middle	2012	82.4961	-21.231	17	97
KK06C	Unit B1 Loc.77 top	2012	82.4961	-21.231	9	97
KK06E	Unit B2 Loc.77 top	2012	82.4961	-21.231	4	96

However, a possible first-order factor controlling the TCN concentrations that we consider is that the samples were collected below naturally cut terraces of different elevations (134 to 97 m asl, Fig. S3.1.1). As the sediment was deposited in a shallow marine environment (based on sedimentary facies and marine fossils) it is likely that most of this incision was after their emergence (estuarine channels are possible but given the modern morphology most incision was post-emergence. The incision depths are deeper than the reach of post-emergence fast nucleon spallation production, but within the range of muogenic production. However, we do not know the relative timing of the incisions (for instance, they were not generated by a simple headward incision in one small drainage). There are some constraints. First, Independence Fiord was glaciated during the LGM, based on a till immediately under dated Holocene marine sediments[133]. So, it is quite likely that the samples were shielded under the Greenland Ice Sheet until the Holocene. We cannot preclude the possibility that the samples were exposed during the penultimate or pre-penultimate interglacial, but it would be almost certain that the samples would have been deeper during those times and exhumed to a shallower depth during each glaciation. The local marine limit was only about 70 m.a.s.l. (ref. [134]), so it appears that marine erosion of the Kap København Formation is unlikely. In summary, the differential amounts, and timings of the incisions below which the TCN samples were collected may have contributed to variability of TCN concentrations at different sites, and therefore challenges the interpretation of the data. If a terrace has been exposed for many tens of thousands of years, but other terraces for less than 10 ka, the terrace that has been exposed longer will have a much higher ^{26}Al/^{10}Be (owing to the faster production rate of ^{26}Al) and appear to have a younger burial age. To evaluate the impact of these factors, we have completed a series of sensitivity analyses, including *depth profiles* at sites with at least three samples. If the concentrations of ^{10}Be systematically decrease with depth at a given site, this reveals that post-emergence and post-incision exposure has occurred and the measured concentrations need to be adjusted accordingly.

3.2 TCN analytical methods

Quartz rich sand was collected at multiple field sites at current depths of 3.0 to 21.0 m, after excavating ca. 30 cm horizontally into the bedded sediments. The following accelerator mass spectrometry (AMS) target chemistry procedure was completed at the Cosmic ray isotope sciences @ Dalhousie (CRISDal) Lab. Mineral separation using combinations of heavy liquids, froth floatation, Frantz magnetic separation, and partial digestions in aqua regia or HF ultrasonic baths continued until abundances of native Al in the quartz concentrate were below 100 ppm Al and Ti (as determined on 0.5 g of sand aliquots using ICP-OES) and the quartz concentrate appeared pure under an optical microscope. Type 2 water (RO with some deionization) was used for the previous steps and Type 1 (18.2 MOhm) deionized water was used for all subsequent steps including acid cleaning of any labware and target holders, using an Elga Centra-R60 water purification system with continuous recirculation to the dispensing sites, where there are additional mixed-bed resin columns to ensure 18.2 MOhm, and for the ^{26}Al and ^{10}Be lab a milli-Q boron filter and final milli-Q mixed-bed resin column. Following the quartz purification procedure, we removed an additional 30 to 35 wt% of the quartz to remove any meteoric ^{10}Be still attached on the surface or on microfractures in the quartz grains[135]. We used a high quartz mass in this experiment (e.g., 80 g, quadruple the typical quartz masses of 15-25 g for exposure dating) for two reasons. We were uncertain if the burial duration could be several millions of years (i.e., more than 6 half-lives of ^{26}Al). We were concerned that the erosion rate in the paleo-catchments would be so rapid (consistent with the volume of sediment in the KK formation) that the depositional concentrations of the TCN would be low at the outset of burial. The larger quartz mass would help provide sufficient ^{26}Al to improve AMS counting statistics, while the ^{26}Al/^{27}Al ratio would not change. We used the following isotope dilution method to produce mg-size targets of BeO and Al_2O_3 for AMS measurements of the ^{10}Be/^{9}Be and ^{26}Al/^{27}Al ratios). The quartz was desiccated at room temperature, massed, and spiked gravimetrically with ca. 220 µg Be from the carrier solution. The Be carrier "Be Carrier B31 Sept 28, 2012" was produced at CRISDal from phenacite sourced from the Ural Mountains, with an ICP-OES-measured average Be concentration of 282 ± 5.64 µg/ml (replicated by N. Lifton at PRIME Lab with a measurement of 279 µg/ml, Table 3.2.1) and density of 1.013 g/ml. We digested the mixture with the minimum needed volumes of concentrated trace-metal grade aqua regia, HF, and perchloric acid. In addition to its strong oxidising potential to help in digestion, perchloric acid has the benefit of a high boiling temperature to allow more efficient evaporation of the fluorosilicic acid and remaining HF and forms a perchlorate cake upon drydown that is more manageable for the subsequent dissolution, which then

was followed by a second perchloric evaporation and drydown. The cake was dissolved and brought up to 100 ml in 2% ultrapure nitric acid, and we extracted a 5 ml aliquot to measure the Al and Be on the actual solution used for target chemistry. The 5-ml aliquots indicated that the samples were similar in Al, Be, and Ti concentration, with Al = 14.1 ± 2.0 µg/ml (s.d.), Be = 0.33 ± 0.02 µg/ml, and Ti = 3.1± 1.8 µg/ml (duplicate measurements on completely separate aliquots of the same sample had precisions better than 1%). The Al spike used for the blank was an Alfa Aesar 1000 µg/ml ICP-MS standard with a density of 1.010 g/ml. Because the native Al concentration in the quartz was relatively large (100-200 µg/g) and because we used a large quartz mass, we did not need to spike the samples with Al to produce the desired 4 mg of Al_2O_3 for the CAMS-LLNL target and ion source. To compensate for the large mass of Al, we used a purposefully modified elution procedure. After an anion column chemistry which mainly removes Fe as an $FeCl^-$, we employed an elution experiment for the cation chemistry that uses a larger bed volume of resin than typical 2 or 5 ml procedures (10 ml of AG-50W-X8-200-400 mesh) and we used CRISDal elution procedure 2013-06-Elut-High which was previously optimised to separate Be and Al from a high Al mass solution, by calibrating the procedure with a cocktail containing 10,000 µg Al, 220 µg Be, and 10,000 µg Ti in 0.5 M HCl. The separated Be and Al chloride solutions were centrifuged, converted to $Be(OH)_2$ or $Al(OH)_3$ using ammonia gas, and then calcined to BeO and Al_2O_3 with a bunsen burner (3 minutes minimum) and furnace (4 hrs at 950°C) respectively. The targets were massed, carefully powdered in their boron-free quartz vials in a static-free glovebox, and then mixed well with the appropriate volumetric ratio of Nb:oxide (e.g. the Nb:Al_2O_3 was between 1:3 to 1:4 as recommended by R. Finkel), packed tightly to the optimal height for Cs-sputtering in thoroughly cleaned stainless steel target holders, and then kept in a desiccator until couriered. All AMS measurements were completed at the Centre for AMS at Lawrence Livermore National Lab (CAMS-LLNL) (both the $^{26}Al/^{27}Al$ and $^{10}Be/^{9}Be$ measurements were in March 2014). The AMS standards used for normalisation of the sample results were KNSTD 30960 (ref. [123]) with $^{26}Al/^{27}Al$ of 3.096 × 10^{-11} atoms/atoms and 07KNSTD3110 (ref. [136]) with $^{10}Be/^{9}Be$ of 2.85 × 10^{-12} at/at. The average $^{10}Be/^{9}Be$ for the phenacite spike has been 1 to 20 × 10^{-16} at/at, with a higher ratio observed on samples with large quartz masses that require greater acid volumes for digestion and therefore longer evaporation times. In 2014, Be targets were analysed without a post-stripping foil so required small corrections for boron isobaric interferences (live times ranged from 97.7% to 98.8%). Native Be concentrations in the quartz were at the limit of ICP-OES detection (ranged from 0 to 0.03 µg/g), so the mass of spike Be is considered the mass of all ^{9}Be. The Al process blank generated $^{26}Al/^{27}Al$ ratios of 2 to 4 × 10^{-15}.

Table S3.2.1. Geochemical data for $^{26}Al/^{10}Be$ dating including stable isotope concentrations

Field ID	CRISDal Lab ID	Qtz Mass	Be Carrier Mass	^{9}Be carrier atoms added	^{27}Al in qtz	^{27}Al in tgt
		g	g	x 10^{19} at	mg	x 10^{19} at
P. Blank	2914	0	0.9281	1.7264	--	6.546
KK01C	2967	84.1738	0.8903	1.6561	10.151	22.66
KK05AU	2968	80.3801	0.8935	1.6621	9.542	21.30
KK05BL	2969	80.6573	0.8959	1.6666	10.275	22.93
KK06A	2970	80.5319	0.8956	1.6660	9.889	22.07
KK06B	2971	86.6866	0.9052	1.6839	9.583	21.39
KK06C	2972	82.3449	0.8965	1.6677	8.092	18.06
KK06E	2974	74.0618	0.8933	1.6617	11.054	24.67
KK03A	2975	80.1602	0.8988	1.6719	8.629	19.26
KK03B	2976	83.6507	0.8860	1.6481	7.852	17.53

Table S3.2.1 Notes:

Table Abbreviations: P. Blank (process blank), Qtz (quartz), at (atoms), tgt (target)

The mass of ^{27}Al carrier added for the P. Blank was 2.9633 g.

Mass of ^{27}Al and ^{9}Be in quartz was measured using 0.5 g aliquot of qtz for each sample, digested in HF, dried, and brought up in 2% HNO_3 acid, measured by ICP-OES. ICP-OES measurement errors averaged 2%, with optimization on Al, using major element matrix-matched standards that ranged from 0.05 to 30 µg/ml for Al and 0.01 to 3 µg/ml for Be.

Table S3.2.2. AMS results for $^{26}Al/^{10}Be$ dating.

Field ID	$^{10}Be/^{9}Be$ in tgt	1σ Unc $^{10}Be/^{9}Be$	**^{10}Be in qtz**	1σ Unc ^{10}Be in qtz	$^{26}Al/^{27}Al$ in tgt	1σ Unc $^{26}Al/^{27}Al$	**^{26}Al in qtz**	1σ Unc ^{26}Al in qtz	**$^{26}Al/^{10}Be$**
	x 10^{-15} at/at	x 10^{-15} at/at	**x 10^{4} at/g**	%	x 10^{-15} at/at	x 10^{-15} at/at	**x 10^{4} at/g**	%	**at/at**
P. Blank	1.435	1.428	**--**	--	2.446	1.316	--	--	
KK01C	59.61	3.95	**1.144**	7.36%	6.793	1.659	**1.64**	25.1%	**1.43**
KK05AU	61.21	2.64	**1.235**	5.33%	15.118	2.675	**3.81**	18.0%	**3.09**
KK05BL	61.41	1.85	**1.238**	4.34%	7.254	1.737	**1.86**	24.6%	**1.50**
KK06A	57.40	1.75	**1.157**	4.46%	10.644	2.786	**2.72**	26.5%	**2.35**
KK06B	63.31	2.33	**1.201**	4.78%	10.245	2.505	**2.34**	24.8%	**1.95**
KK06C	64.39	1.93	**1.274**	4.27%	13.551	2.678	**2.78**	20.2%	**2.18**
KK06E	70.16	1.77	**1.541**	3.85%	21.876	2.923	**7.07**	13.6%	**4.59**
KK03A	63.99	1.89	**1.304**	4.25%	*n.d.*	*n.d.*	***n.d.***	*n.d.*	***n.d.***
KK03B	67.01	1.57	**1.291**	3.80%	19.102	3.146	**3.81**	16.8%	**2.95**

Table S3.2.2 Notes:

P. Blank or BLK is the process blank, an estimate of ^{10}Be or ^{26}Al contamination during all stages of target preparation, in containers without quartz.

The ^{10}Be and ^{26}Al concentrations in quartz (C) is the radionuclide/stable nuclide (R/S) AMS measurement, multiplied by the number of stable atoms in each target, minus the number of stable nuclide atoms in the blank, divided by the mass of quartz:

$$C = \frac{\left[\left(\frac{R}{S}\times S\right)-blank\right]}{massqtz}.$$

Measurement uncertainty: The total analytical uncertainty in the R concentration per g qtz is a combination of the following random uncertainties added in quadrature:

$UNC = ((AMSunc)^2 + (Carrunc)^2 + (Blkunc)^2)^{0.5}$

*The 1σ AMS uncertainty (**AMSunc**) for the radionuclide/stable nuclide (R/S) measurements are the greater of the Poisson distributed statistic for the total number of counts on a target or the coefficient of variation in the R/S about the mean of the three, four, or five analytical passes on each target. The uncertainty in carrier concentration (**Carrunc**) for Be carrier is based on multiple measurements of Be over years, including measurements by other facilities who used the carrier, and averages 2.2%. Although the commercial Al standard used for the Al carrier has a better uncertainty than this, we assume the 2.2% uncertainty for the Al in order to consider uncertainties in the gravimetric measurements during chemistry. The **Blkunc** is the product of the P.Blank AMSunc measurement (99.5% for $^{10}Be/^{9}Be$ and 53.8% for $^{26}Al/^{27}Al$) and the fraction of the P. blank relative to each sample measurement:*

$Blkunc = \left[(AMSunc_{Blk}) \times \frac{atomsN_{Blk}}{atomsN_{Spl}}\right]$ *and averaged 2.3% for ^{10}Be and 3.6% for ^{26}Al.*

The largest contributor to the uncertainty in the AMS measurements was the measurement of $^{26}Al/^{27}Al$ at the 10^{-14} and 10^{-15} levels. As requested by a reviewer, we provide a summary of the ^{27}Al current during the $^{26}Al/^{27}Al$ measurement run in March 2014 (Table S3.2.3) which reveals that the targets pulled a current comparable to that of the pure ^{26}Al standards, suggesting that the targets were reasonably pure Al_2O_3 and loaded properly, and consistent with the fact that the uncertainty was observed to be controlled by Poisson counting statistics, and not inter-pass variability owing to target purity issues.

Table S3.2.3. Average currents of the stable $^{27}Al^-$ ion measured over each 600-second pass

Field ID	Lab ID	AMS ID	Evolution of average current during analysis (27Al- µA)			
			Pass1	Pass2	Pass3	Pass4
**KK01C**	JG2967	AL13166	1.27	1.65	1.73	
**KK05A upper**	JG2968	AL13167	1.30	1.53	1.41	
**KK05 B lower**	JG2969	AL13168	1.59	1.18	0.86	0.90
**KK06A**	JG2970	AL13169	1.18	0.96	0.62	

**KK06B**	JG2971	AL13170	1.60	0.94	0.74	
**KK06C**	JG2972	AL13171	1.67	1.25	0.87	
**KK06E**	JG2974	AL13172	1.75	1.79	1.61	
**KK03B**	JG2976	AL13174	1.42	1.32	1.12	
**Al blank, no Be**	JG2986	AL13161	1.42	0.99	0.57	0.57
AMS standard	KNSTD10650	AL13221	1.58	1.42	1.28	0.71
AMS standard	KNSTD10650	AL13222	1.51	1.47	0.76	0.62
AMS standard	KNSTD30960	AL13226	1.41	1.38	1.21	1.18
AMS standard	KNSTD30960	AL13227	1.27	1.28	1.30	1.06
AMS standard	KNSTD30960	AL13228	1.53	1.30	1.24	1.07
AMS standard	KNSTD30960	AL13229	1.53	1.40	1.38	1.40
AMS standard	KNSTD30960	AL13230	1.22	1.06	1.13	1.16
AMS standard	KNSTD30960	AL13231	1.24	1.17	1.21	1.17
AMS standard	KNSTD30960	AL13232	1.06	1.06	1.12	1.08
AMS standard	KNSTD30960	AL13233	1.29	1.19	1.17	1.11
AMS standard	KNSTD4694	AL13219	0.80	1.23	1.00	0.94
AMS standard	KNSTD4694	AL13220	1.12	1.28	1.30	0.87
AMS standard	KNSTD480	AL13130	0.53	0.67	0.54	0.53
AMS standard	KNSTD480	AL13211	0.78	0.58	0.49	0.48
AMS standard	KNSTD74440	AL13159	0.74			
AMS standard	KNSTD74440	AL13243	1.32	1.33	1.25	1.40

3.3 Simple burial age calculation

We used a Matlab code[137] to determine the burial age that best fitted concentrations of ^{10}Be and ^{26}Al given a simple single surface build-up and simple single burial history. Spallogenic production, muon production, and scaling is based on ref[129] ('LSD scaling scheme') including during the build-up period, the burial period, and the post-emergence period. Results are presented in Table S3.3.1, Table S3.3.2. As discussed in the main text, we use a ^{10}Be production rate of 4.0 atoms g^{-1} yr^{-1} and ^{26}Al/^{10}Be of 6.75 unless otherwise stated (as a sensitivity test, we show the results with ^{26}Al/^{10}Be of 7.42 including for adjustments to the concentrations related to recent incision). For muogenic production, we assign alpha = 1, as per Balco, 2017 (ref. [130]). For the calculations we use two shielding scenarios:

(1) present-day depths; and (2) the depths from the emergence model described in paragraph 3.1 that assumes the samples were under a 150 m terrace for most of their post-emergence time and that their depths were much deeper than present day. However, in both cases, allowing post-emergence production by spallation and muons resulted in TCN model concentrations that were significantly greater than those measured. This indicates that there was a significantly greater depth and duration of shielding than is apparent today, possibly owing to shielding by Greenland Ice Sheet cover (see paragraph 3.2) or recent erosion of a significant mass (tens of metres) of the Kap København Formation, or both. Table S3.3.1B provides the most probable burial age ± 1σ uncertainty for the case that the samples have remained in the same position for a long time (i.e., that the incision was a long time before the Holocene). The burial ages range from 0.81 to 3.21 Ma and individually would be interpreted to be a maximum estimate of burial duration for the Kap København Formation because it is not certain that only one burial event is recorded by each sample. The fact that the ^{10}Be concentrations are very similar, except for KK06E, would suggest that significant differences in burial histories among the samples is unlikely, and such uniform concentrations are consistent with very deeply shielded samples. The apparent lack of any significant storage space between the coastal sample site and the nearby mountainous source region also is consistent with brief, if any, storage episodes for the sand prior to final deposition in the Kap København Formation. Nevertheless, we will interpret the individual burial ages as maxima.

Table S3.3.2 is for the case that the surface of the Kap København Formation in the sampled area was 150 m asl until very recently, and that the channels were incised instantaneously just prior to sampling (i.e. that for most of the time the samples were much deeper and more shielded than today). The range of the ages is again 0.81 to 3.21 Ma (Table S3.3.2B, with $^{26}Al/^{10}Be = 6.75$) with very little change in any of the burial ages. This is important because it reveals that the history of incision does not affect the individual burial ages significantly. This insensitivity also holds if we use the maximum $^{26}Al/^{10}Be = 7.42$ (Tables S3.3.1C and S3.3.2C).

Table S3.3.1 These burial ages are based on the environmental and isotope data as shown, assuming that the landscape today has been constant for hundreds of thousands of years. A: Parameters for the burial age calculation. B: Burial age and initial catchment exposure age based on $^{26}Al/^{10}Be = 6.75$. C: Same, except $^{26}Al/^{10}Be = 7.42$

A. Parameters

Sample ID	depth (cm)	density (g/cc)	lat (dec)	long (dec)	elev (m)	Be (atoms/g)	unc Be (atoms/g)	Al (atoms/g)	unc Al (atoms/g)
KK01C	1800	1.9	82.4942	-21.6061	116	11435	842	16382	4119
KK03B	800	1.9	82.4960	-21.5591	134	12907	490	38110	6397
KK05AU	300	1.9	82.4940	-21.4779	111	12350	658	38065	6854
KK05BL	600	1.9	82.4940	-21.4779	111	12382	537	18642	4582
KK06A	2100	1.9	82.4961	-21.231	97	11569	516	27187	7207
KK06B	1700	1.9	82.4961	-21.231	97	12013	574	23431	5821
KK06C	900	1.9	82.4961	-21.231	97	12741	544	27780	5604
KK06E	400	1.9	82.4961	-21.231	97	15408	593	70719	9620

B. $^{26}Al/^{10}Be = 6.75$

Sample ID	Estimated Depth (m)	Most Probable Age (Ma)	1σ+ (Ma)	1σ- (Ma)	Min. Exp. (ka)
KK01C	18	3.21	0.86	0.39	11.6
KK03B	8	1.71	0.47	0.28	6.1
KK05AU	8	1.62	0.52	0.31	5.7
KK05BL	3	3.11	0.82	0.36	12.0
KK06A	21	2.19	0.95	0.38	7.1
KK06B	17	2.58	0.84	0.37	9.0
KK06C	9	2.35	0.60	0.33	8.5
KK06E	4	0.81	0.35	0.25	4.8

C. $^{26}Al/^{10}Be = 7.42$

Sample ID	Estimated Depth (m)	Most Probable Age (Ma)	1σ+ (Ma)	1σ- (Ma)	Min. Exp. (ka)
KK01C	18	3.39	0.86	0.39	12.7
KK03B	8	1.91	0.46	0.29	6.7

KK05AU	8	1.82	0.51	0.32	6.2
KK05BL	3	3.31	0.81	0.37	13.1
KK06A	21	2.38	0.95	0.38	7.8
KK06B	17	2.76	0.84	0.37	9.9
KK06C	9	2.53	0.60	0.32	9.3
KK06E	4	0.99	0.36	0.25	5.2

Table S3.3.2 $^{26}Al/^{10}Be$ burial ages, assuming they are from below a single raised marine terrace that was originally 150 m asl, and that any erosion or incision above a sample was *recent* and *instantaneous*. The measured concentrations used have not been adjusted for post-emergence production. The sample depth used is the modern depth (m) plus the difference between the elevation of the surface below which the sample was collected and the 150 m terrace surface (*c.f.* Table S3.1.1 and Fig. S3.1.1). Min. Exp. is the minimum exposure duration in the catchment source area to derive the ^{10}Be concentration; there may have been multiple long exposures separated by partial burial events, so this value can significantly underestimate the total exposure history. **A**: Parameters for the burial age calculation. **B**: Burial age and initial catchment exposure age based on $^{26}Al/^{10}Be = 6.75$. **C**: Same, except $^{26}Al/^{10}Be = 7.42$

A. Parameters

Sample ID	depth (cm)	density (g/cc)	lat (dec)	long (dec)	elev (m)	Be (atoms/g)	delBe (atoms/g)	Al (atoms/g)	delAl (atoms/g)
KK01C	5200	1.9	82.4942	-21.6061	150	11435	842	16382	4119
KK03B	2400	1.9	82.4960	-21.5591	150	12907	490	38110	6397
KK05 BL	4500	1.9	82.4940	-21.4779	150	12382	537	18642	4582
KK05AU	4200	1.9	82.4940	-21.4779	150	12350	658	38065	6854
KK06A	7500	1.9	82.4961	-21.231	150	11569	516	27187	7207
KK06B	7000	1.9	82.4961	-21.231	150	12013	574	23431	5821
KK06C	6200	1.9	82.4961	-21.231	150	12741	544	27780	5604
KK06E	5800	1.9	82.4961	-21.231	150	15408	593	70719	9620

B. $^{26}Al/^{10}Be = 6.75$

Sample ID	Estimated Depth (m)	Most Probable Age (Ma)	1σ+ (Ma)	1σ- (Ma)	Min. Exp. (ka)
KK01C	52	3.21	0.86	0.39	11.2
KK03B	24	1.72	0.46	0.29	6.0
KK05AU	45	1.63	0.51	0.32	5.5
KK05BL	42	3.11	0.81	0.37	11.5
KK06A	75	2.19	0.95	0.38	6.8
KK06B	70	2.58	0.83	0.37	8.6
KK06C	62	2.35	0.60	0.33	8.1
KK06E	58	0.81	0.35	0.25	4.5

C. $^{26}Al/^{10}Be = 7.42$

Sample ID	Estimated Depth (m)	Most Probable Age (Ma)	1σ+ (Ma)	1σ- (Ma)	Min. Exp. (ka)
KK01C	52	3.39	0.86	0.39	12.3
KK03B	24	1.91	0.46	0.29	6.6
KK05AU	45	1.82	0.50	0.32	6.0
KK05BL	42	3.30	0.82	0.37	12.7
KK06AU	75	2.38	0.95	0.38	7.5
KK06BL	70	2.76	0.84	0.37	2.8
KK06C	62	2.53	0.60	0.32	8.9
KK06E	58	1.00	0.35	0.25	5.0

A quick review of ^{10}Be or ^{26}Al concentrations and burial ages in Tables S3.3.1 and S3.3.2 reveals that the concentrations and maximum ages are not equivalent across the four different sites. This can be caused by chemistry or AMS error or is the variation owing to complex exposure/burial histories for each sample site. A closer inspection of the data from the two terraces with multiple samples (KK05 at 111 m and KK06 at 97 m, Fig. S3.1.1) reveals that the variation can be explained by differences in post-incision exposure histories.

At KK05, the two ^{10}Be samples have equivalent concentrations, but the two ^{26}Al are significantly different. This is predicted in the instance that the post-incision history is short. The

depth of the two samples is 3 and 6 m. Production rates at this depth and elevation are a mixture of muonic and spallogenic production rates, and at about 3 m the two pathways have about the same production rate. For instance, the production rates of ^{10}Be by spallation & muons for the 3 m and 6 m samples, respectively are 0.107 & 0.1020 and 0.002 & 0.072 atom g^{-1} yr^{-1}, which means that even after 10 ka of exposure the two samples would still be within their ~5% 1σ internal analytical error. Thus, the ^{10}Be isotope is insensitive to short (<30 ka) exposure at these depths as most (95%) of the concentration can be inherited since initial deposition. On the other hand, the ^{26}Al concentration of the shallower sample is much higher than the deeper (i.e., the right direction to explain their age discrepancy). Assuming that the concentration of the deeper is entirely inherited from the initial depositional concentration (see depth profile analysis below), the differences in these two concentrations can be explained if the terrace was cut about 37 ka ago. Exposure of an incised terrace for 22 kyr can explain the ^{10}Be and ^{26}Al data within 2σ of their measurements at site KK05.

At KK06, a very similar pattern occurs with four samples spanning 4 to 21 m depth. Note that at this location sample 'A' is deepest. Except for KK06E, the ^{10}Be concentration does not vary significantly with depth (lowest sample is 25% lower than the uppermost) relative to the significant variation in ^{26}Al concentration with depth (lowest sample is 62% lower than the uppermost). Again, this implies that more of the ^{26}Al concentration was produced post-incision, owing to greater spallogenic production for shallower samples. The concentrations of both TCN decrease exponentially from top to bottom, suggesting that the exposure duration of this terrace may have been longer than terrace KK05. As we have no geological or other basis to establish the differences in incision histories above each sample site, we will just acknowledge that there is a sensitivity to post-depositional exposure of the uppermost samples (perhaps upper 6 m) which may contribute to the variability in simple burial age among the samples within a site and across different sites. The best geological explanation for KK06E to have such a much higher ^{10}Be and ^{26}Al concentration than the other samples is that it is possible that KK06E, which is one of the shallowest samples (presently 3 m deep) was deposited at a significantly different time (different inherited pre-depositional concentration) from the other sediments sampled in the Kap København Formation, or that KK06E was sampled from a recently deposited fill terrace which is above the cut terrace under which KK06A,B,&C were collected (Fig S3.1.1; today the terrace is at 97 m, but possibly the cut terrace was at ≤93 m and > 3 m was deposited as fill recently to bring it to 97 m).

The most probable minimum burial age from all the samples can be calculated from the convolved probability distributions of the data from Table S3.3.1. The most probable age of all eight

samples, assuming steady state erosion and using a production ratio of 6.75 and 7.42, is 2.409 ± 0.237(0.159) Ma or 2.553 ± 0.238(0.159) Ma respectively (Fig. S3.3.1). However, the considerable scatter among the individual pdfs may suggest the individual burial ages are not dating the same event (i.e., the depositional age of the Kap København Formation).

(a)

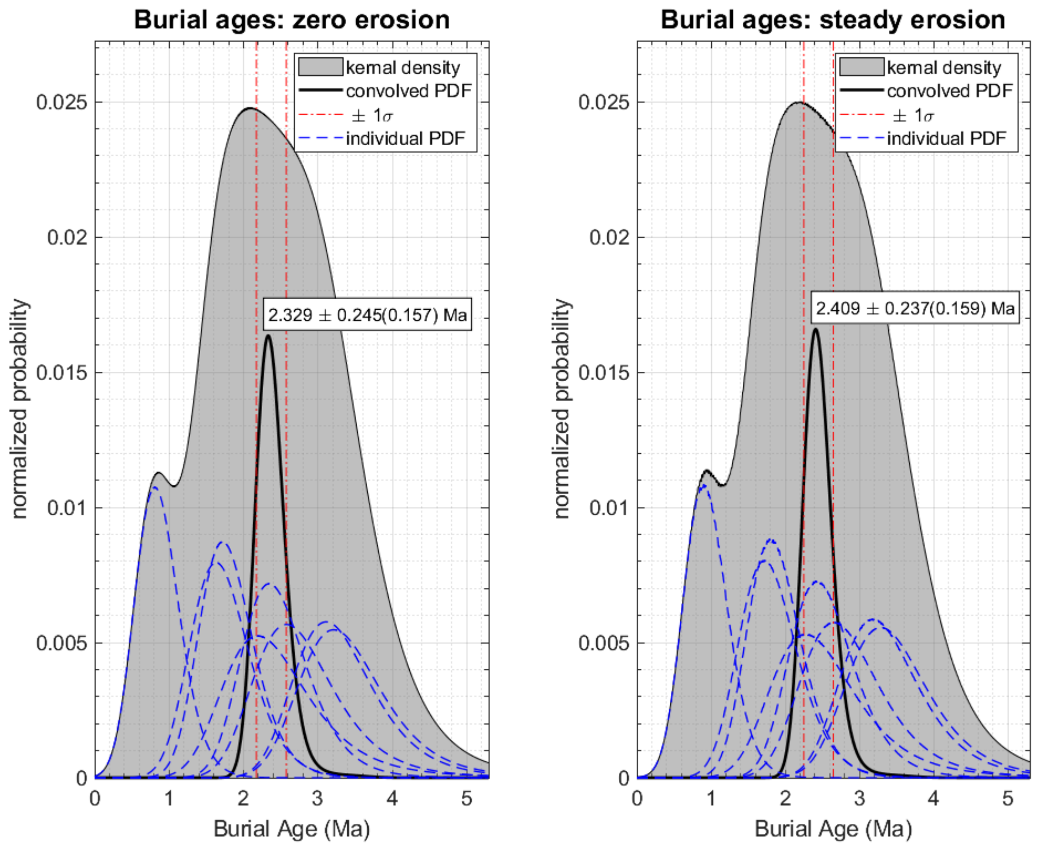

(b)

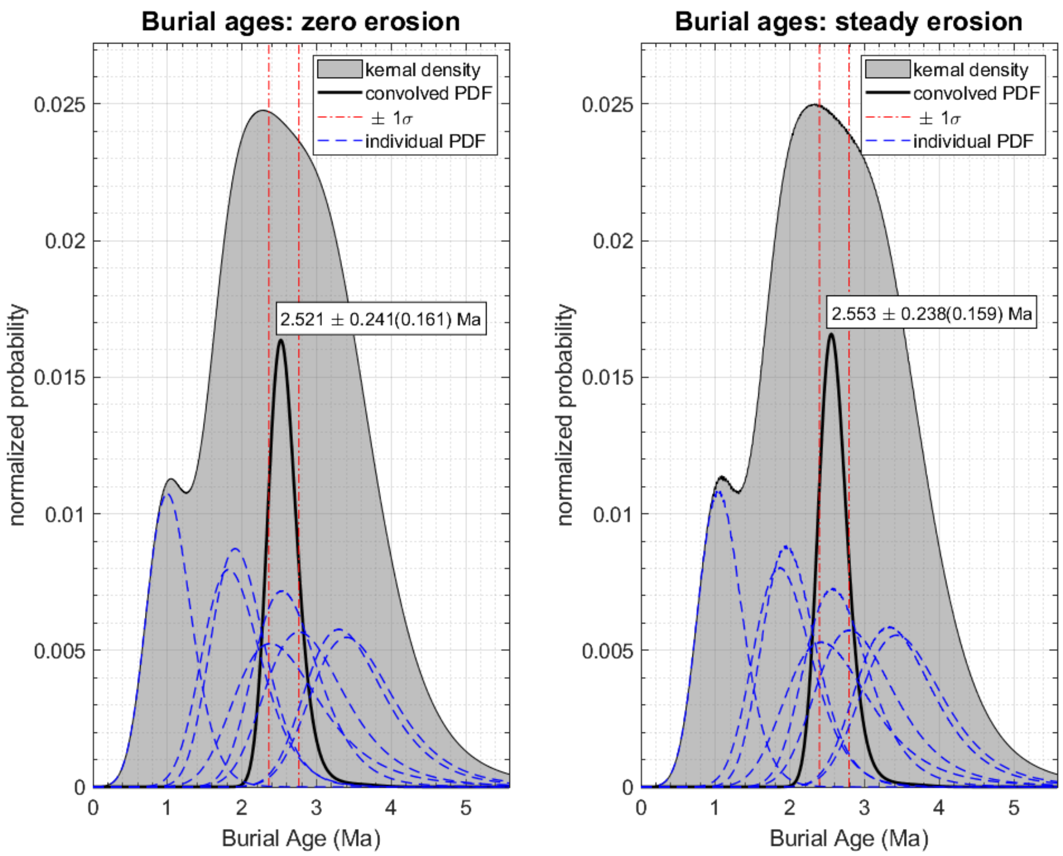

Figure S3.3.1. Convolved burial age of all eight samples in Table 3.3.1 assuming $^{26}Al/^{10}Be$ production ratio of 6.75 **(a)** and 7.42 **(b)**. Plots on the left assume production during build-up phase with zero surface erosion and plots on the right assume steady state erosion of catchment surfaces. The thick curve represents the convolved probability distribution function (multiplication of the probabilities of the three samples). The red dashed lines represent the 1s uncertainty for the most probable age. The blue dashed lines are the probability distributions for the individual samples. The shaded area represents the kernel density (the sum of the individual probabilities density functions) and is only shown as it is commonly used by others to represent the average age of a dataset.

3.4 TCN concentration depth profiles

We use a depth profile calculator[138] to plot the measured (unadjusted) concentrations for both ^{10}Be and ^{26}Al in a depth profile, assess their variability with depth, and model approximate exposure ages and nuclide inheritance values. Because the samples did not come from the same location, the depth profiles are a composite profile. We assume that each sample was below the 150 m terrace height. The surface production rates at this location for the different spallogenic production ratios are provided in Table S3.4.1, with magnetic field effects integrated over 2.0 Ma.

Table S3.4.1 Production rates at 150 m elevation for the different spallation production ratios.

**$^{26}Al/^{10}Be$ = 6.75**	^{10}Be	^{26}Al
Interaction	atom/g/yr	atom/g/yr
Nucleonic spallation	4.947	33.35
Total muonic production	0.149	1.25
Total production	5.096	34.60

**$^{26}Al/^{10}Be$ = 7.42**	^{10}Be	^{26}Al
Interaction	atom/g/yr	atom/g/yr
Nucleonic spallation	4.947	36.66
Total muonic production	0.149	1.25
Total production	5.096	37.91

The ^{10}Be depth profile (Fig. S3.4.1) for all eight samples using measured (unadjusted) concentrations reveals that there is a possibility that the sediments were exposed since the last glacial maximum (Table S2.4.2), with a most probable exposure age of 14.6 (+25.2, -14.6) ka based on a 5σ fit to all data. The most probable inheritance concentration (Fig. S3.4.2), i.e., prior to any post-glacial exposure, is 1.15 x 10^4 atoms/g (Table S3.4.2). Exposure ages and inheritance concentrations are derived from n=100,000 stochastically generated profile solutions allowing surface erosion rates between 0 and 3 cm/ka.

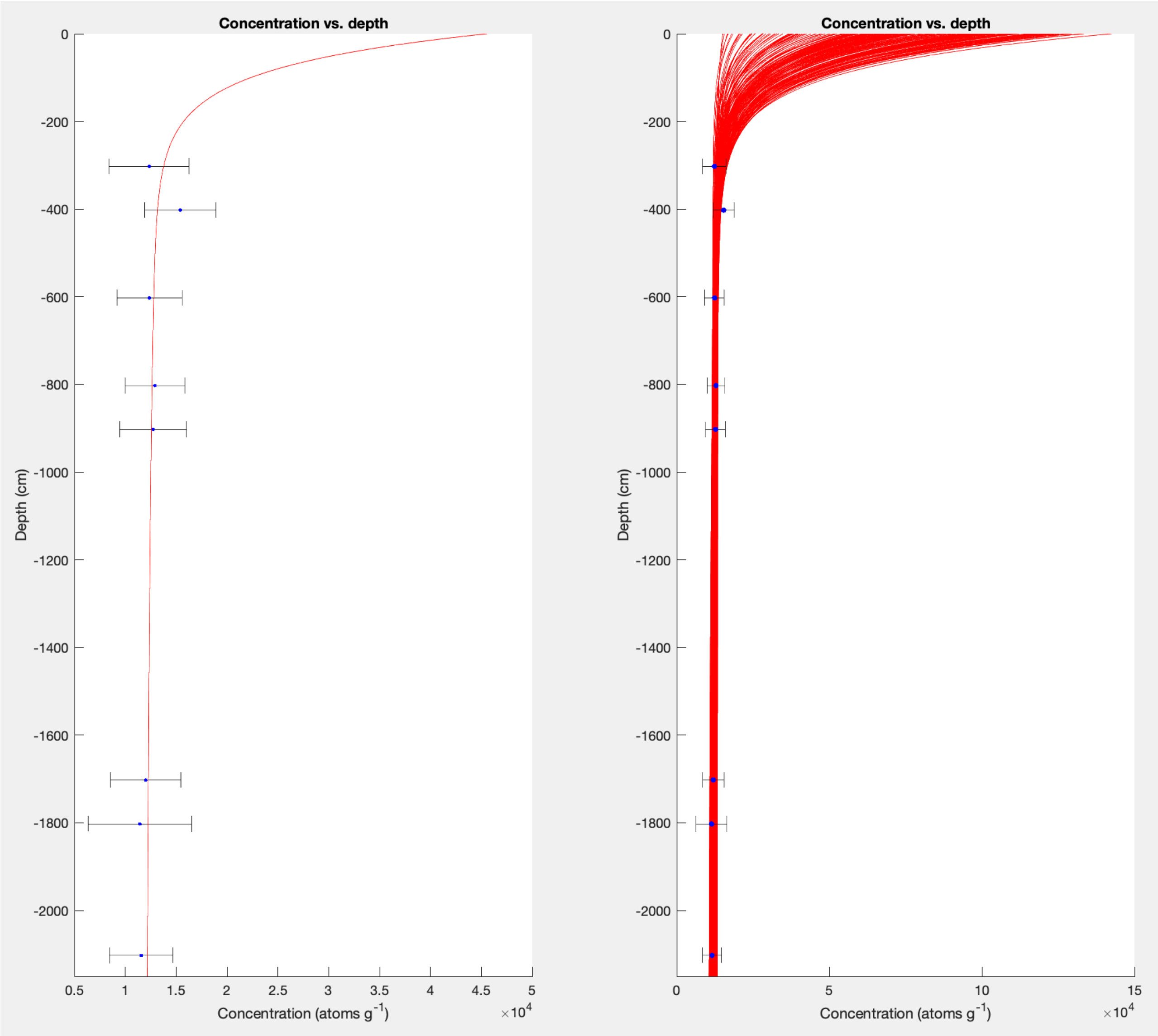

Fig. S3.4.1. Composite "*concentration vs. depth*" profile through ^{10}Be concentrations based on the measured concentrations for all samples. The dot plots show the median (dot), and the standard deviation (whiskers), n = 100.000.

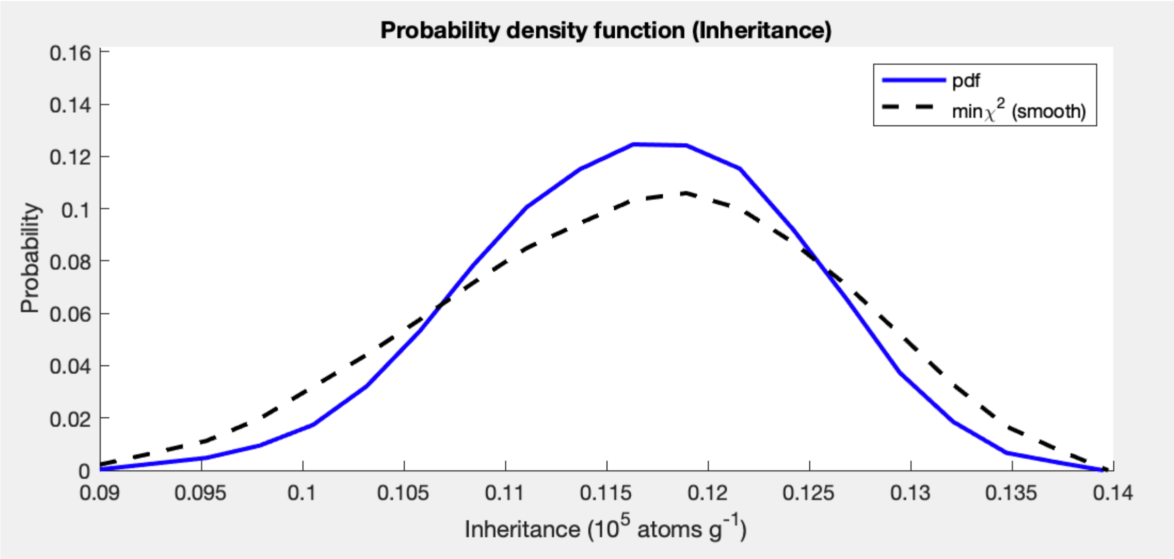

Fig. S3.4.2 Probability distribution function for the inheritance of ^{10}Be based on all measurements.

Table S3.4.2 Summary of output from the depth profile analysis on all ^{10}Be samples, n = 100.000.

Parameter	Exposure age (ka)	^{10}Be inheritance (10^4 atoms/g)
mean	14.6	1.15
median	14.0	1.15
mode	14.0	1.11
min chi^2	8.9	1.19
maximum	39.8	1.35
minimum	0	0.95
Bayesian most probable	10.5	1.19
Bayesian 2-sigma upper	26.7	1.28
Bayesian 2-sigma lower	0.7	1.02

The ^{26}Al depth profile (Fig. S3.4.3) for all eight samples using measured (unadjusted) concentrations reveals that there is a possibility that the sediments were exposed since the last glacial maximum

(Table S3.4.3), with a most probable exposure age of 16.0 (+26.3,-16.0) ka based on n=100,000 stochastically generated profiles and a 4σ fit to all data except KK06E which could not be fit, and allowing any erosion rate between 0 and 3 cm/ka. That exposure age is similar to the exposure age (14.6 ka) from the ^{10}Be depth profile and is consistent with the observation that Independence Fjord was occupied by LGM ice until sometime before early Holocene sediments. The most probable inheritance ^{26}Al concentration (Figure S3.4.4), i.e., prior to any post-glacial exposure, is 1.53 x 10^4 atoms/g (Table S3.4.3).

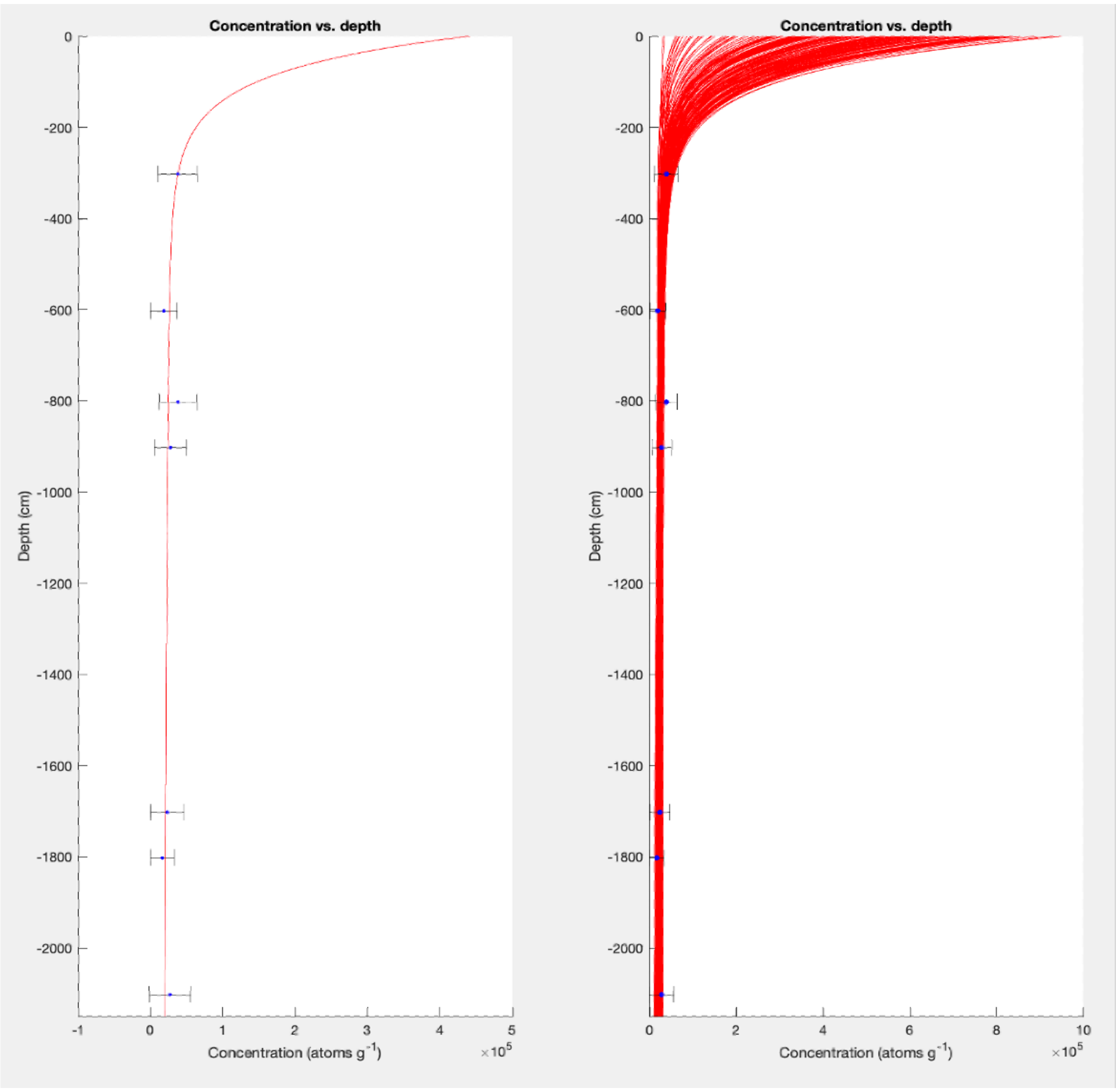

Figure S3.4.3. Composite "*concentration vs. depth*" profile through ^{26}Al concentrations based on all measurements (no adjustments) except KK06E which prevented a solution with a reasonable confidence (4σ)

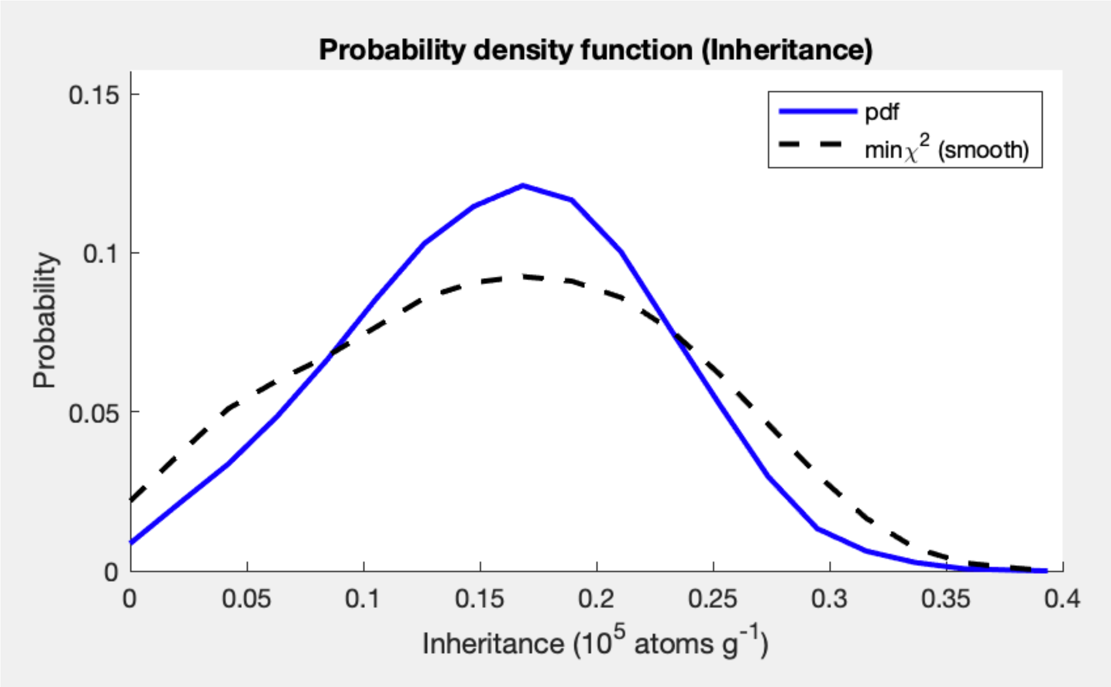

Figure S3.4.4 Probability distribution function for the inheritance of ^{26}Al based on all measurements except KK06E.

Table S3.4.3 Summary of output from the depth profile analysis on all ^{26}Al samples

Parameter	Exposure age (ka)	^{26}Al inheritance (10^4 atoms/g)
mean	16.0	1.53
median	15.1	1.53
mode	8.0	1.47
min chi^2	11.7	1.70
maximum	42.3	3.07
minimum	0.0	0.0
Bayesian most probable	9.5	1.89

Bayesian 2-sigma upper	31.2	2.52
Bayesian 2-sigma lower	0.3	0.31

Assuming that the composite depth profile is meaningful, then it is possible to calculate a mean burial age for the Kap København Formation. We can use the most probable inheritance concentration for ^{10}Be and ^{26}Al from the depth profile analyses above (Tables S3.4.2 and S3.4.3), instead of individual burial ages from each sample (Tables S3.3.1 and S3.3.2 and Fig. 3.3.1). The data used for the inheritance-based burial age is summarised in Table S3.4.4.

Table S3.4.4 Data used to calculate the burial age of a inheritance-based average concentration for the Kap København Formation

Sample ID	depth	density	lat	long	elev	Be	delBe	Al	delAl
	(cm)	(g/cc)	(dec)	(dec)	(m)	(atoms/g)	(atoms/g)	(atoms/g)	(atoms/g)
dp_ave	2100	1.9	82.4961	-21.231	97	11500	600	15300	3060

The result is an 'average' burial age of 3.36 (+0.59/-0.33) Ma, which again must be interpreted as a maximum burial duration. As discussed above, KK06E contains a relatively high concentration of ^{10}Be and ^{26}Al relative to all other samples and has a $^{26}Al/^{10}Be$ ratio that is relatively low, which is consistent with that site being exposed for longer than other sites since the emergence of the Kap København Formation. It was not included in the depth profile analysis to fit a most probable solution to a composite concentration-depth profile. However, its high concentration is consistent with the depth profile analysis indicating a post-emergence exposure of around 15 ka for both ^{10}Be and ^{26}Al (*c.f.* Table S3.4.2 and Table S3.4.3).

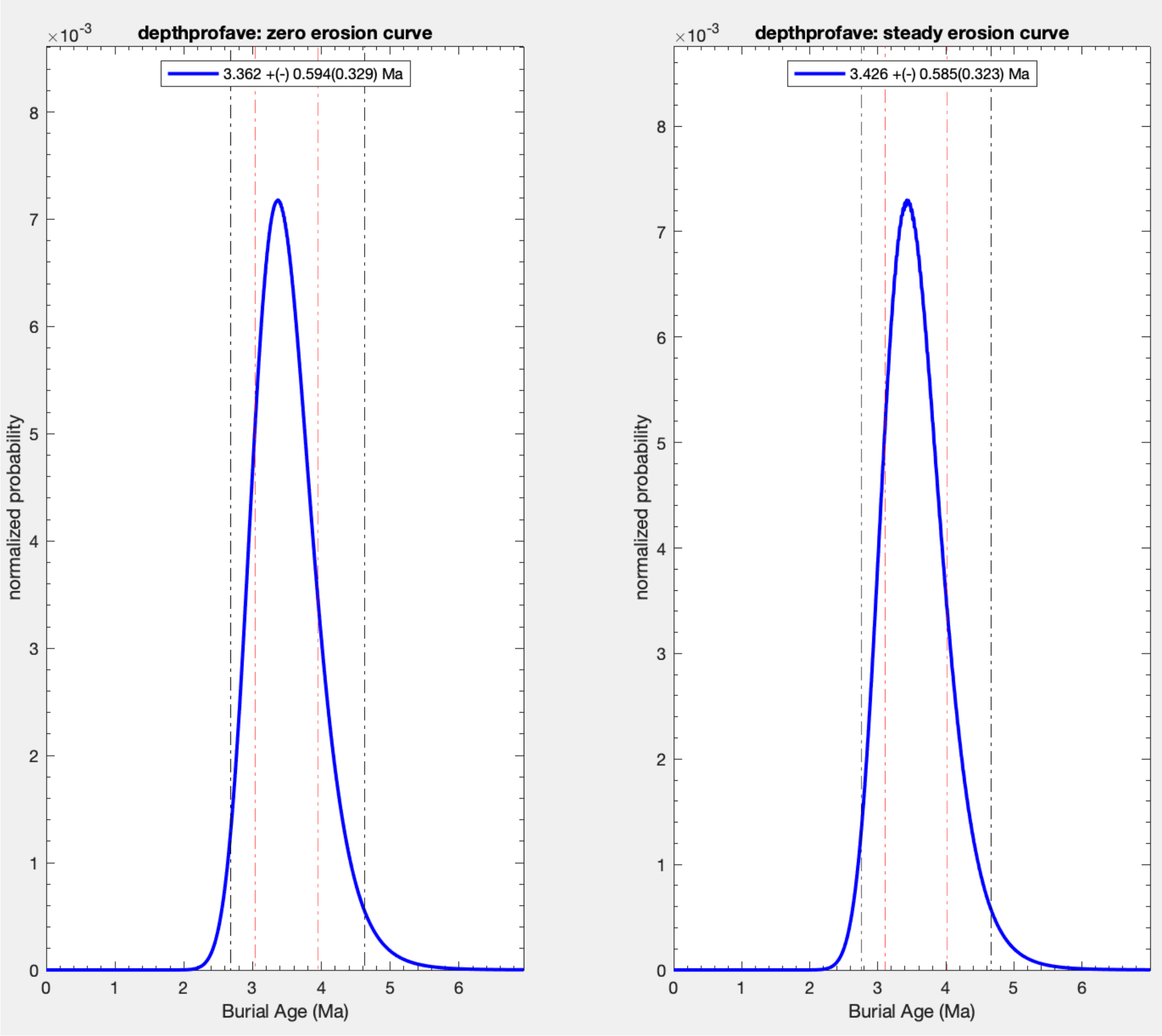

Fig. S3.4.5 The probability distributions for the burial age based on the inherited concentrations of ^{10}Be and ^{26}Al from the depth profile analysis. The steady state erosion pdf considers that there may have been some erosion during burial (we use the maximum erosion permitted by the concentrations) to test the sensitivity of the data to erosion.

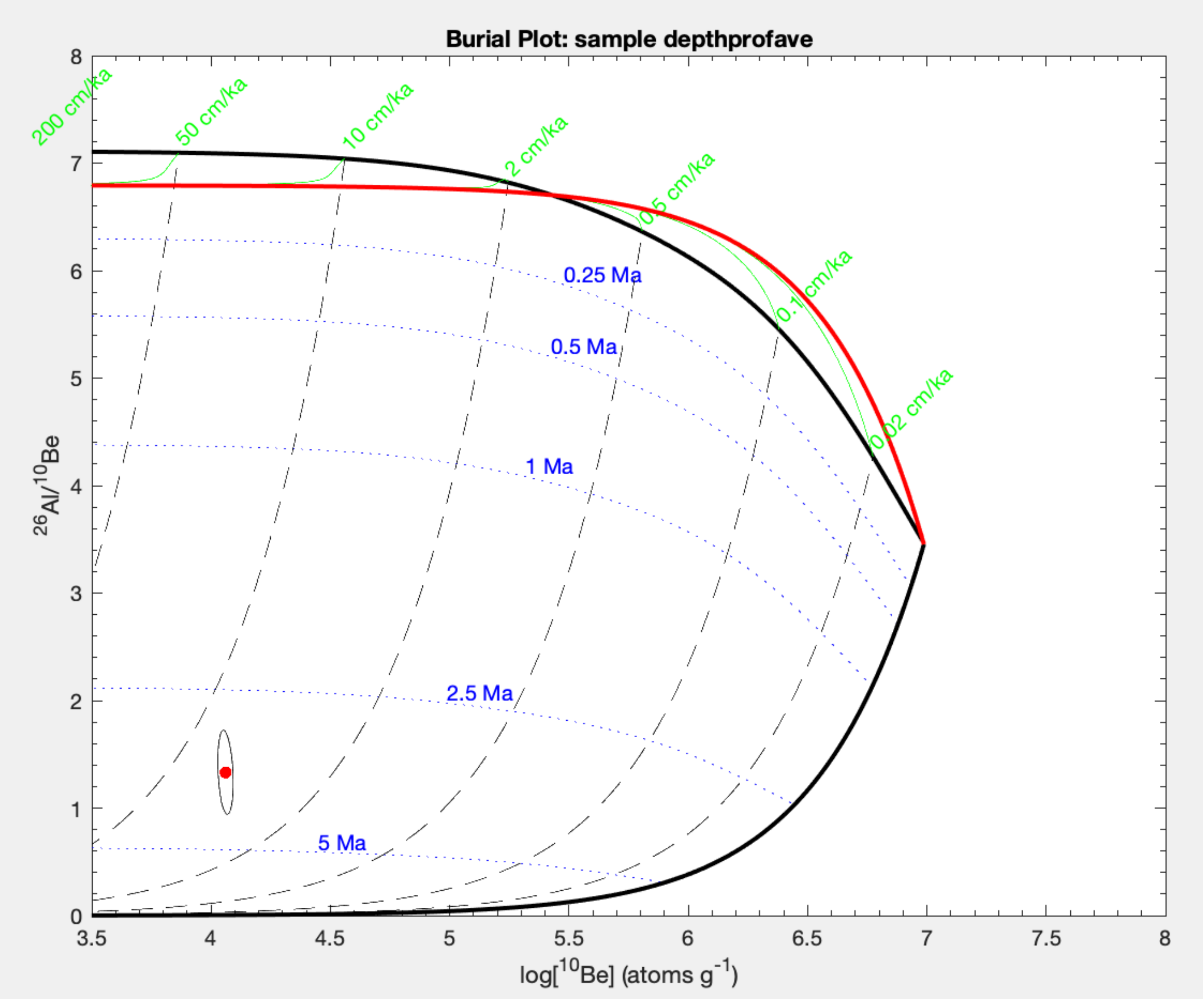

Fig. S3.4.6 A $^{26}Al/^{10}Be$ vs. ^{10}Be graph showing the position of the inheritance concentrations and ratio.

3.5 ^{26}Al vs ^{10}Be isochron

For comparison purposes, the samples are plotted on a $^{26}Al/^{10}Be$ isochron[139]. A benefit of an isochron burial age approach is that if multiple burial histories have occurred in the past, they will not affect the isochron burial age. No cobbles were available for the *cobble isochron method*, and no paleosols were present to indicate a paleo surface had been exposed for 10^3 to 10^4 years prior to burial to build up sufficient concentration for a *depth profile isochron method*. Nevertheless, assuming that all eight samples spread over different sites with different incision histories are dating the same burial event,

we obtain a depth profile isochron curve that has a mean slope of 14.5 (impossible for a simple continuous burial) and mean age that is negative (Fig. S3.5.1). This result is consistent with a complex burial-exposure history in which some of the samples but possibly not all have been exposed for different durations during or at the end of the burial history, as was concluded from the depth profile analysis above.

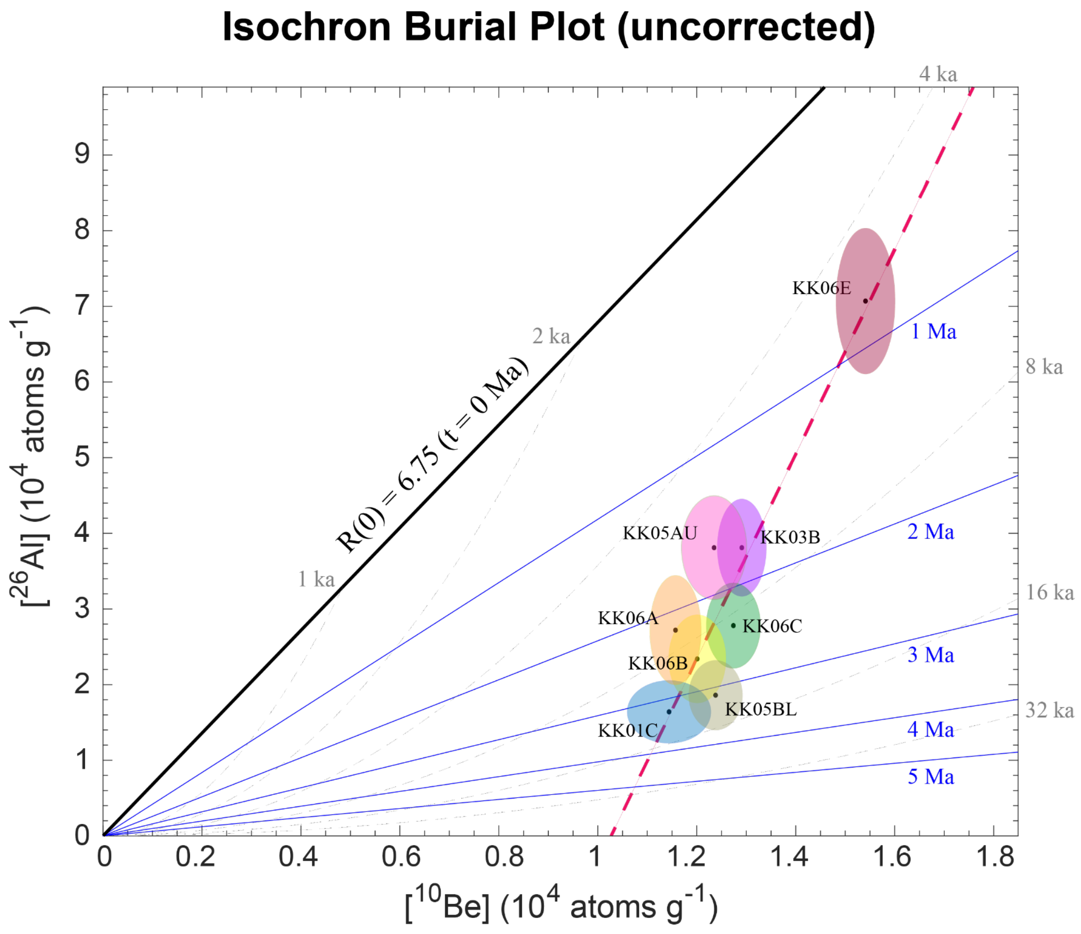

Fig. S3.5.1. ^{26}Al vs. ^{10}Be isochron plot using all eight KK samples.

3.6 Favoured interpretation of the burial age of the Kap København Formation

Using the concentrations of ^{10}Be and ^{26}Al in sand collected below four different terraces cut into the Kap København Formation, the Bayesian most probable burial duration is determined to be between 2.41 ± 0.24(0.16) Ma and 2.55 ± 0.24(0.16) Ma (1σ unc) for a production ratio of 6.75 and 7.42 respectively (Fig. S3.3.1), based on the convolution of probability distribution functions for all eight samples and including maximum steady state erosion. As the individual probability distribution functions are maxima, then this would be interpreted as an average of the maxima, and therefore a maximum age for the Kap København Formation.

If we calculate the most probable inheritance in a composite depth profile from all sample sites and use the inheritance of ^{10}Be and ^{26}Al as an average concentration without the impact of post-emergence exposure, we obtain a maximum limiting age of 3.36 (+0.59/-0.33) Ma.

However, we consider that a large scatter in maximum burial ages exist among different locations, and that at least some of those samples a post-emergence exposure has affected their ^{26}Al/^{10}Be ratios by an unknown amount. Therefore, we have calculated the most probable burial duration from only the three deepest samples (KK06A, B, & C; all in the longest depth profile) to minimise the effects of post-emergence exposure (Table S3.6.1). The convolved probability distribution functions are presented in Fig S3.6.1 and S3.6.2 for a ^{26}Al/^{10}Be production ratio of 6.75 and 7.42 respectively.

Table S3.6.1. Data selected from the deepest three samples of KK06 for our best estimate of the burial age of the Kap København Formation.

**Sample ID**	**depth**	**density**	**lat**	**long**	**elev**	**Be**	**delBe**	**Al**	**delAl**
	**(cm)**	**(g/cc)**	**(dec)**	**(dec)**	**(m)**	**(atoms/g)**	**(atoms/g)**	**(atoms/g)**	**(atoms/g)**
KK06C	900	1.9	82.4961	-21.2310	97	12741	544	27780	5604
KK06B	1700	1.9	82.4961	-21.2310	97	12013	574	23431	5821
KK06A	2100	1.9	82.4961	-21.2310	97	11569	516	27187	7207

The most probable age for those samples assuming either no erosion or the maximum possible erosion in the catchment for build-up of the ^{10}Be and ^{26}Al is, respectively, 2.49 (+0.46/-0.25) or 2.57 (+0.45/-0.25) Ma for a production ratio of 6.75, or 2.68 (+0.46/-0.26) or 2.71 (+0.45/-0.25) Ma for a production ratio of 7.42. As these are probably the most reliable three samples, and because their error weightings are similar, we take the lowest and the highest 1σ ages, which yields a range of 2.24

to 3.16 Ma, and a midpoint age of 2.70 +0.46/-0.46 Ma as the maximum burial age of the Kap København Formation. We also note that the position of these three samples on the ^{26}Al vs. ^{10}Be isochron plot is consistent with this midpoint range.

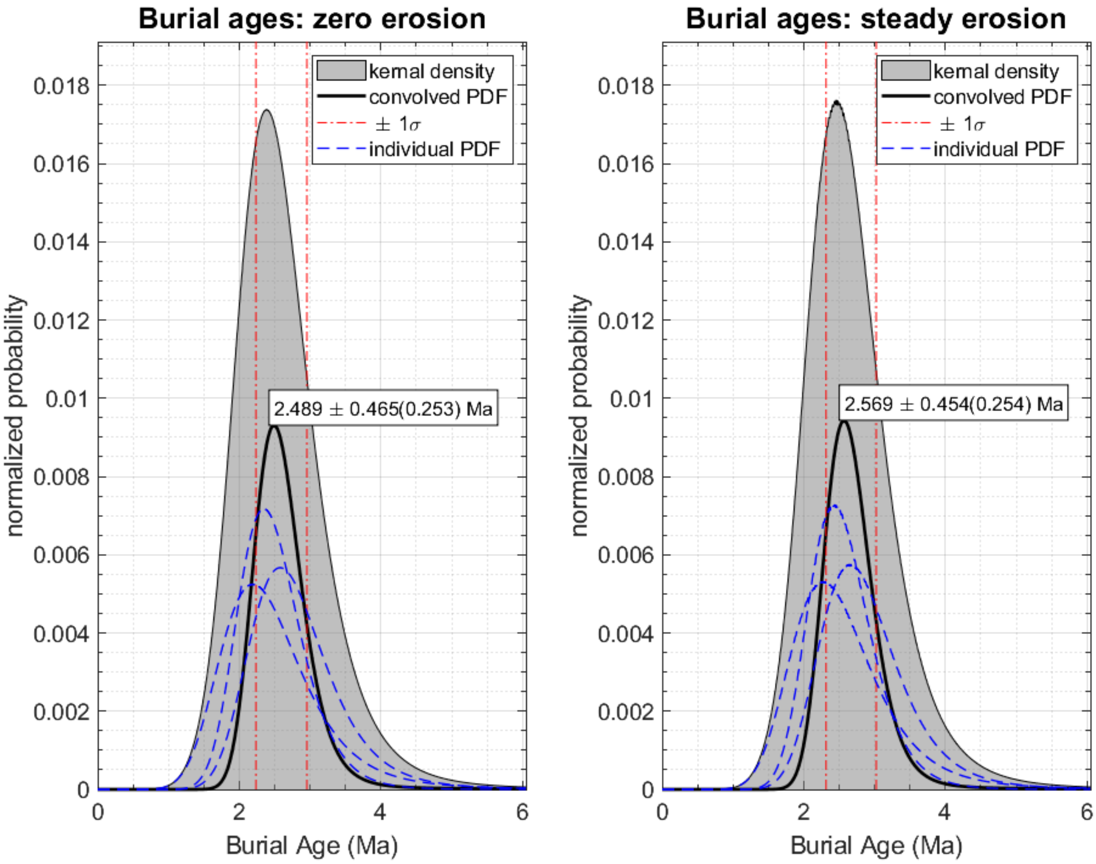

Fig. S3.6.1 Convolved probability distribution function most probable burial age of the three deepest samples from a single depth profile, KK06A, B, & C for a $^{26}Al/^{10}Be$ production ratio of 6.75. Figure on the left assumes zero erosion and figure on the right assumes the maximum steady state erosion in the catchment. The thick curve represents the convolved probability distribution function (multiplication of the probabilities of the three samples). The red dashed lines represent the 1σ uncertainty for the most probable age. The blue dashed lines are the probability distributions for the individual samples. The shaded area represents the kernel density (the sum of the individual probabilities density functions) and is only shown as it is commonly used by others to represent the average age of a dataset.

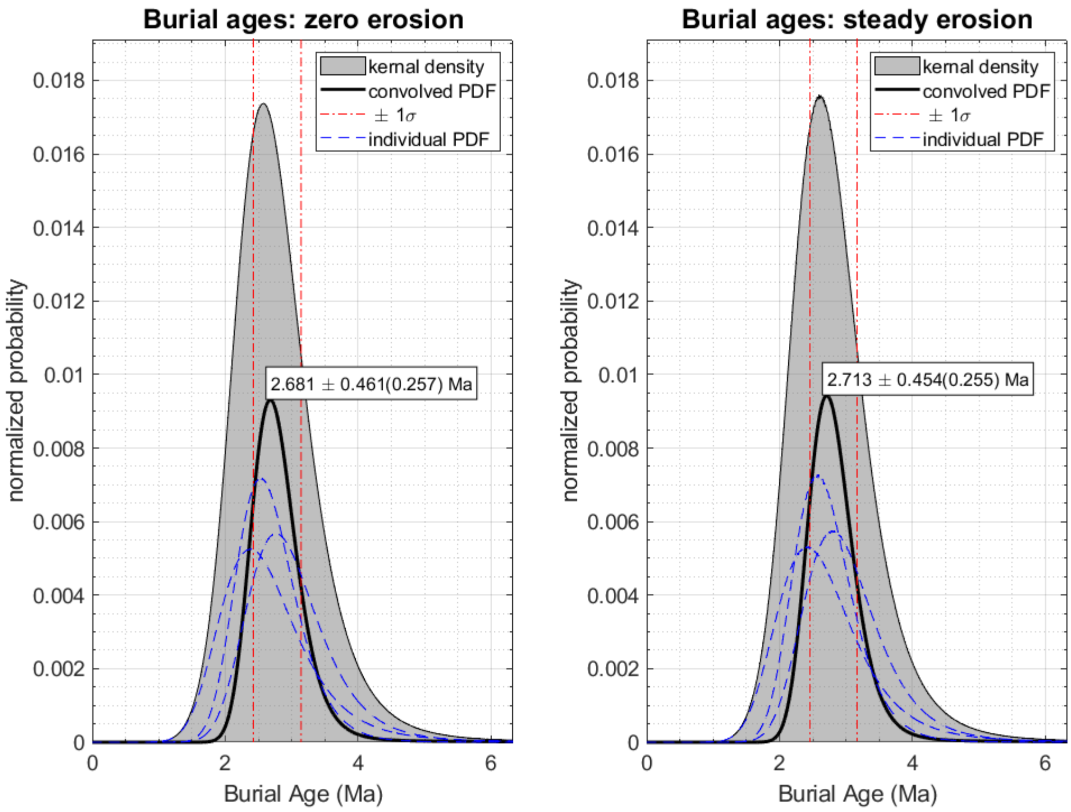

Figure S3.6.2 Same as Fig. S3.6.1 except for a $^{26}Al/^{10}Be$ production ratio of 7.42.

4. Mineralogy

4.1 Clay mineral characterization

We separated the fraction <2 µm from ~1 g of the bulk samples by centrifugation. Plant material was hand-picked, and the rest of organic compounds digested overnight with H_2O_2 (30% w/w, Sigma-Aldrich) at 60 °C. The remaining sample was then washed with 100 ml doubly deionised water (Millipore, resistivity ≥18.2 MΩcm) and we added a pinch of $Na_2H_2P_2O_7$ (practical grade, Sigma Aldrich) as a dispersant. The suspension was then mixed on a rotary shaker at 300 rpm overnight and the fraction <2 µm was separated the next day. We then analysed the clay fraction as air-dried (AD), treated in a chamber of ethylene glycol vapours (80 °C) for at least 8 h (EG sample) and heated at 400 °C for 1 h (400 °C sample). The instrumental parameters for the analysis of oriented clay mounts were the same as for the bulk powder samples, except that the diffractograms were collected on an

unspun sample from 2 - 60 °2θ with the step size of 0.6°, the step time of 3 sec and the opening of the divergence slit of 0.6°.

4.2 Mineralogic composition

Mineralogic composition in weight percent calculated based on crystalline material (Table S4.2.1) and including amorphous (glass and organic) content (Table S4.2.2). The composition is distinct between the four stratigraphic units and the averages and standard deviations are presented in Table S4.2.3 and 4. The model minerals chosen to represent the minerals in the deposit are noted in Table S4.2.3 and 4. Table S4.2.6. shows minerals used as models in adsorption studies and their pre-treatments and purification procedure. Minerals with an average of 0.6 wt% or more were included in the adsorption study.

Table S4.2.1. Tables of mineralogic composition of samples including the corresponding stratigraphic units. Excl. amorphous content.

EXCL AMORPH																				
XRD		Quartz		K-feldspar	Plagioclas	Hornblenc	Diopside	Rutile	Magnetite	Halite	Pyrite	Gypsum	**TOTAL**	Dioctahed	Trioctahec	Trioctahec	Trioctahec	Talc	**TOTAL**	SUM
Adsorption study		Quartz		Orthoclas	Orthoclas	Tremolite	Diopside						**NON-CLAY**	Illite	Biotite	Chlorite	Smectite	Talc	**CLAYS**	
**12-01-31**	B2	55.2		15.5	9.6	1.0	5.4	0.5	0.4	0.5	-	-	87.9	5.8	0.6	3.4	1.6	0.7	12.2	100.1
**12-01-32**	B2	56.2		15.8	9.6	0.8	4.8	0.5	0.3	0.4	-	-	88.5	5.5	0.1	2.8	2.8	0.5	11.8	100.3
12-01-33	B2	56.6		14.6	9.8	1.2	4.3	0.5	0.2	0.4	-	-	87.6	4.9	0.7	2.4	3.8	0.4	12.2	99.8
12-01-34	B2	64.3		14.3	7.9	1.0	4.4	0.5	0.4	0.1	-	-	92.9	4.1	-	1.2	1.2	0.8	7.3	100.2
12-01-35	B2	63.3		13.7	8.9	0.2	5.3	0.5	0.4	-	-	-	92.3	4.5	-	1.3	1.3	0.5	7.6	99.9
12-01-36	B2	65.3		13.1	9.0	0.8	4.0	0.3	0.2	-	-	-	92.7	3.7	-	1.1	1.6	1.0	7.4	100.1
12-01-37	B2	67.9		12.8	8.3	0.8	3.7	0.3	0.2	-	-	-	94.0	3.6	0.2	0.8	1.1	0.4	6.1	100.1
12-01-38	B2	67.9		12.8	7.7	0.7	3.6	0.3	0.3	-	-	-	93.3	3.2	-	0.9	2.4	0.2	6.7	100.0
12-01-39	B2	68.9		12.2	7.8	0.6	3.6	0.2	0.3	-	-	-	93.6	3.2	0.3	0.8	1.0	1.1	6.4	100.0
12-01-41	B3	76.5		6.8	7.2	0.4	3.6	-	-	-	-	-	94.5	2.2	0.8	0.7	0.8	1.0	5.5	100.0
12-01-42	B3	75.7		7.1	7.1	0.9	3.6	0.2	-	-	-	-	94.5	2.2	0.2	0.8	1.7	0.7	5.6	100.1
12-01-43	B3	79.1		6.5	6.6	0.5	3.0	-	-	-	-	-	95.7	2.2	-	0.4	0.9	0.8	4.3	100.0
12-01-44	B3	73.0		8.2	7.2	0.8	4.4	-	0.2	-	-	-	93.8	2.4	0.4	1.0	1.8	0.7	6.3	100.1
12-01-45	B3	74.0		6.8	7.9	0.8	3.7	0.2	-	-	-	-	93.4	2.5	0.5	0.8	1.5	1.4	6.7	100.1
12-01-46	B3	73.6		6.8	8.0	1.1	4.4	0.1	0.2	-	-	-	94.2	1.8	0.4	0.8	1.6	1.2	5.8	100.0
12-01-47	B3	77.0		6.5	7.3	0.7	3.2	-	-	-	-	-	94.7	1.8	0.5	0.7	1.0	1.3	5.2	99.9
12-01-48	B3	69.5		7.9	9.0	1.5	5.3	0.2	-	-	-	-	93.4	2.3	0.4	1.0	1.6	1.3	6.6	100.0
12-01-49	B3	75.2		7.8	7.8	0.6	3.0	0.2	-	-	-	-	94.6	2.4	0.7	0.7	0.7	1.1	5.6	100.2
12-01-50	B3	76.0		6.9	7.6	0.9	3.3	0.1	-	-	-	-	94.8	2.7	0.2	0.8	1.1	0.2	5.0	99.8
12-01-51	B3	74.4		8.1	8.7	0.7	2.5	-	0.2	0.1	-	-	94.7	2.0	0.2	1.0	0.8	1.3	5.3	100.0
12-01-52	B3	75.5		7.3	7.7	1.0	4.0	-	-	-	-	-	95.5	1.9	0.5	0.7	0.6	0.9	4.6	100.1
199A	B3 - 50	64.0		13.9	8.0	0.3	4.0	-	0.2	-	-	-	90.4	4.3	0.7	1.1	1.9	1.5	9.5	99.9
199B	B3 - 50	60.2		14.5	10.2	0.4	5.4	-	-	-	-	-	90.7	4.0	0.5	2.0	2.4	0.4	9.3	100.0
200A	B3 - 50	59.7		15.0	9.2	0.5	3.4	0.6	-	0.2	-	0.6	89.1	5.9	1.0	2.2	0.8	0.9	10.8	99.9
200B	B3 - 50	57.8		15.2	10.5	0.2	4.6	0.6	-	-	-	0.8	89.7	5.4	0.9	2.4	0.9	0.6	10.2	99.9
203A	B1 - 75	71.4		8.5	7.7	1.1	3.5	-	-	-	0.6	0.5	93.3	1.7	0.6	0.4	3.3	0.9	6.9	100.2
203B	B1 - 75	71.5		8.7	7.6	1.1	2.9	-	-	0.1	0.6	0.5	93.0	1.7	0.4	0.4	4.1	0.5	7.1	100.1

Table S4.2.2. Tables of mineralogic composition of samples including the corresponding stratigraphic units. Incl. amorphous content.

Individual XRD patterns (observed intensity), refinements (calculated intensity) “Source Data S4” and their difference can be downloaded as tiff files (“Source Data S5”).

INCL. AMORPH																				
XRD		Quartz	Plant&glas	K-feldspar	Plagioclas	Hornblenc	Diopside	Rutile	Magnetite	Halite	Pyrite	Gypsum	**TOTAL**	Dioctahed	Trioctahec	Trioctahec	Trioctahec	Talc	**TOTAL**	SUM
Adsorption study		Quartz		Orthoclas	Orthoclas	Tremolite	Diopside						**NON-CLAY**	Illite	Biotite	Chlorite	Smectite	Talc	**CLAYS**	
**12-01-31**	B2	45.3	17.9	12.7	7.9	0.8	4.4	0.4	0.3	0.4	-	-	90.1	4.8	0.5	2.8	1.3	0.6	10.0	100.1
**12-01-32**	B2	42.0	25.3	11.8	7.2	0.6	3.6	0.4	0.2	0.3	-	-	91.4	4.1	0.1	2.1	2.1	0.4	8.8	100.3
12-01-33	B2	47.3	16.4	12.2	8.2	1.0	3.6	0.4	0.2	0.3	-	-	89.6	4.1	0.6	2.0	3.2	0.3	10.2	99.8
12-01-34	B2	59.0	8.3	13.1	7.2	0.9	4.0	0.5	0.4	0.1	-	-	93.5	3.8	-	1.1	1.1	0.7	6.7	100.2
12-01-35	B2	63.3	-	13.7	8.9	0.2	5.3	0.5	0.4	-	-	-	92.3	4.5	-	1.3	1.3	0.5	7.6	99.9
12-01-36	B2	65.3	-	13.1	9.0	0.8	4.0	0.3	0.2	-	-	-	92.7	3.7	-	1.1	1.6	1.0	7.4	100.1
12-01-37	B2	67.9	-	12.8	8.3	0.8	3.7	0.3	0.2	-	-	-	94.0	3.6	0.2	0.8	1.1	0.4	6.1	100.1
12-01-38	B2	67.9	-	12.8	7.7	0.7	3.6	0.3	0.3	-	-	-	93.3	3.2	-	0.9	2.4	0.2	6.7	100.0
12-01-39	B2	68.9	-	12.2	7.8	0.6	3.6	0.2	0.3	-	-	-	93.6	3.2	0.3	0.8	1.0	1.1	6.4	100.0
12-01-41	B3	76.5	-	6.8	7.2	0.4	3.6	-	-	-	-	-	94.5	2.2	0.8	0.7	0.8	1.0	5.5	100.0
12-01-42	B3	75.7	-	7.1	7.1	0.9	3.6	0.2	-	-	-	-	94.5	2.2	0.2	0.8	1.7	0.7	5.6	100.1
12-01-43	B3	79.1	-	6.5	6.6	0.5	3.0	-	-	-	-	-	95.7	2.2	-	0.4	0.9	0.8	4.3	100.0
12-01-44	B3	73.0	-	8.2	7.2	0.8	4.4	-	0.2	-	-	-	93.8	2.4	0.4	1.0	1.8	0.7	6.3	100.1
12-01-45	B3	74.0	-	6.8	7.9	0.8	3.7	0.2	-	-	-	-	93.4	2.5	0.5	0.8	1.5	1.4	6.7	100.1
12-01-46	B3	73.6	-	6.8	8.0	1.1	4.4	0.1	0.2	-	-	-	94.2	1.8	0.4	0.8	1.6	1.2	5.8	100.0
12-01-47	B3	77.0	-	6.5	7.3	0.7	3.2	-	-	-	-	-	94.7	1.8	0.5	0.7	1.0	1.3	5.2	99.9
12-01-48	B3	69.5	-	7.9	9.0	1.5	5.3	0.2	-	-	-	-	93.4	2.3	0.4	1.0	1.6	1.3	6.6	100.0
12-01-49	B3	75.2	-	7.8	7.8	0.6	3.0	0.2	-	-	-	-	94.6	2.4	0.7	0.7	0.7	1.1	5.6	100.2
12-01-50	B3	76.0	-	6.9	7.6	0.9	3.3	0.1	-	-	-	-	94.8	2.7	0.2	0.8	1.1	0.2	5.0	99.8
12-01-51	B3	74.4	-	8.1	8.7	0.7	2.5	-	0.2	0.1	-	-	94.7	2.0	0.2	1.0	0.8	1.3	5.3	100.0
12-01-52	B3	75.5	-	7.3	7.7	1.0	4.0	-	-	-	-	-	95.5	1.9	0.5	0.7	0.6	0.9	4.6	100.1
199A	B3 - 50	64.0	-	13.9	8.0	0.3	4.0	-	0.2	-	-	-	90.4	4.3	0.7	1.1	1.9	1.5	9.5	99.9
199B	B3 - 50	60.2	-	14.5	10.2	0.4	5.4	-	-	-	-	-	90.7	4.0	0.5	2.0	2.4	0.4	9.3	100.0
200A	B3 - 50	52.5	12.0	13.2	8.1	0.4	3.0	0.5	-	0.2	-	0.5	90.4	5.2	0.9	1.9	0.7	0.8	9.5	99.9
200B	B3 - 50	57.8	-	15.2	10.5	0.2	4.6	0.6	-	-	-	0.8	89.7	5.4	0.9	2.4	0.9	0.6	10.2	99.9
203A	B1 - 75	71.4	-	8.5	7.7	1.1	3.5	-	-	-	0.5	0.5	93.2	1.7	0.6	0.4	3.3	0.9	6.9	100.2
203B	B1 - 75	71.5	-	8.7	7.6	1.1	2.9	-	-	0.1	0.5	0.5	92.9	1.7	0.4	0.4	4.1	0.5	7.1	100.1

Table S4.2.3. Unit averages (avg.) and standard deviations (stddev) excluding amorphous material as identified with XRD. ()=number of samples, / = no data, standard deviation calculated when more than one sample occurrence. Minerals with an average of 0.6 wt% or more were included in the adsorption study. The corresponding model minerals used in adsorption experiments are listed.

**Unit**	**B2 (9)**		**B3 (12)**		**B3-50 (4)**		**B1 - 75 (2)**		**Equivalent applied in adsorption study**
**XRD id.**	Avg.	stddev	Avg.	stddev	Avg.	stddev	Avg.	stddev	
**Quartz**	62.8	5.1	75.0	2.3	60.4	2.3	71.5	0.0	Quartz
**K-feldspar**	13.9	1.2	7.2	0.6	14.7	0.5	8.6	0.1	Orthoclase
**Plagioclase**	8.7	0.8	7.7	0.6	9.5	1.0	7.7	0.1	Orthoclase

**Hornblende**	0.8	0.3	0.8	0.3	0.3	0.1	1.1	/	Tremolite
**Diopside**	4.3	0.7	3.7	0.7	4.4	0.7	3.2	0.3	Diopside
**Rutile**	0.4	0.1	0.2	/	0.6	/	/	/	/
**Magnetite**	0.3	0.1	0.2	/	0.2	/	/	/	/
**Halite**	0.3	0.1	0.1	/	0.2	/	0.1	/	/
**Pyrite**	/	/	/	/	/	/	0.6	/	/
**Gypsum**	/	/	/	/	0.7	0.1	0.5	/	/
**TOTAL non-clays**	91.4	2.5	94.5	0.7	90.0	0.6	93.2	0.2	
**Dioctahedral mica**	4.3	0.9	2.2	0.3	4.9	0.8	1.7		Illite
**Trioctahedral mica**	0.4	0.2	0.4	0.2	0.8	0.2	0.5	0.1	Illite
**Trioctahedral chlorite**	1.6	0.9	0.8	0.2	1.9	0.5	0.4		Chlorite
**Trioctahedral smectite**	1.9	0.9	1.2	0.4	1.5	0.7	3.7	0.4	Smectite
**Talc**	0.6	0.3	1.0	0.3	0.9	0.4	0.7	0.2	Talc
**TOTAL clays**	8.6	2.5	5.5	0.7	9.9	0.6	7.0	0.1	/

Table S4.2.4. Unit averages (avg.) and standard deviations (stddev) including amorphous material as identified with XRD. ()=number of samples, / = no data, standard deviation calculated when more than one sample occurrence. Minerals with an average of 0.6 wt% or

more were included in the adsorption study. The corresponding model minerals used in adsorption experiments are listed.

Unit	B2 (9)		B3 (12)		B3-50 (4)		B1 - 75 (2)		Equivalent applied in adsorption study
XRD id.	Avg.	stddev	Avg.	stddev	Avg.	stddev	Avg	stddev	
Quartz	60.2	9.5	75.0	2.3	58.6	2.3	71.5	0.0	Quartz
Plant & glass	16.7	6.9	/	/	12.0	0.5	8.6	0.1	/
K-feldspar	12.7	0.6	7.2	0.6	14.2	1.0	7.7	0.1	Orthoclase
Plagioclase	8.0	0.6	7.7	0.6	9.2	0.1	1.1	/	Orthoclase
Hornblende	0.7	0.2	0.8	0.3	0.3	0.7	3.2	0.3	Tremolite
Diopside	3.9	0.5	3.7	0.7	4.3	/	/	/	Diopside
Rutile	0.4	0.1	0.2	0.0	0.6	/	/	/	/
Magnetite	0.3	0.1	0.2	0.0	0.2	/	0.1	/	/
Halite	0.2	0.1	0.1	/	0.2	/	0.6	/	/
Pyrite	/	/	/	/	/	0.1	0.5	/	/
Gypsum	/	/	/	/	0.7	0.6	93.2	0.2	/
TOTAL non-clays	92.6	1.4	94.5	0.7	90.3	0.8	1.7		Illite
Dioctahedral mica	3.8	0.4	2.2	0.3	4.7	0.2	0.5	0.1	Illite
Trioctahedral mica	0.3	0.2	0.4	0.2	0.8	0.5	0.4		Chlorite
Trioctahedral chlorite	1.3	0.5	0.8	0.2	1.9	0.7	3.7	0.4	Smectite
Trioctahedral smectite	1.7	0.7	1.2	0.4	1.5	0.4	0.7	0.2	Talc

Talc	0.6	0.3	1.0	0.3	0.8	0.6	7.0	0.1	/

Table S4.2.5. Mineralogic composition (XRD data) of 9 Triassic mudstones from the Kim Fjelde area. Reference (ref. [140]) is available upon request to the Geological Survey of Denmark and Greenland.

Mineral	# 230	# 231	# 232	# 233	# 234	# 235	# 236	# 237	# 238
Quartz	49	49	51	57	48	46	45	46	42
Microcline	4	4	5	2	2	2	4	5	5
Albite	7	6	6	5	8	9	11	16	15
Mica	1	1	1	1	1	1	1	1	1
Ilite	19	18	18	8	13	14	20	18	21
Chlorite	8	8	10	5	6	6	10	10	11
Kaolinite	2	2	2	1	1	2	1	1	1
Calcite	4	5	4	14	15	12	2	0	0
Dolomite	4	4	3	6	5	5	2	1	1
Pyrite	3	3	2	0.5	2	2	3	2	2
Apatite	0	0	0	0	0	0	0	0	0
sum	101	100	102	99.5	101	99	99	100	99

Table S4.2.6. Pure and purified minerals used in the adsorption studies.

Mineral	Used as received	Only <2 µm fraction used	Heated	Boiled in 1 M NaOH	Washed in 2 M NaOH	Impurities	Source
Quartz	Crushed to reduce particle size	/	/	/	/	/	unknow n

Diopside (pyroxene)	/	<20 µm removed prior to heating	900 °C for 2 h	/	yes	Minor hornblende and quartz	Krantz-Minerals, Germany
Tremolite (amphibole)	/	/	850 °C for 2 h	yes	/	Minor hornblende	unknown
Orthoclase (K-feldspar)	/	/	800 °C for 2 h	yes	/	Minor plagioclase and quartz	unknown
Illite (IMt-1)	/	yes	/	/	/	Minor quartz	Source Clay Repository
Clinochlore (chlorite, CCa2)	Crushed to reduce particle size	/	/	/	/	/	Source Clay Repository
Talc	Crushed to reduce particle size	/	/	/	/	/	Isua, vein in dunite
Montmorillonite (smectite, SWy-2)	/	yes	/	/	/	Minor quartz	Source Clay Repository

4.3 Adsorption

4.3.1 Adsorption isotherms

Adsorption of DNA to K-feldspar, quartz, tremolite (amphibole), diopside (pyroxene), talc and illite is best fit to a Langmuir isotherm suggesting a monolayer adsorption process. Adsorption of DNA to smectite and chlorite is best described with a Freundlich isotherm suggesting a multilayer adsorption process meaning that, in theory, an indefinite amount of DNA can be adsorbed. The fit parameters for all isotherms are provided in Table S4.3.1.1 The relatively high standard deviation of the extraction recovery for the non-clay minerals is likely caused by the low adsorption capacities of DNA leading to the extraction yield being close to the detection limit of the Biophotometer.

Table S4.3.1.1. Adsorption isotherm parameters and extraction yield.

**Model**	**Langmuir isotherm**			**Extraction**
	q_{max}	K_L	R^2	*Recovery (%)*
K-feldspar	0.26	0.0072	0.979	43 ± 28 (3)
Quartz	0.46	0.0064	0.993	43 ± 32 (7)
Amphibole	0.60	0.0100	0.969	43 ± 17 (3)
Diopside	0.73	0.0230	0.878	36 ± 10 (3)
Talc	15.48	0.0400	0.987	10 ± 2 (4)
Illite	16.47	0.0050	0.991	10 ± 2 (4)
**Model**	**Freundlich isotherm**			**Extraction**
	K_F	*1/n*	R^2	*Recovery (%)*

	0.73	0.43	0.992	
Chlorite				10 ± 1 (3)
	0.40	0.97	0.991	
Smectite				4 ± 1 (4)

Langmuir isotherm: Adsorption capacity = (q_{max} * K_L * equilibrium concentration) / (1 + K_L * equilibrium concentration), ()= number of repetitions, q_{max} = the maximum adsorption capacity , K_L= Langmuir constant, R^2 = coefficient of determination, Freundlich isotherm: Adsorption capacity = K_F * equilibrium capacity$^{(1/n)}$, K_F=Freundlich constant, n= Freundlich exponent.

4.4 Thermal age

Thermal age is a measure which enables simple comparison between ancient biomolecular targets by normalising them to an equivalent (thermal) age, allowing all samples to be treated as having experienced a constant temperature of 10°C. Thus, samples from cooler sites, which experience slower rates of chemical reaction, will have thermal ages younger than their geochronological age, whilst samples from warmer sites will be thermally 'older'. Various factors can affect the effective diagenetic temperature experienced by a sample (and therefore impact on its thermal age), from burial depth to seasonal and interglacial / glacial cycles[141–143]. Using a simple DNA half-life estimate for bone[23], projected to a long term stable -17C temperature (current MAT for Kap København[7], Table S4.4.1) provides a theoretical average fragment length of 51bp at 1.93 Ma, (50 bp at 2Ma) which is comparable to what we observe in our sequencing data (Table S4.4.2). However, when accounting for more complex models involving periods of non-frozen conditions for hundreds of thousands of years (Table S4.4.2), the estimated DNA degradation is more severe than we can observe in our data and the DNA molecules are degraded to 1-2 bp. Assuming that such periods of non-frozen conditions represent a more realistic scenario, it suggests that DNA in sediments are degraded differently and can be preserved longer than DNA in fossils.

Table S4.4.1. Parameters for calculation of thermal age.

	MAT (°C)	Scale	LGM (°C)	Age (ka)	Thermal age

**Kap København**	-17	2.8	-30	2,000	2700 (500)
**Krestovaya**	-11	2.2	-21	1,200	7000 (500)

Ea=126.96 kJ mol-1, lnA= 41.2 from Lindhal & Nyberg (1972) ref. [61], () denotes the standard deviation.

Table S4.4.2. Theoretical average fragment length for 4 different thermal scenarios.

	Scenario	Average fragment lenghs (bp)					
		k per site per year	1.93 Ma	k per site per year	2.14 Ma	k per site per year	2.6 Ma
1)	_ -17°C for the whole period	1.01E-08	51	1.01E-08	46	1.01E-08	38
2)	0°C for the 0.6 mill years, then -17°C	2.60E-07	2	2.62E-07	2	2.67E-07	1
3)	0°C for the 1.2 mill years, then -17°C	5.01E-07	1	5.03E-07	1	5.08E-07	1
**4)**	0°C for the 1.8 mill years, then -17°C	7.42E-07	1	7.44E-07	1	7.49E-07	1

DNA rate kinetics. Ea=126.96 kJ mol-1, lnA= 41.2 from Lindhal & Nyberg (1972) ref. [61], () denotes the standard deviation.

4.4.1 Chain scission

DNA depurination is believed to lead to strand scission, which is a time dependent exponential decay process displaying pseudo-first order kinetics. Therefore, we can use the estimated rate of depurination based upon the thermal age estimate to predict the extent of DNA chain scission (λ). The rate found by Allentoft et al.[23] is arguably the most relevant here, as unlike Lindhal & Nyberg[61] DNA is decaying in the presence of a mineral (bioapatite) and not suspended in an aqueous solution. Based upon the rate kinetics of DNA from the Moa bone, we would predict DNA survival in both Kap København and Krestovka. Kap København, despite its much greater chronological age, is thermally younger than Krestovka, but both lie within the limit predicted by simplistic model proposed by Allentoft and coauthors[23] and the even more rapid in solution depurination rates estimated by Lindhal & Nyberg[61]. If we, however, use the Allentoft depurination rates then a 30p fragment is assumed to have a half-life of just over 2Ma at a MAT -17 °C.

5. Kap København plant micro and macrofossils

5.1 Microfossils (pollen and spores)

Because of the relatively minerogenic character of the formation the original analysis of the Kap København Formation focused on the macrofossils and not palynology. A cursory pollen analysis was completed in 2013 to compare any recovered microfossils with macrofossil assemblage. Pollen was extracted from six samples from an ~50 cm sequence from Unit B3 at Locality 119. Concentrations were generally low in these sandy sediments. Samples 2 and 3 yielded only 3 and 12 terrestrial grains respectively, but the remaining four samples ranged from 71 – 225 terrestrial grains (mean = 170.25) see Fig. S5.1.1. Pteridophytes including *Botrichium*, *Asplenium*, *Athyrium* were well represented in samples 1, 4 and 6, where along with *Lycopodium* they comprised over 30% of the assemblage. Sample 4 was dominated by herbaceous flowering plants, sedges, and grass while samples 5 and 6 at the top of the sequence had higher frequencies of woody taxa, notably *Betula*.

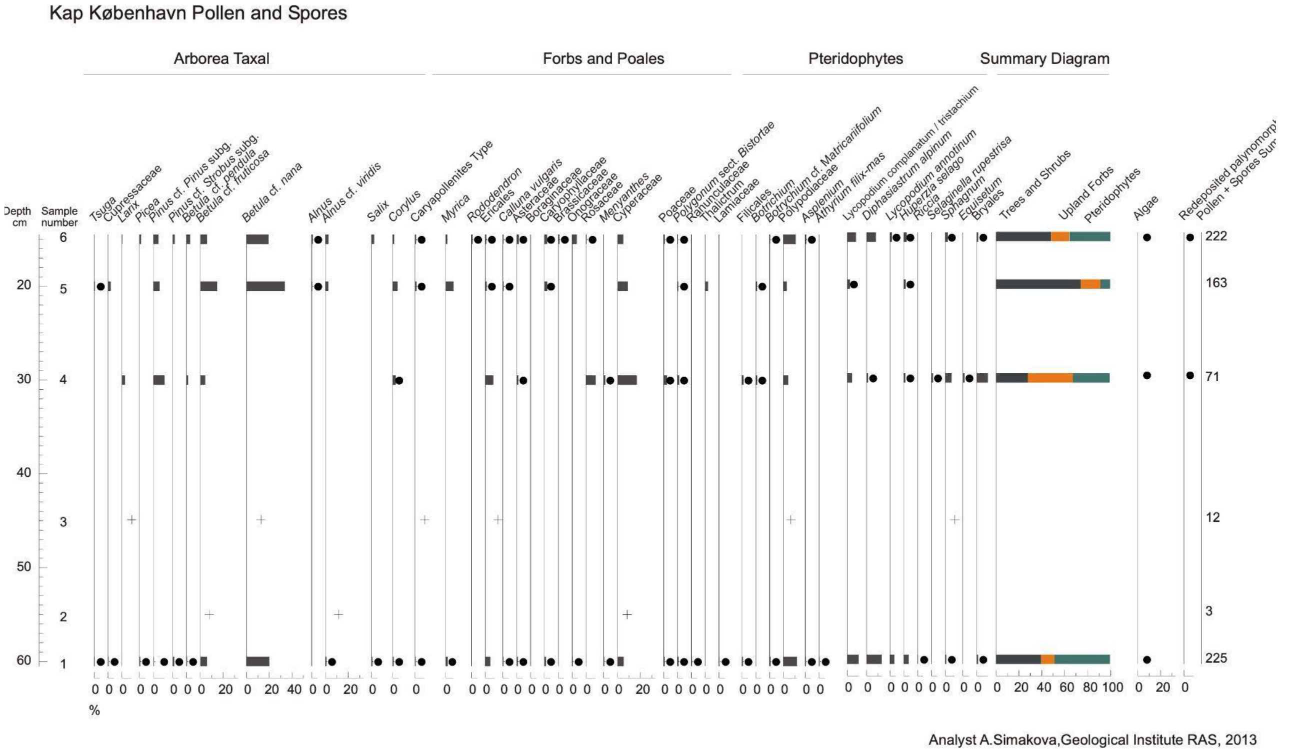

Fig. S5.1.1. Pollen and spore percentage diagram from a 50 cm stratigraphic sequence collected from the unit B3, locality 119 of the Kap København Formation by A. Simakova. The crosses indicate the occurrence of taxon in samples 2 and 3 for which frequencies were not calculated due to pollen sums ≤ 30 terrestrial grains. Bullets indicate percentages ≤ 2. The relatively high frequencies of pteridophytes suggests that these were common in the riparian habitats along the streams, which

transported their spores to the shallow nearshore basin. Because of this alluvial dynamic, the proportions of types need not correlate closely with their actual abundance in the vegetation in terms of biomass or coverage, even when differences in pollen production and dispersion are considered.

Because lake records in boreal forests typically have higher proportions of tree pollen than most of the Kap København samples, the diagram could be interpreted to show a predominance of dwarf shrub and shrub tundra in combination with restricted patches of forest tundra. The increase in arboreal pollen toward the top of the sequence could indicate an expansion of forest tundra but we cannot control for the deposition rate or possible changes in the catchment as the unit accumulated. Because of these uncertainties, we have conservatively compared only the genera represented in the pollen with the macrofossil and ancient eDNA assemblages and not their relative proportions.

5.2 Macrofossils

The plant macrofossils found in the Kap København Formation are some of the oldest and best preserved in the world. Here, large fragments of trees including limbs and small fragile *Larix groenlandii* twigs with cones still attached comprise part of a large and complex assemblage including boreal trees and forbs as well as arctic species[7,10,16,18]. This assemblage of fossils is consistent with the genetic results in which we also find a mixed arctic-boreal plant assemblage that includes some taxa with northern limits more than 1000 km to the south today. From Funder et al.[7] we extracted and digitalized the plant macrofossil assemblage, which can be found Source Data S1 sheet 2.

6. DNA

6.1 DNA sample metadata

Information about samples, extraction batch, sequencing batch, their geological information, number of raw reads, number of reads after trimming and QC can be found in the Source Data S1, sheet 1.

6.2 Database assemblage and taxonomic classification

We used publicly available resources from Genbank NCBI, the nt database (Nov. 2020) and the RefSeq (v.92) as well as the PhyloNorway database[34] and the GTDB (release 95) as reference databases. In addition, to increase the database coverage by metazoans, we screened the NCBI SRA

and the genome databases for boreal taxa that were not included in the RefSeq database. This resulted in a total of 31 genomes, with varying assembly level, of different taxa were added to the already above-mentioned databases see Source Data S1, sheet 4.

6.3 Sequence similarity comparison between samples in kmer space

We initially sought to investigate similarity and dissimilarity between samples between different units and for the data obtained between shotgun and capture enriched samples. For this we used Simka to calculate a k-mer (kmer = 31) spectrum and compute Bray-Curtis ecological distances between them (see Fig. S6.3.1a-d). We find 7 samples from the geological layers 74_B1_83_L1 and 74_B1_83_L3 to fall distant to the other samples on the first component; this placement is likely driven by poor preservation in these layers as later found having few ancient taxa. We therefore excluded these as outliers to explore the structure within the remaining set of samples (Fig. S6.3.1c and Fig. S6.3.1d), and find that between the geological units there are no direct patterns to be recognized. While the samples from units B2 and B3 accounts for most of the variation, which can be explained by the richer ancient taxonomic assemblage (Fig. 3) found, we find the remaining samples from B1 to cluster close with the experimental controls. This again highlights that unit B1 likely had poorer DNA preservation than the other two units.

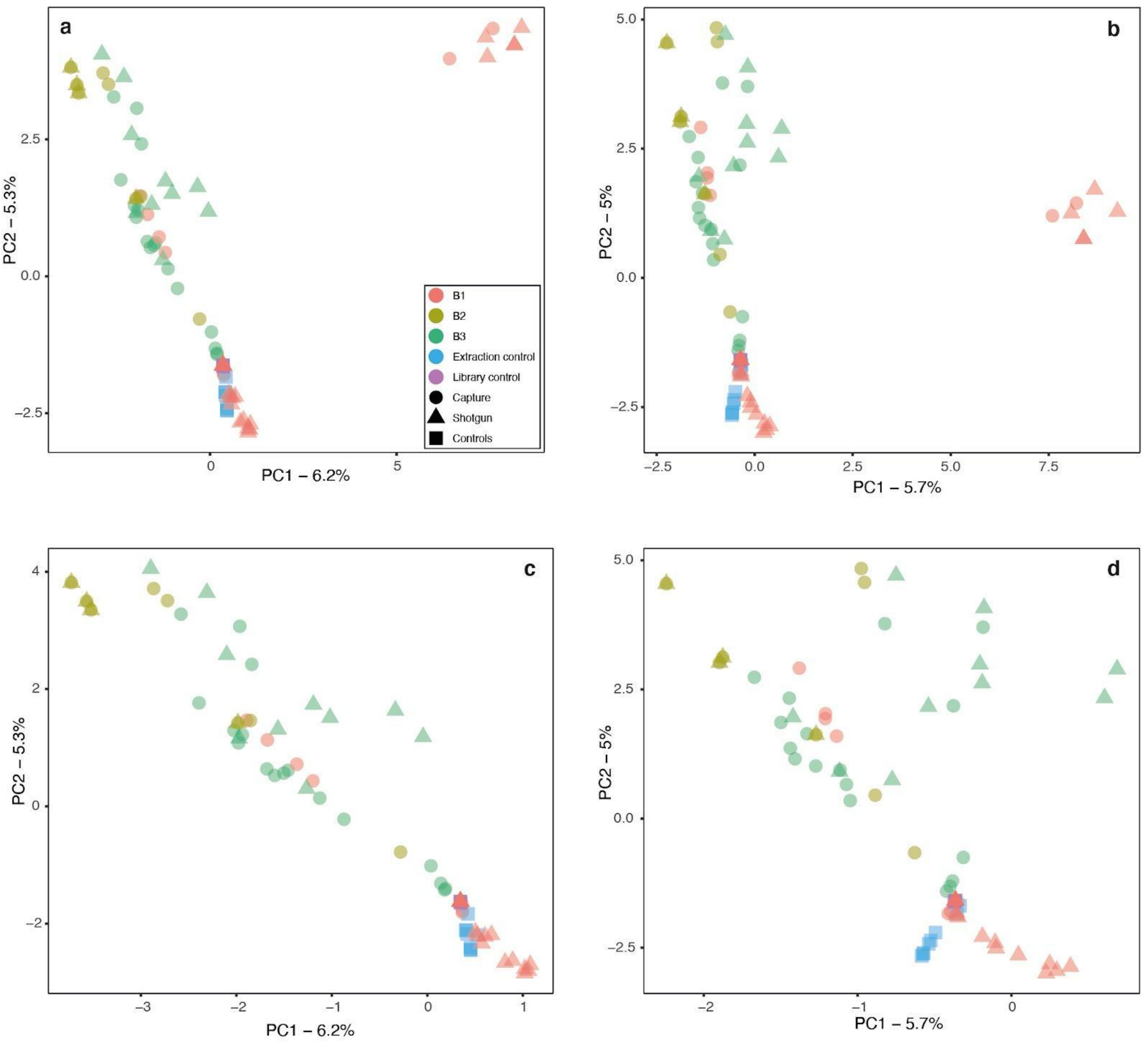

Fig. S6.3.1 principal component analysis of the kmer 31 bray-curtis distances between all samples and controls. a. abundance PCA, **b.** bray-curtis presence-absence, **c.** abundance PCA excluding outliers and **d.** bray-curtis presence-absence excluding outliers.

6.4 Ancient DNA authenticity

6.4.1 Library construct strategy

We used a dual indexing system, to avoid potential index hopping[144], where each barcode was used only once during a library preparation and sequencing run. As this has been shown to be highly effective in avoiding index hopping[144]. Furthermore, to rule out cases where identical index pairs

could have contaminated our libraries post PCR and pre sequencing, we looked at the key taxa, such as the mastodont, hare and reindeer, and found that these were also identified in samples between independent sequencing batches. Considering that batches of samples were built to libraries separately and sequenced separately (in one case we solely used a whole flow cell sequencing batch 3 see Source Data S1). The likelihood of having repeated contamination with matching index combinations to our samples during sequencing on the exact same dates are tiny. Lastly, we requested the sequencing facility about the DNA libraries sequenced in parallel with ours, they didn't contain any elephant, hare or reindeer related DNA. We therefore conclude that the taxa found must have originated from the DNA extracted from our samples.

6.4.2 DNA damage and fragmentation

DNA damage and lengths of reads has long been used as important and independent proxies for ancient DNA authenticity, however, to date this has been a huge undertaking, especially for DNA damage, as each individual taxon had to be assessed manually. Here we use a newly designed fast ancient DNA damage estimator metaDMG[95] which computes nucleotide mis-incorporation and fragmentation patterns efficiently of even highly complex metagenomic datasets. metaDMG takes advantage of the information already contained in the alignment files to compute and statistically evaluate the post-mortem DNA damage for individual taxon or references, and hereby by-passes the need for classifying and splitting reads into individual organisms and hereafter realigning these to individual genomes and parse this data to mapDamage2.0. MetaDMG estimates the average DNA damage at the termini position (D-max) as well as computes a likelihood ratio (λ-LR) which is a test statistic that quantifies how much better the damage model (i.e. more damage in the beginning of the read) fits the data compared to a null model (i.e. constant amount of damage). A low λ-LR value (≈0) means that the damage model was not better at describing the data compared to the null model, whereas the higher the value, the better the fit of the damage model (compared to the null model). The metaDMG (version 0.14.0) was run with parameters -simscorelow 0.95, -simscorehigh 1.0, -weighttype 1 and -max-position 15.

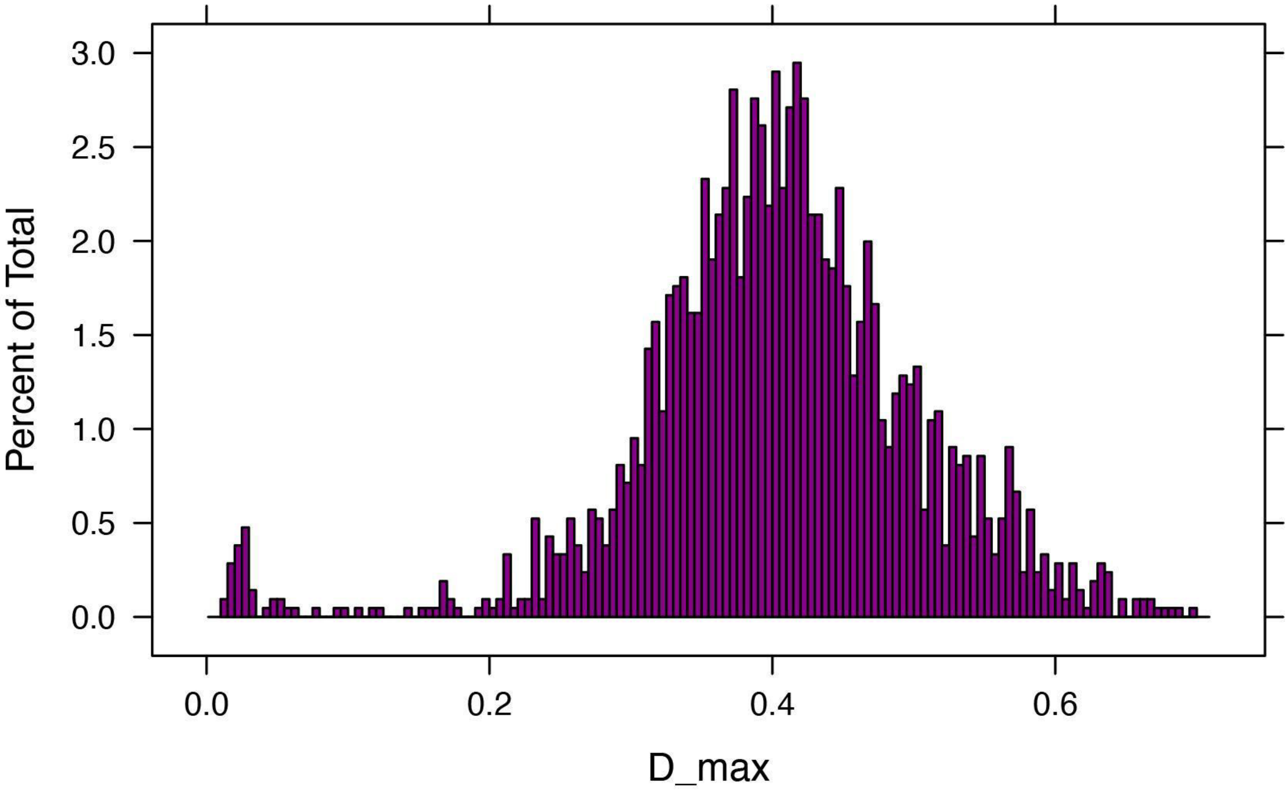

Fig. S6.4.2.1. distribution of DNA damage (D_max) for plant taxa at genus level with ≥ 500 reads assigned.

We explored the DNA damage of the metagenomes from the Kap København formation (Fig. S6.4.2.1-3) by isolated looking at plant taxa with ≥ 500 reads at genus level, thereby excluding microbes whose population potentially can survive in such environments and therefore will not give a true DNA damage estimate. Furthermore, by excluding taxa with less than 500 reads, we limit the noise introduced by taxa with few reads. We next plotted the distribution of DNA damage (see Fig. S6.4.2.1.), which had a median D-max = 0.41 and with the data distributed between 0.25-0.65. In Fig. S6.4.2.2 show C to T and G to A nucleotide substitutions using metaDMG for four key taxa at 15 first positions on the forward and reverse strands, these plots all show, elevated amount of C->T and G->A substitution at the termini's, characteristic for ancient DNA damage. Likewise, is the case for the two marine taxon, the pulicidae and formicidae shown in Fig. S6.4.2.3. From this we set a minimum filter for all samples at all taxonomic levels of D-max ≥ 25% and a likelihood ratio (λ-LR) ≥ 1.5, excluding any taxa and/or samples that did not comply with this threshold. We also extracted all read lengths from reads classified to the key taxa at both nuclear and mitochondrial genome, and find the read lengths, as the damage patterns, are very similar with a tendency to peak towards 30 base pairs (Fig. S6.4.2.4. and Fig. S6.4.2.5.).

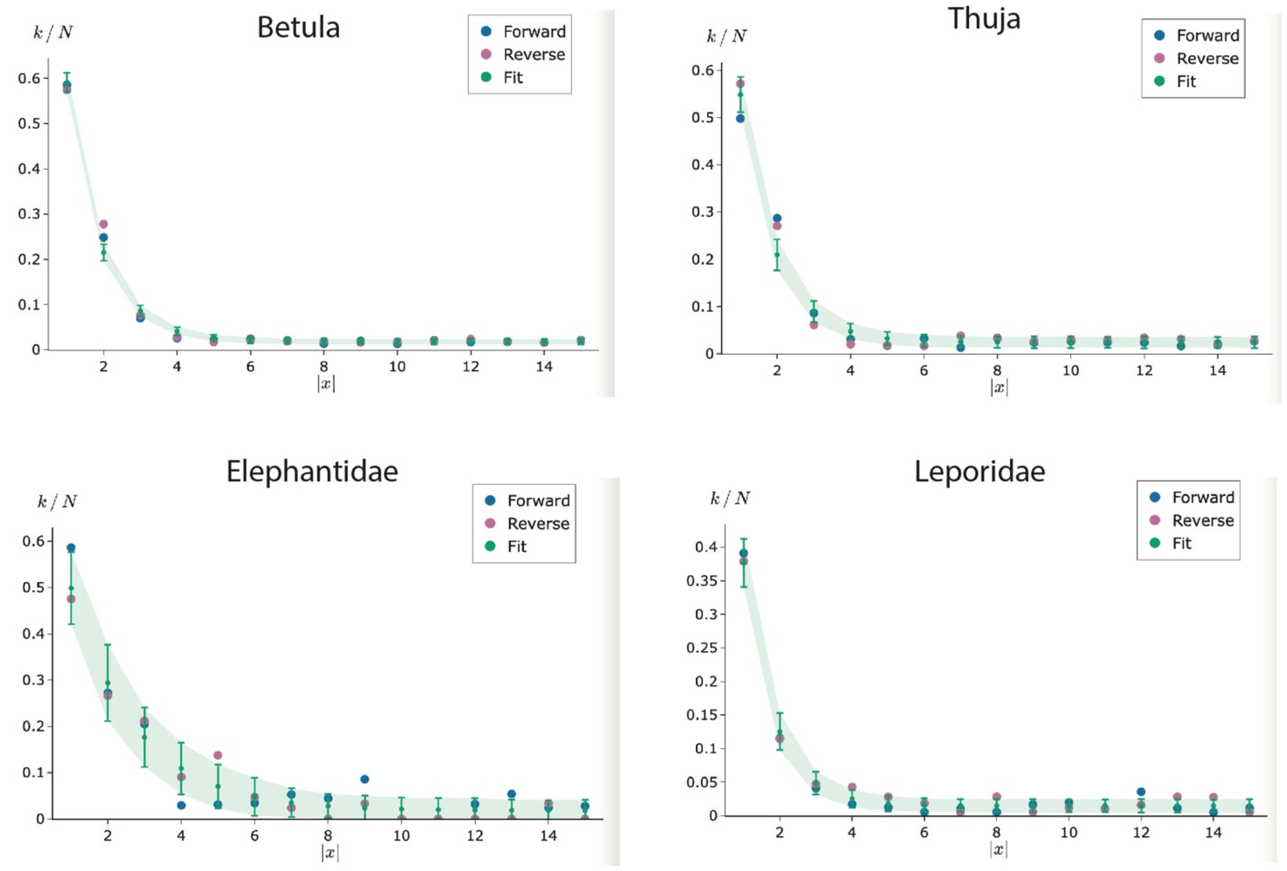

Fig. S6.4.2.2. Position (|x|) specific nucleotide mis-incorporations (k/N) on the forward and the reverse strand for key taxa due to DNA damage. Blue dots are C->T and red dots G->A, green areas indicate the Bayesian fit (including uncertainties). **a.** birch (*Betula*, lambda likelihood ratio (λ-LR): 119.00, Number of reads (n) = 18100), Sample: KapK-205B-Ext-52-Lib-52-Index2). **b.** cedar (*Thuja*, λ-LR: 84.62, n = 1450) **c.** hare (Leporidae*,* λ-LR: 74.76, n = 925, Sample: KapK-12-1-27-Ext-4-Lib-4-Index2) and mastodon (Elephantidae, λ-LR: 51.60, n = 171, Sample: KapK-12-1-35-Ext-12-Lib-12-Index2).

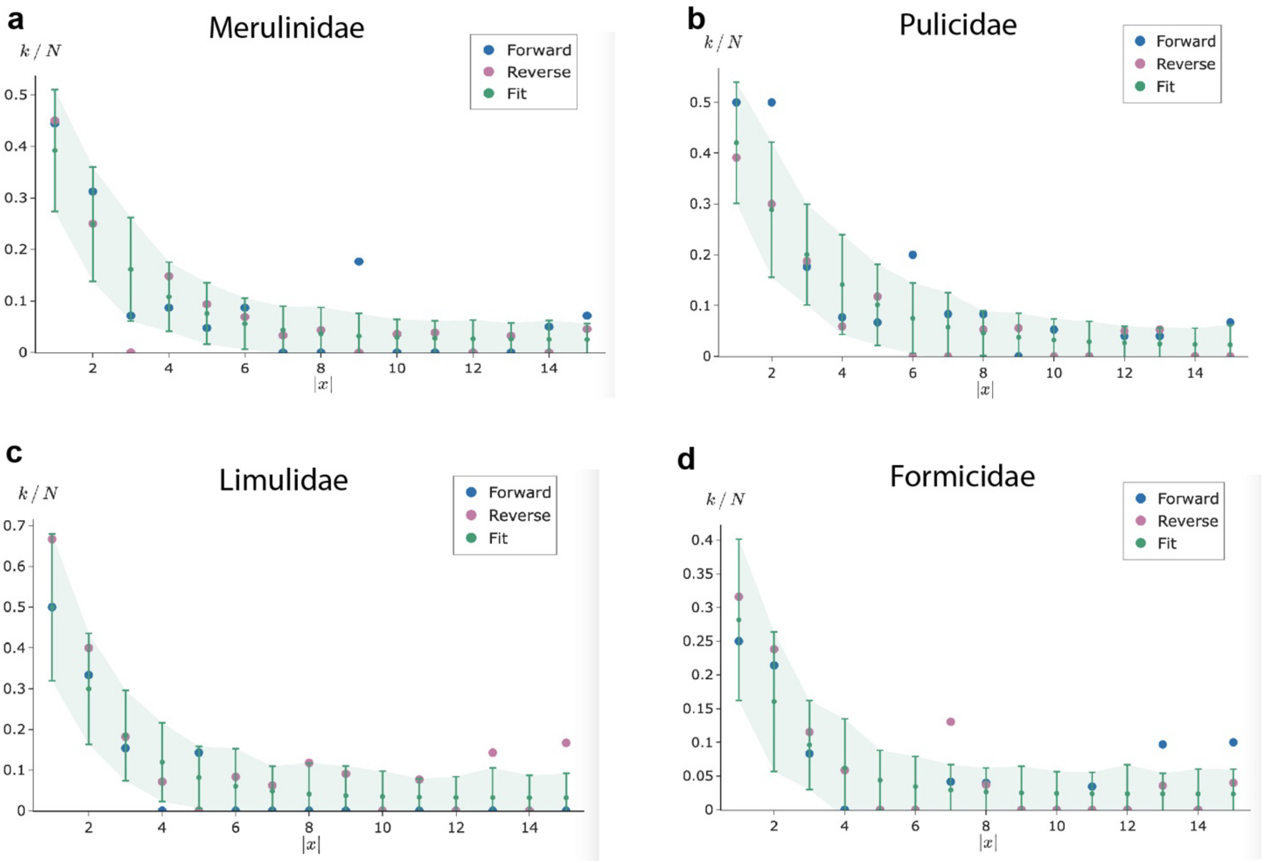

Fig. S6.4.2.3. Position (|x|) specific nucleotide mis-incorporations (k/N) on the forward and the reverse strand for key marine and arthropod taxa due to DNA damage. Blue dots are C->T and red dots G->A, green areas indicate the Bayesian fit (including uncertainties). **a**. Merulinidae (Number of reads (n) = 131, library KapK-12-1-34-Ext-1-Lib-1-Index2), **b**. Pulicidae (n = 139, lambda likelihood ratio (λ-LR) = 37.49, KapK-12-1-52-Ext-34-Lib-34-Index1), **c**. Limulidae (n = 66, λ-LR = 28.91, KapK-12-1-34-Ext-1-Lib-1-Index2), **d**. Formicidae (n = 108, λ-LR = 22.23, KapK-12-1-24-Ext-11-Lib-11-Index2).

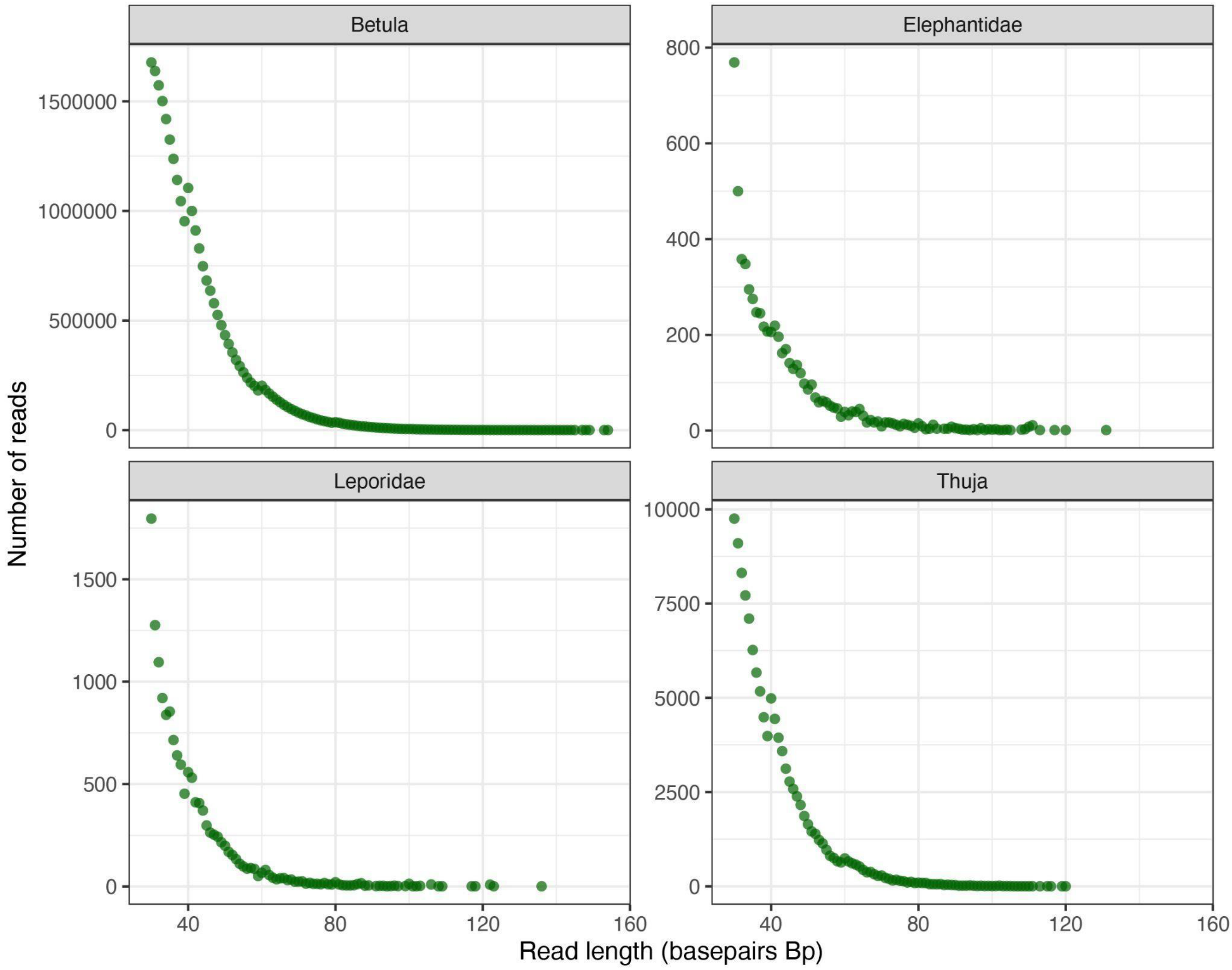

Fig. S6.4.2.4. Read length distributions of all the reads across all samples assigned to the same key taxa shown in Fig. S6.4.2.2

.

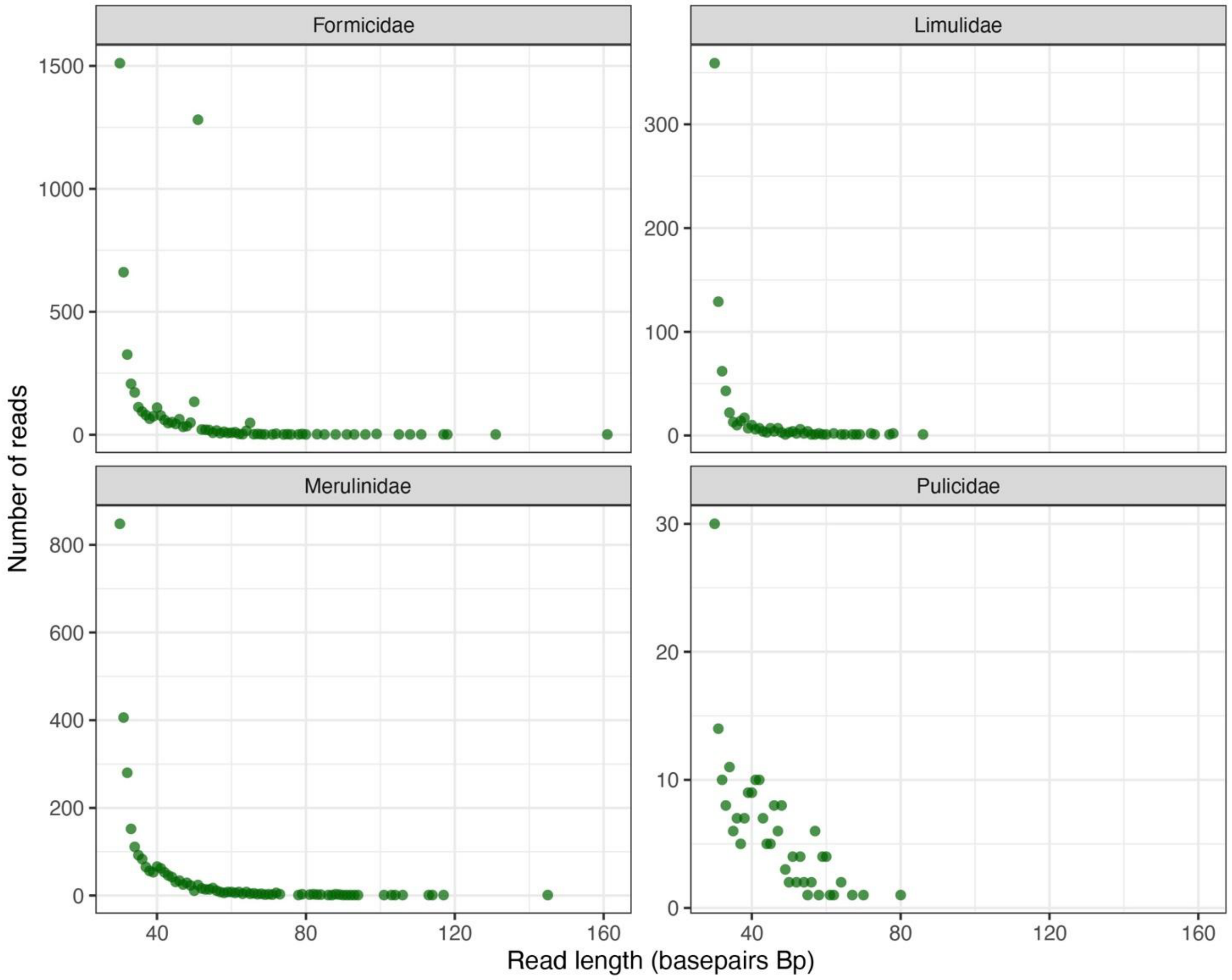

Fig. S6.4.2.5. Read length distributions of all the reads across all samples assigned to the same key taxa shown in Fig. S6.4.2.3.

After filtering the taxa for Dmax ≥ 0.25, LR > 1.5 and read lengths ≥ 30 bp, we explored the abundance of each taxon by counting the number of reads represented at genus level for plants or any read assigned to a lower taxonomic node. We next applied 3 cut-off thresholds to ensure robust taxonomic classifications and profiles. For threshold one (T1), we calculated the median of reads per taxa which were divided by 2 and used this as a cut-off to filter taxa with lower number of reads assigned. This resulted in filtering taxa with fewer reads than 76.5 assigned covering all geological units (B1, B2 and B3). In the second threshold (T2) we used a similar approach but for the total number of reads within a sample, e.g., we calculated the median across the samples and divided this by 2, which we set as a minimum number of reads in a sample (T2 =145249.75). We hereafter

converted the reads to proportion of reads with each sample and parsed all taxa above the 3rd quantile (T3) to be plotted in R setting proportion of reads to be minimum 0.11%. Lastly, we required that each taxon was represented in a minimum of 3 independent samples.

Similarly, we applied T1 and T2 for all reads assigned within metazoans, which resulted in T1 cut-off of 8, 9 and 8 reads, while T2 required 1, 68 or 17 reads per sample for units B1, B2 and B3 respectively. As well as parsed the 3rd quantile (T3), setting proportion of reads to be minimum 26%, 10% and 15%. Lastly, we required that each taxon was represented in a minimum of 3 independent samples.

With these stringent thresholds set, we observed that we filter taxa that due to low read numbers are challenging to verify and therefore also could be false-positive, however we also filter true taxa. For instance, in unit B1, we found taxa such as hare (*Lepus*), mastodon (*Mammut*) and goose (*Anser*) however due to few reads or less than 3 replicates these were removed with the thresholds set. We also observe taxa such as Larch (*Larix*), copepods (*Eurytemora*), wheat stem sawfly (*Cephus cinctus*), sheepshead minnow (*Cyprinodon variegatus*) and bugs (*Nilaparvata*) which showed DNA damage but only appearing in 1 or 2 samples or had too few reads.

This resulted in 102 plant genus and 9 animal families to be parsed and presented in the taxonomic profiles. In Fig. S6.4.2.6-11 we plot the mean read lengths and the Dmax for all samples and for each of the taxa that are presented in Fig.3.

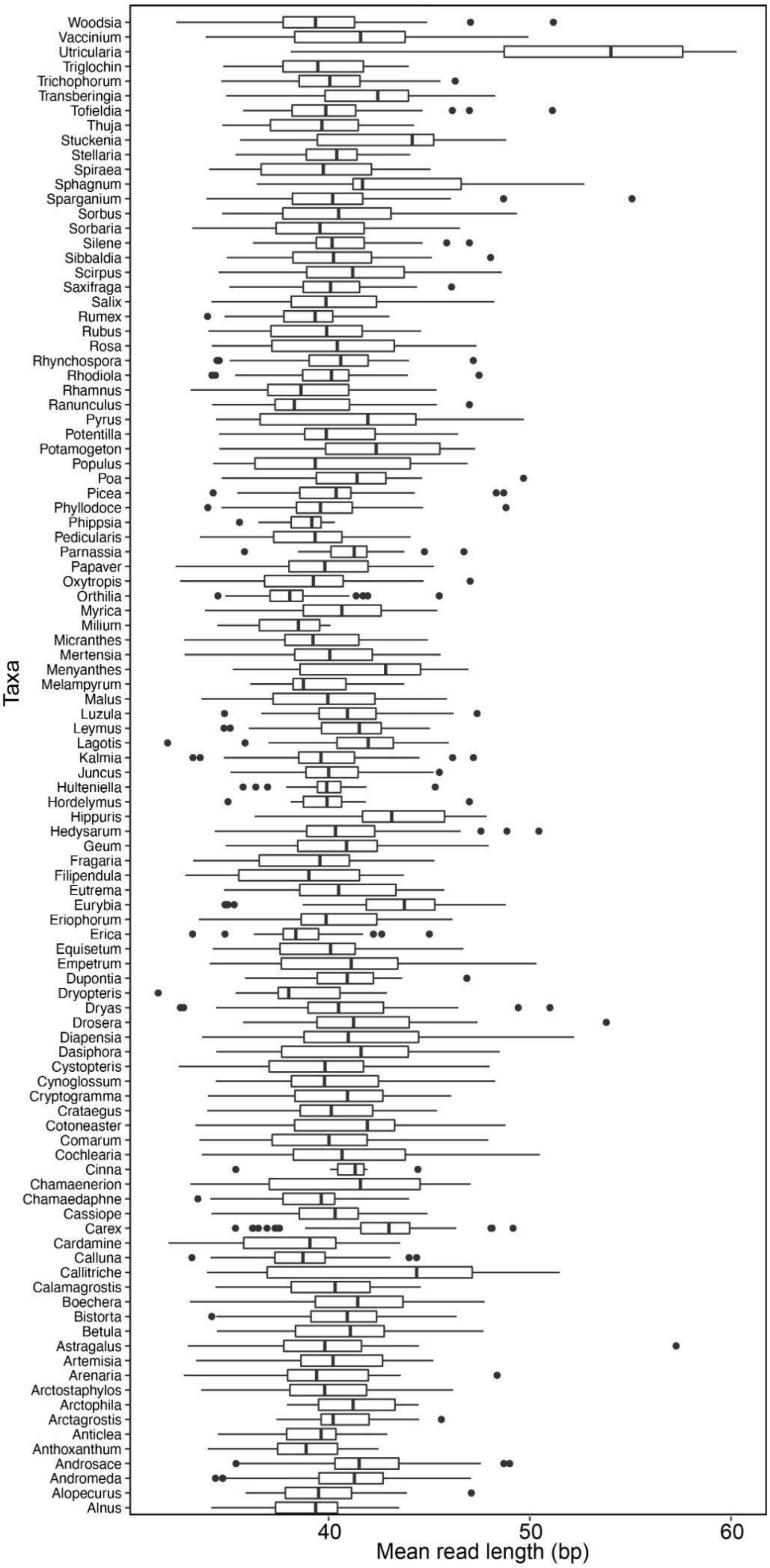

Fig. S6.4.2.6 Boxplot of the mean read length per plant taxa found within each sample. Sample sizes for each plant genus are given in Table S6.4.2.1. The box defines the interquartile range,

and the bold line the median. The upper whisker marks the largest value within 1.5 times the interquartile range above the 75th percentile, and the lower whisker marks the smallest value within 1.5 times the interquartile range below the 25th percentile.

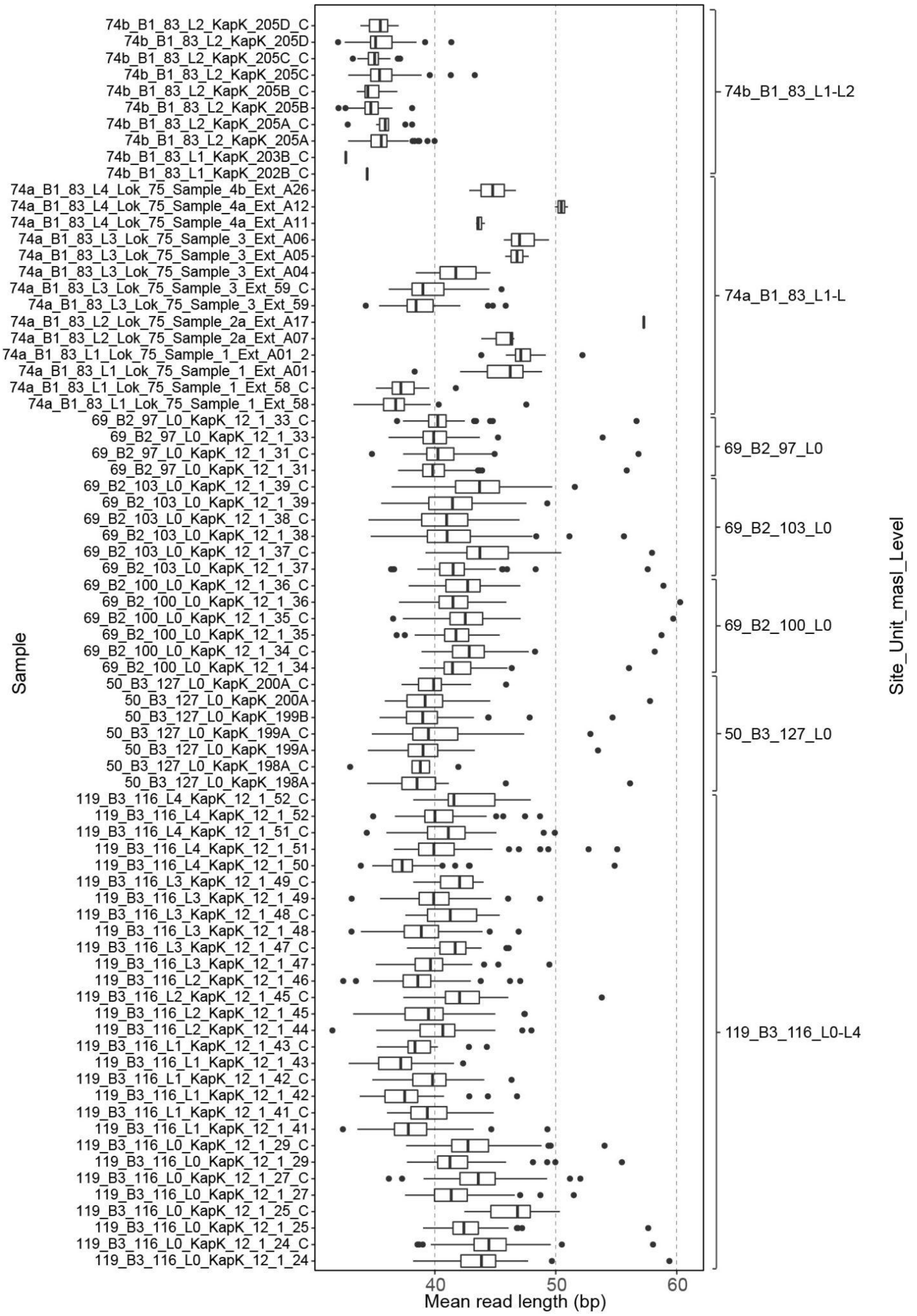

Fig. S6.4.2.7. Boxplot of the mean read length per sample found within all plant taxa. Sample sizes for each sample are given in Table S6.4.2.2. The box defines the interquartile range, and the bold line the median. The upper whisker marks the largest value within 1.5 times the interquartile range above the 75th percentile, and the lower whisker marks the smallest value within 1.5 times the interquartile range below the 25th percentile.

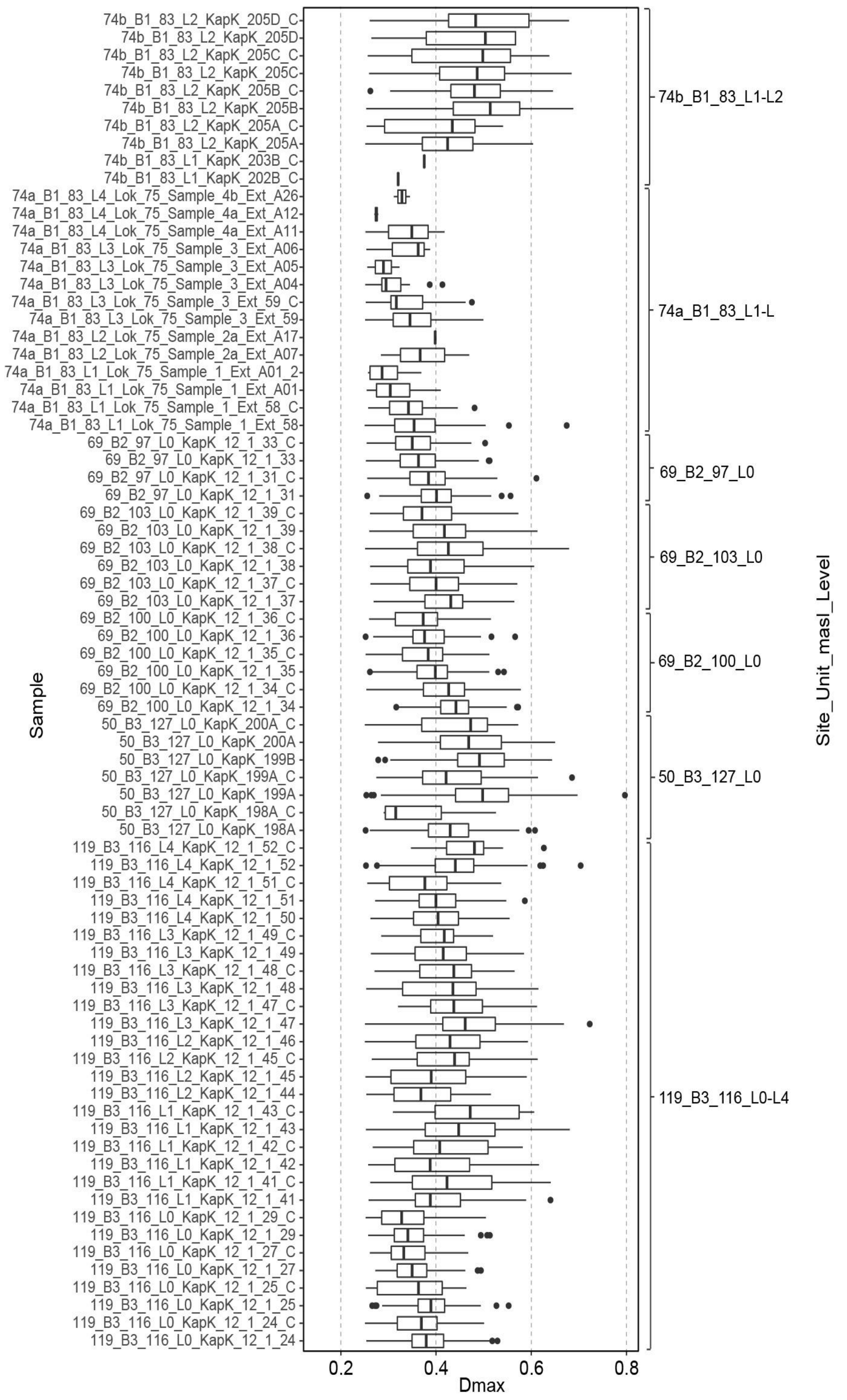

Fig. S6.4.2.8. Boxplot of the average DNA damage (Dmax) of the forward and reverse strand per sample found within all samples. Sample sizes for each sample are given in Table S6.4.2.2. The box defines the interquartile range, and the bold line the median. The upper whisker marks

the largest value within 1.5 times the interquartile range above the 75th percentile, and the lower whisker marks the smallest value within 1.5 times the interquartile range below the 25th percentile.

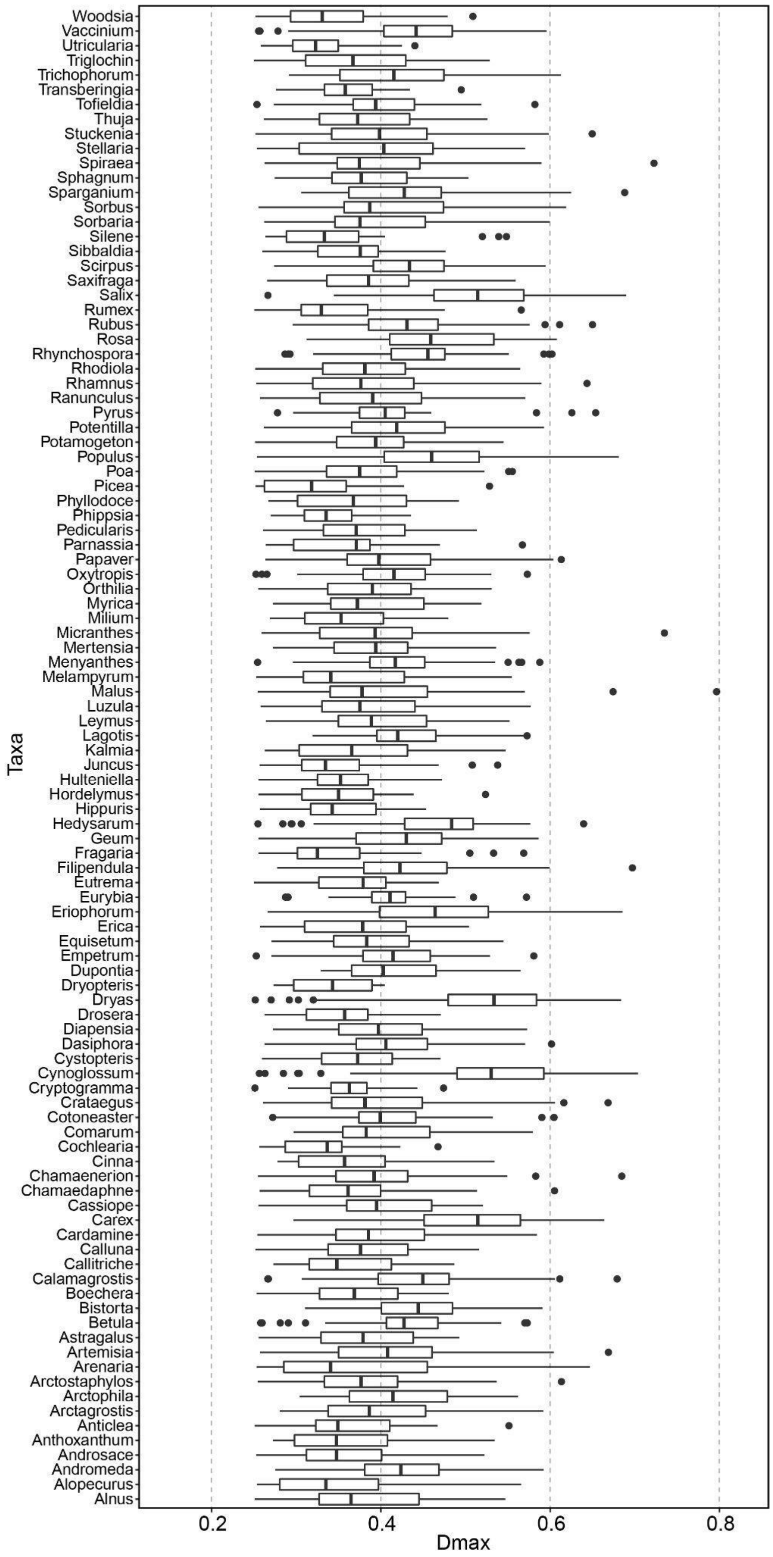

Fig. S6.4.2.9. Boxplot of the average DNA damage (Dmax) of the forward and reverse strand per plant taxa found within each sample. Sample sizes for each plant genus are given in Table S6.4.2.1. The box defines the interquartile range, and the bold line the median. The upper

whisker marks the largest value within 1.5 times the interquartile range above the 75th percentile, and the lower whisker marks the smallest value within 1.5 times the interquartile range below the 25th percentile.

Table S6.4.2.1 Sample sizes for the boxplot figures Fig. S6.4.2.6 and Fig S6.4.2.9.

Plant Genus in Fig. S6.4.2.6 and Fig. S6.4.2.9	Samle size for Fig. S6.4.2.6 and Fig. S6.4.2.9	Plant Genus in Fig. S6.4.2.6 and Fig. S6.4.2.9	Samle size for Fig. S6.4.2.6 and Fig. S6.4.2.9
Woodsia	26	Juncus	43
Vaccinium	66	Hulteniella	20
Utricularia	41	Hordelymus	20
Triglochin	31	Hippuris	25
Trichophorum	40	Hedysarum	57
Transberingia	19	Geum	54
Tofieldia	39	Fragaria	37
Thuja	18	Filipendula	42
Stuckenia	37	Eutrema	23
Stellaria	22	Eurybia	28
Spiraea	44	Eriophorum	57
Sphagnum	11	Erica	36
Sparganium	42	Equisetum	60
Sorbus	47	Empetrum	24
Sorbaria	47	Dupontia	16
Silene	27	Dryopteris	13
Sibbaldia	32	Dryas	74
Scirpus	42	Drosera	27
Saxifraga	51	Diapensia	40
Salix	66	Dasiphora	41
Rumex	34	Cystopteris	25
Rubus	46	Cynoglossum	46
Rosa	46	Cryptogramma	11
Rhynchospora	39	Crataegus	39
Rhodiola	33	Cotoneaster	35
Rhamnus	36	Comarum	43
Ranunculus	35	Cochlearia	24
Pyrus	31	Cinna	10
Potentilla	48	Chamaenerion	37
Potamogeton	43	Chamaedaphne	46
Populus	54	Cassiope	48
Poa	33	Carex	65
Picea	25	Cardamine	32
Phyllodoce	36	Calluna	37
Phippsia	11	Callitriche	27
Pedicularis	50	Calamagrostis	48
Parnassia	28	Boechera	28
Papaver	41	Bistorta	52
Oxytropis	41	Betula	60
Orthilia	37	Astragalus	52
Myrica	44	Artemisia	41
Milium	6	Arenaria	28
Micranthes	42	Arctostaphylos	54
Mertensia	35	Arctophila	15
Menyanthes	52	Arctagrostis	18
Melampyrum	20	Anticlea	31
Malus	35	Anthoxanthum	24
Luzula	29	Androsace	37
Leymus	32	Andromeda	57
Lagotis	35	Alopecurus	22
Kalmia	50	Alnus	45

Table S6.4.2.2 Sample sizes for the boxplot figures Fig. S6.4.2.7 and Fig S6.4.2.8.

Samples in Fig. S6.4.2.7and Fig. S6.4.2.8	Sample size in Fig. S6.4.2.7 and Fig. S6.4.2.8	Samples in Fig. S6.4.2.7and Fig. S6.4.2.8	Sample size in Fig. S6.4.2.7 and Fig. S6.4.2.8
74b_B1_83_L2_KapK_205D_C	15	50_B3_127_L0_KapK_200A_C	20
74b_B1_83_L2_KapK_205D	76	50_B3_127_L0_KapK_200A	79
74b_B1_83_L2_KapK_205C_C	19	50_B3_127_L0_KapK_199B	99
74b_B1_83_L2_KapK_205C	63	50_B3_127_L0_KapK_199A_C	47
74b_B1_83_L2_KapK_205B_C	17	50_B3_127_L0_KapK_199A	90
74b_B1_83_L2_KapK_205B	76	50_B3_127_L0_KapK_198A_C	6
74b_B1_83_L2_KapK_205A_C	9	50_B3_127_L0_KapK_198A	72
74b_B1_83_L2_KapK_205A	65	119_B3_116_L4_KapK_12_1_52_C	9
74b_B1_83_L1_KapK_203B_C	1	119_B3_116_L4_KapK_12_1_52	81
74b_B1_83_L1_KapK_202B_C	1	119_B3_116_L4_KapK_12_1_51_C	24
74a_B1_83_L4_Lok_75_Sample_4b_Ext_A26	2	119_B3_116_L4_KapK_12_1_51	74
74a_B1_83_L4_Lok_75_Sample_4a_Ext_A12	2	119_B3_116_L4_KapK_12_1_50	81
74a_B1_83_L4_Lok_75_Sample_4a_Ext_A11	3	119_B3_116_L3_KapK_12_1_49_C	12
74a_B1_83_L3_Lok_75_Sample_3_Ext_A06	3	119_B3_116_L3_KapK_12_1_49	67
74a_B1_83_L3_Lok_75_Sample_3_Ext_A05	2	119_B3_116_L3_KapK_12_1_48_C	15
74a_B1_83_L3_Lok_75_Sample_3_Ext_A04	19	119_B3_116_L3_KapK_12_1_48	66
74a_B1_83_L3_Lok_75_Sample_3_Ext_59_C	25	119_B3_116_L3_KapK_12_1_47_C	19
74a_B1_83_L3_Lok_75_Sample_3_Ext_59	81	119_B3_116_L3_KapK_12_1_47	79
74a_B1_83_L2_Lok_75_Sample_2a_Ext_A17	1	119_B3_116_L2_KapK_12_1_46	67
74a_B1_83_L2_Lok_75_Sample_2a_Ext_A07	3	119_B3_116_L2_KapK_12_1_45_C	23
74a_B1_83_L1_Lok_75_Sample_1_Ext_A01_2	10	119_B3_116_L2_KapK_12_1_45	68
74a_B1_83_L1_Lok_75_Sample_1_Ext_A01	20	119_B3_116_L2_KapK_12_1_44	27
74a_B1_83_L1_Lok_75_Sample_1_Ext_58_C	13	119_B3_116_L1_KapK_12_1_43_C	18
74a_B1_83_L1_Lok_75_Sample_1_Ext_58	66	119_B3_116_L1_KapK_12_1_43	47
69_B2_97_L0_KapK_12_1_33_C	57	119_B3_116_L1_KapK_12_1_42_C	17
69_B2_97_L0_KapK_12_1_33	97	119_B3_116_L1_KapK_12_1_42	60
69_B2_97_L0_KapK_12_1_31_C	73	119_B3_116_L1_KapK_12_1_41_C	19
69_B2_97_L0_KapK_12_1_31	95	119_B3_116_L1_KapK_12_1_41	49
69_B2_103_L0_KapK_12_1_39_C	65	119_B3_116_L0_KapK_12_1_29_C	71
69_B2_103_L0_KapK_12_1_39	71	119_B3_116_L0_KapK_12_1_29	92
69_B2_103_L0_KapK_12_1_38_C	44	119_B3_116_L0_KapK_12_1_27_C	63
69_B2_103_L0_KapK_12_1_38	59	119_B3_116_L0_KapK_12_1_27	91
69_B2_103_L0_KapK_12_1_37_C	36	119_B3_116_L0_KapK_12_1_25_C	15
69_B2_103_L0_KapK_12_1_37	77	119_B3_116_L0_KapK_12_1_25	93
69_B2_100_L0_KapK_12_1_36_C	73	119_B3_116_L0_KapK_12_1_24_C	79
69_B2_100_L0_KapK_12_1_36	96	119_B3_116_L0_KapK_12_1_24	91
69_B2_100_L0_KapK_12_1_35_C	89		
69_B2_100_L0_KapK_12_1_35	101		
69_B2_100_L0_KapK_12_1_34_C	94		
69_B2_100_L0_KapK_12_1_34	100		

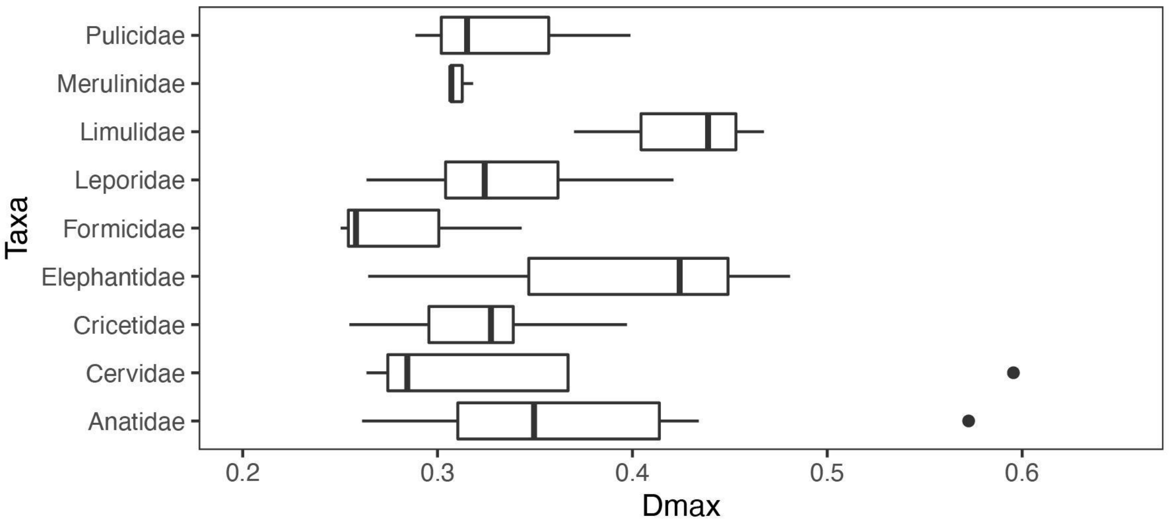

Fig. S6.4.2.10. Boxplot of the average DNA damage (Dmax) of the forward and reverse strand per metazoan taxa found within each sample. Sample size n = 5, 4, 8, 17, 3, 10, 7, 5, 9, for Pulicidae, Merulinidae, Limulidae, Leporidae, Formicidae, Elephantidae, Cricetidae, Cervidae and Anatidae, respectively. The box defines the interquartile range, and the bold line the median. The upper whisker marks the largest value within 1.5 times the interquartile range above the 75th percentile, and the lower whisker marks the smallest value within 1.5 times the interquartile range below the 25th percentile.

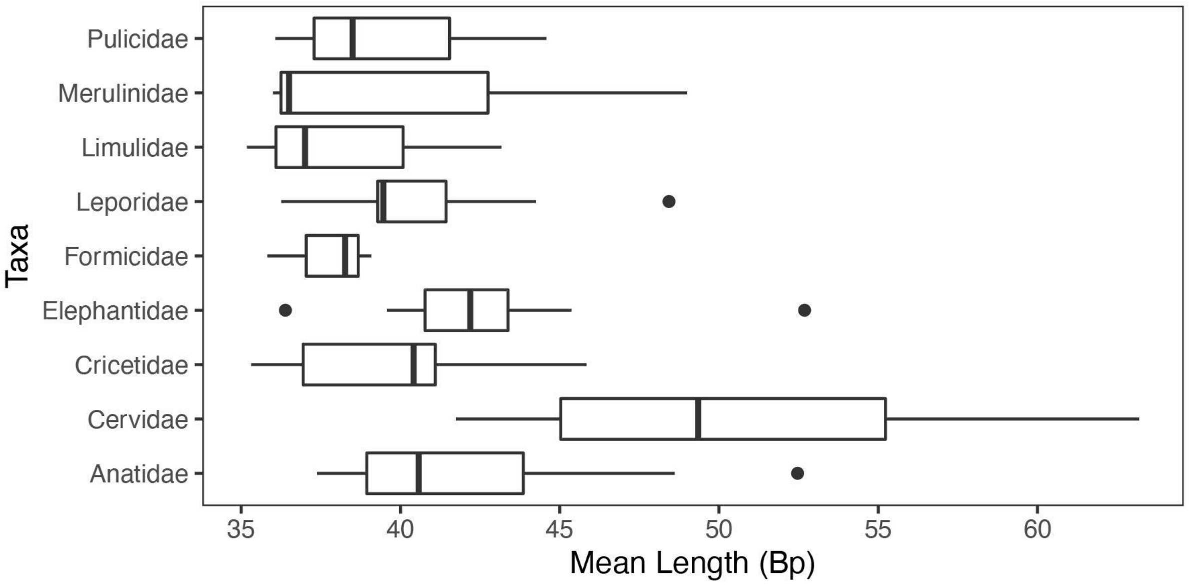

Fig. S6.4.2.11. Boxplot of the mean read length per sample found within all metazoan taxa.

Sample size n = 5, 4, 8, 17, 3, 10, 7, 5, 9, for Pulicidae, Merulinidae, Limulidae, Leporidae, Formicidae, Elephantidae, Cricetidae, Cervidae and Anatidae, respectively. The box defines the interquartile range, and the bold line the median. The upper whisker marks the largest value within 1.5 times the interquartile range above the 75th percentile, and the lower whisker marks the smallest value within 1.5 times the interquartile range below the 25th percentile.

6.5 Capture enrichment

To increase the number of animal mitochondrial reads, we used the animal probe set of the arctic paleo-chip recently published[31] for capture enrichment obtained through Arbor Sciences MyBait®. We find that mitochondrial DNA was enriched in many cases more than 10-fold, in Fig. S6.4.3.1 and Fig. S6.4.3.2. We report the numbers of reads classified to the mitochondria of the key taxa found in Unit B2 and Unit B3. We also compare the overall efficiency of the capture between the key taxa (Fig. S6.4.3.3 and Fig. S6.4.3.4). The efficiency between the individual samples was highly variable, even though they were captured in parallel. The capture bait set proved very efficient and increased the mitochondrial DNA see; Fig. S6.5.1-4.

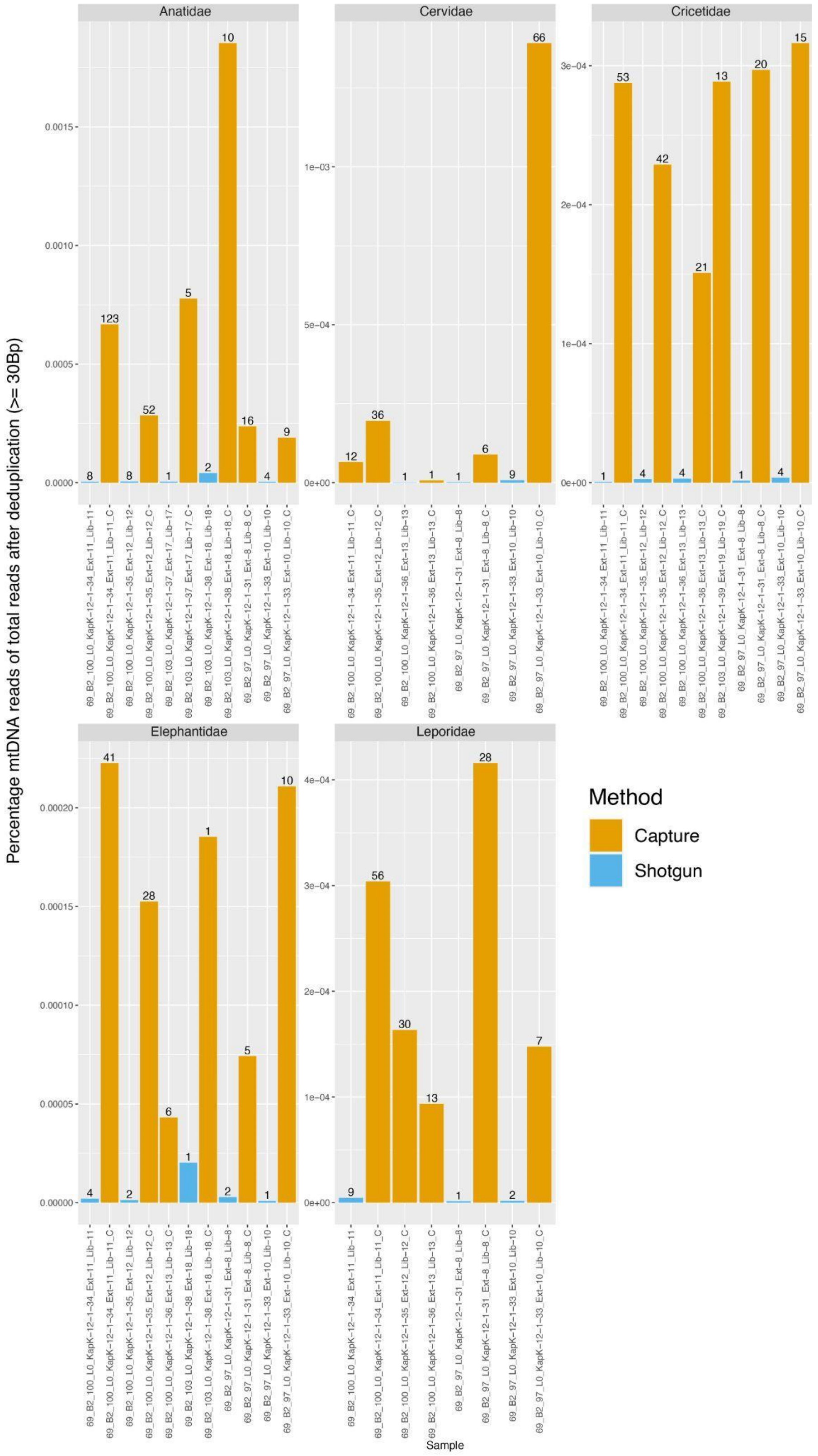

Fig. S6.5.1. Comparison of samples from Unit B2 that were subjected to capture enrichment and those not. Number above bars are total reads after duplicate removal.

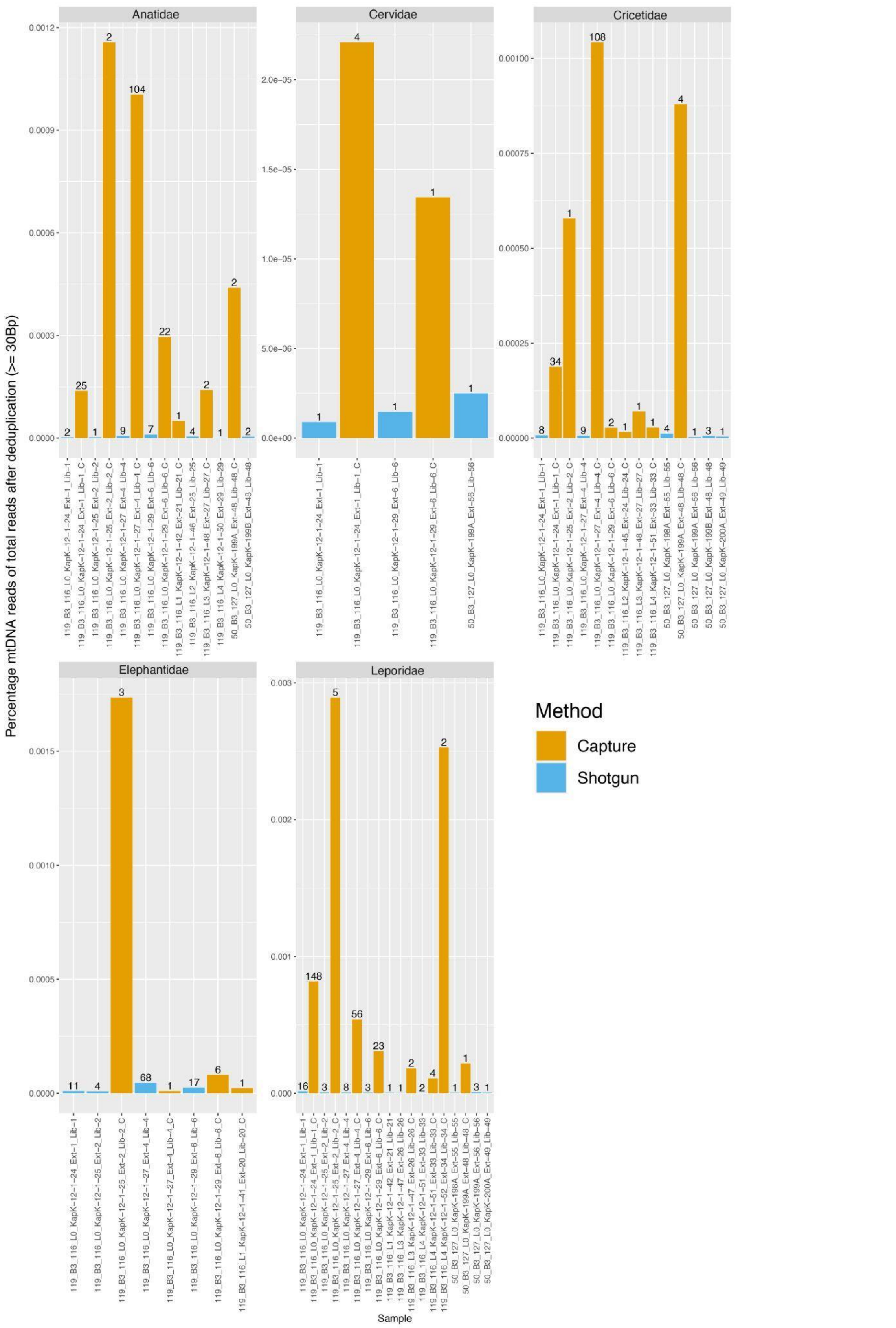

Fig. S6.5.2. Comparison of samples from Unit B3 that were subjected to capture enrichment and those not. Number above bars are total reads after duplicate removal.

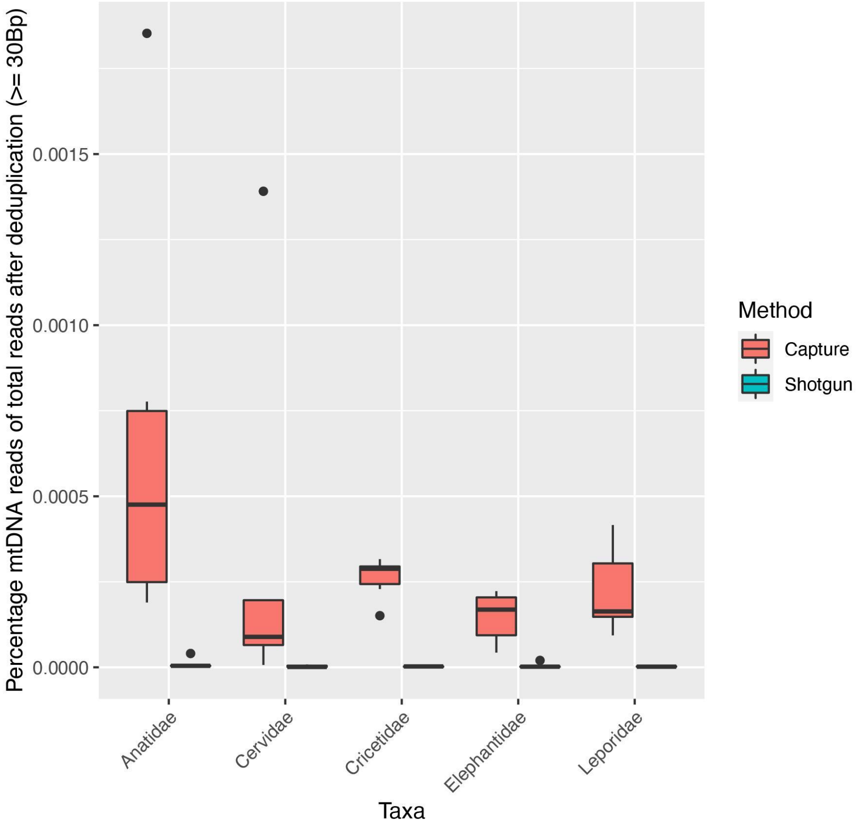

Fig. S6.5.3. Comparison of samples from Unit B2 that were subjected to capture enrichment and those not. Sample sizes for the captured boxplots are 6, 5, 6, 6 and 5 for Anatidae, Cervidae, Cricetidae, Elephantidae and Leporidae, respectively. Sample sizes for the shotgun boxplots are 5, 3, 5, 5 and 3, following the above order. The box defines the interquartile range, and the bold line the median. The upper whisker marks the largest value within 1.5 times the interquartile range above the 75th percentile, and the lower whisker marks the smallest value within 1.5 times the interquartile range below the 25th percentile.

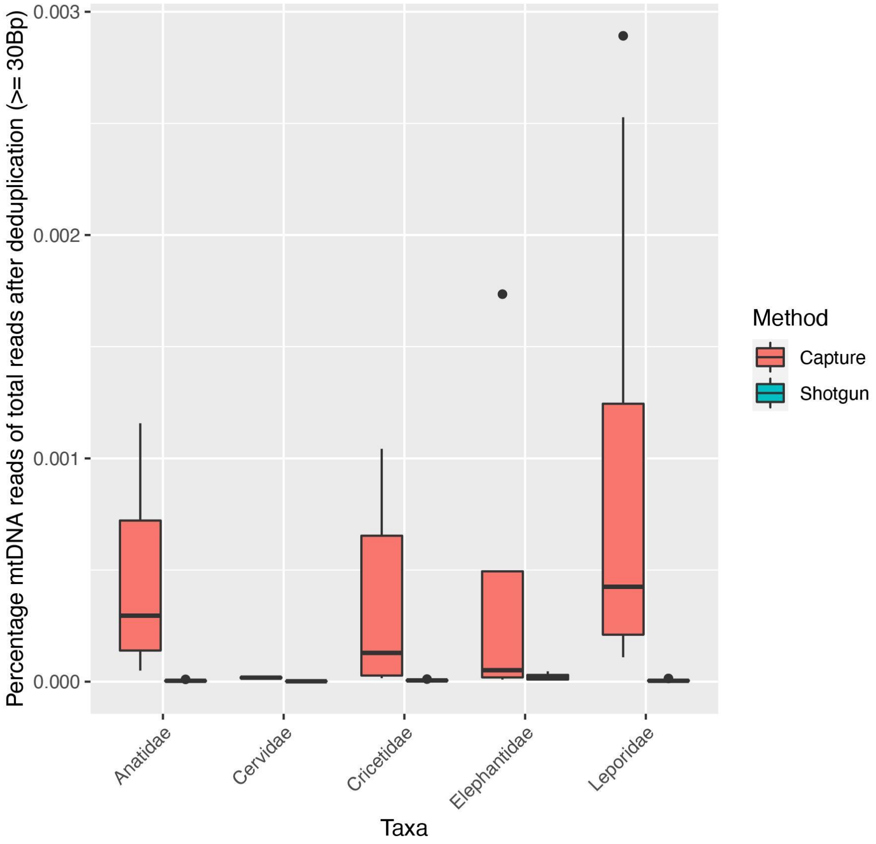

Fig. S6.5.4. Comparison of samples from Unit B3 that were subjected to capture enrichment and those not. Number above bars are total reads after duplicate removal. Sample sizes for the captured boxplots are 7, 2, 8, 4 and 8, for Anatidae, Cervidae, Cricetidae, Elephantidae and Leporidae, respectively. Sample sizes for the shotgun boxplots are 7, 3, 6, 4 and 10, following the above order. The box defines the interquartile range, and the bold line the median. The upper whisker marks the largest value within 1.5 times the interquartile range above the 75th percentile, and the lower whisker marks the smallest value within 1.5 times the interquartile range below the 25th percentile.

6.6 Comparing plant taxonomic profiles

We used principal component analysis (PCA) to test for patterns between units, sites and the driving vectors (plants only), Fig. S6.6.1. and Fig. S6.6.2. We find that most samples are clustering by site rather than by unit, which likely explains that there are slight depositional differences between these. On PC1 site 50 accounts for most of the variation in the dataset, while PC2 explains the variation

between the remaining sites. The structure of the variance does not seem to correspond directly to any single environmental variable like temperature, effective precipitation, or substrate (Fig. S6.6.2.).

In addition, we reran our data setting the mean read length of ≥ 35bp to check whether the plant composition was affected by the read length and to make sure these were not affected by spurious mappings (Fig. S6.6.4.). Our reanalysis found that only one plant taxon (Picea) was not present compared to the taxonomic profile with threshold set at ≥ 30 bp (Fig. 3a). As *Picea mariana* is also found in the macrofossil record in the Kap København formation we find no argument to set the cut-off threshold in this study to 35 bp. Though we acknowledge that spurious mapping can occur, we argue that our databases and the competitive mapping ensure that this is not an issue at the family/genus/species taxonomic levels but can inflate the numbers at higher taxonomic levels, such as root and phylum (see also reply to comment below).

We also performed a test comparing our database with databases of slightly different composition, to test the effects of having the Arctic - Boreal database nuclear DNA as well as chloroplasts in there. First, we build a database consisting of chloroplasts from both RefSeq and the arctic boreal chloroplast only, and secondly, we build a database in which we exclude the arctic boreal database entirely (both nuclear and chloroplast). The alignments to each of the databases were hereafter parsed using the workflow described in the manuscript's method section. To ease comparison between the results, we compare the summed number of reads to each taxon across all samples for the different database builds (See Fig. S6.6.5.).

We find the taxonomic profiles to be highly similar when excluding nuclear reference material, and aligning only to chloroplasts of the RefSeq and arctic boreal databases (Fig. S6.6.5a). In fact, 46% (16 of the 35) most abundant taxa found when using nuclear references are also found when restricting to the chloroplasts. A total of 58 % of the 35 genera found when restricting to cpDNA were found among the 102 genera found using the complete databases (Fig.3a, main text), and the remaining 42 % were present in the dataset but between the genera that were below the filtering thresholds set. All genera found today can be found in the North American (including Greenlandic) flora. Lastly, 17 % and 25% of the genera found are not part of the Arctic-Boreal plant database, for the nuclear and the chloroplast taxa, respectively.

Less similar is the taxonomic profile when the database is restricted to all RefSeq plastids only, here 83% of the genera were not found among the most abundant plants when including the arctic boreal nuclear (+ plastids) database. While the majority can be found on the North American continent today, 18% of the genera cannot and are today found at more southern latitudes, such as the *Flacourtia sp.,* which is part of the Salicaceae family but grows in the Asian and African tropics and subtropics. This is more likely DNA reads from the genera Salix (potentially ancestral Salix glauca or related) which are widespread in the north today - and part of the new contribution by the Arctic - Boreal plant database. We argue that a similar process is the case for the other exotics (Fig. S6.6.5b).

In summary, the taxonomic profiles obtained with nuclear and plastid as well as plastid only from the arctic and boreal as well as RefSeq are very similar. Which is the more correct way of presenting the data is still unknown. But as we do not attempt to convert the proportions to abundances in the landscape (which we currently have no method for) we argue that both are equally good and therefore present the data where the nuclear data is included. From the abundances of genera and the summary statistics of the database content above, we conclude that the addition of the arctic and boreal database does not drive the data in an arctic boreal mixed species community, but that this is the nature of the data.

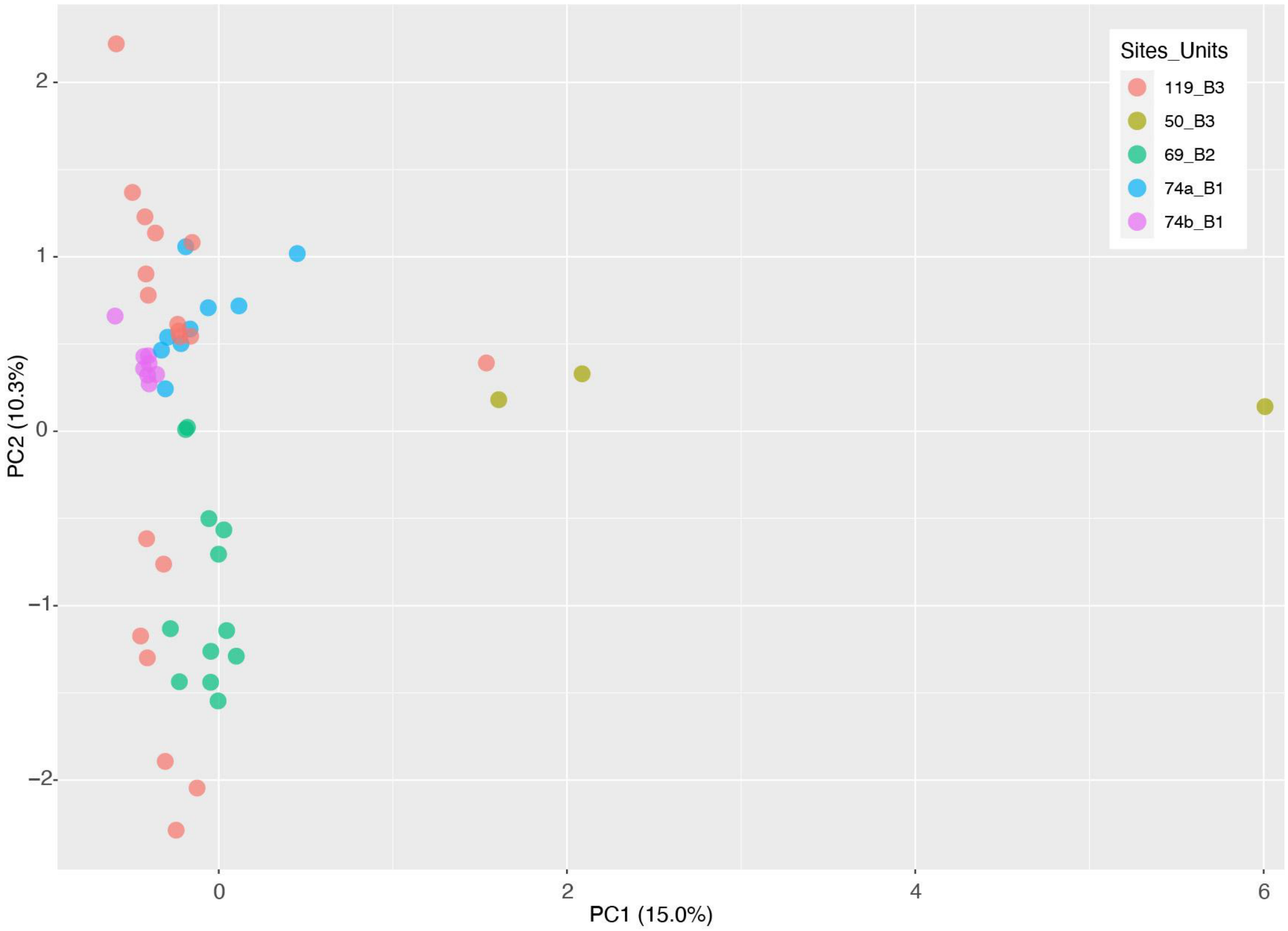

Fig. S6.6.1. Principal component analysis (PCA) of damage filtered plant taxonomic profiles, colored by sites.

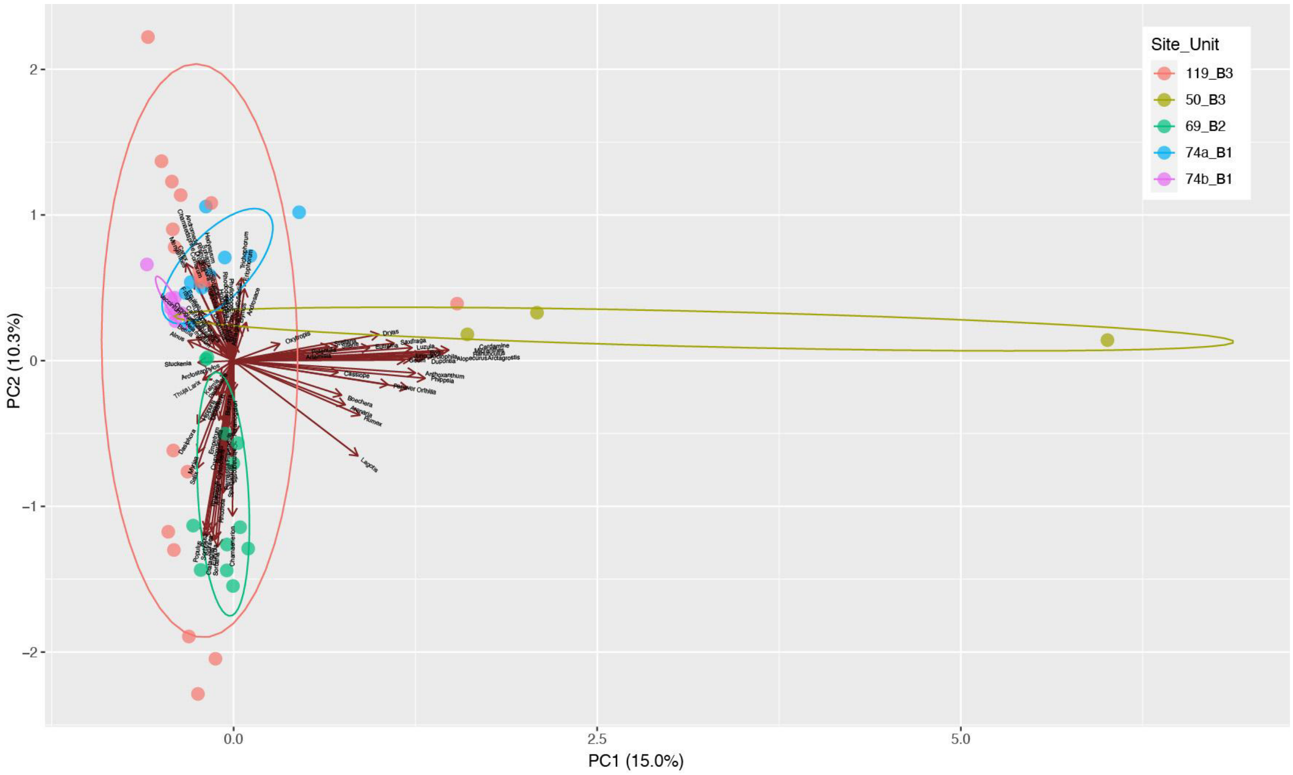

Fig. S6.6.2. Principal component analysis (PCA) of damage filtered plant taxonomic profiles. Colored by sites, added driving vectors (dark red arrows) and added group ellipses using ggbiplot (ellipse= TRUE) in R.

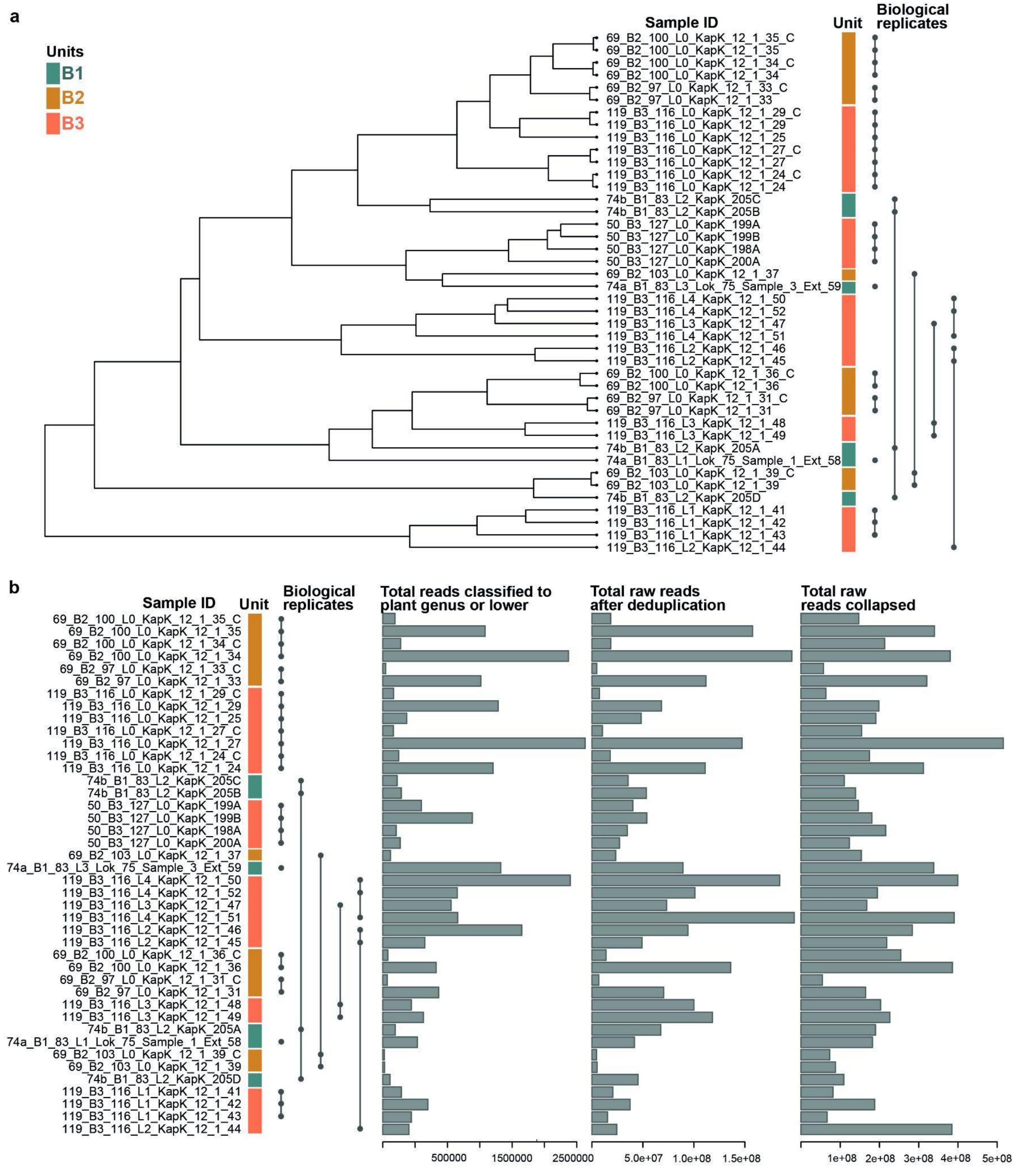

Fig. S6.6.3. Comparison of biological replicates. a. Hierarchical clustering using Euclidean distances (in heatmap.2) of the plant taxonomic profiles (Fig. 3) for each sample, with the corresponding sample names, units and lines connecting the replicates. **b.** Read counts of taxa classified, total reads before and after duplicate removal in relation to the biological replicates.

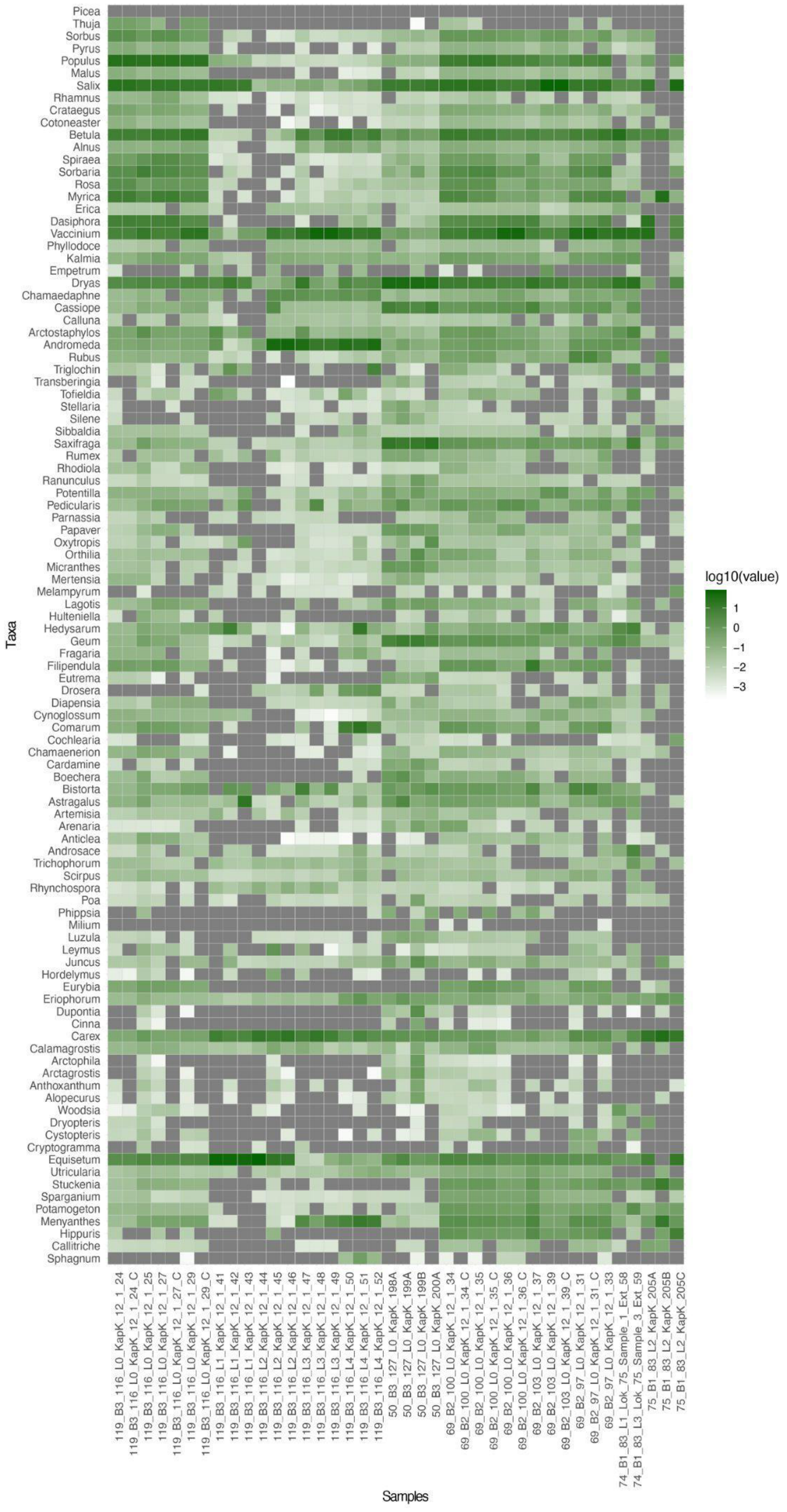

Fig. S6.6.4. Plant genus profile with mean read length 35Bp or more.

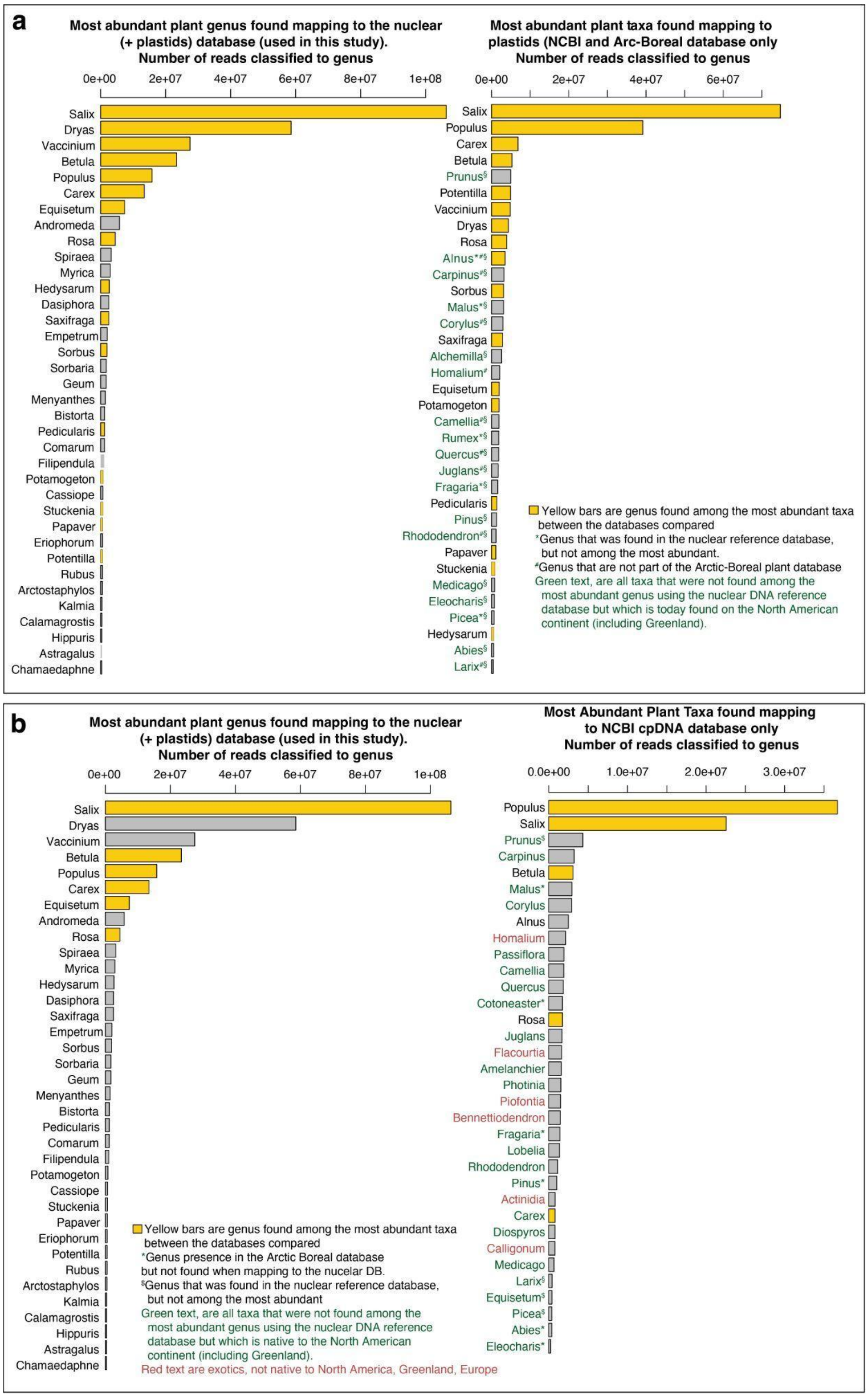

Fig. S6.6.5. Most abundant plant genus identified compared to two databases. a. Comparison of the abundant taxa found in using the databases presented in the manuscript with a database

limited to chloroplasts only and b. Comparison of the taxa found with the databases described in the main text with the RefSeq chloroplast database excluding the Arctic - Boreal database.

6.7 Sensitivity and coverage analysis for the phylogenetically placed taxa.

Spurious mapping is a ubiquitous issue in all ancient DNA studies and we therefore tested the uniquely classified mitochondrial reads aligned to the key taxa used in the phylogenetic analysis in the Kap Copenhagen dataset. First, we look at the read distribution across mitochondrial genomes, as it is well accepted that reads should fall near to randomly across the genome. In Fig. S6.7.1. we show that this is in fact the case. We next compared the average number of mismatches to the respective mitochondrial references per base pair across the reads lengths and compared these to the one million year-old sequence data of a mammoth[21] (Accession: ERR5032135_1). However, we found that these libraries had been treated with a Uracil-DNA Glycosylase enzyme to reduce mismatches occurring from post mortem DNA damages, and we therefore also included the non-treated sequence data from a 13.5kyr old mastodon[145] (Accession: SAMEA104469193). The main reason for performing this analysis is that the rate of allele mismatches per base pair should be constant as a function of read length in absence of spurious alignments. On the other hand, if we had many spurious alignments, we should also observe a marked increase in the rate of allele mismatches in shorter read lengths. We find that the allele mismatches across all taxa including the mastodon from ref. [145] are highly comparable, with no marked increase from the constant rate in the allele mismatches in the shorter read lengths (Fig. S6.7.2). However, we note that the mismatch rate across different lengths do vary more for the taxa in our data, which we conclude is due to the low number of read counts (< 441 reads) compared to the much larger datasets of both the mastodon and mammoth bone data (> 11.400 reads). In fact, when we combine the counts from our mtDNA sequences (totalling 1.451 reads) this variation becomes much less pronounced. We also note that these allele mismatch counts are directly comparable to the plant chloroplast results discussed below.

Similarly, we performed the same analysis for the assembled chloroplast genomes of the key plant taxa. We observe that the allele mismatches across the different read lengths show little variation compared to the mitochondria, which is explained by the larger number of reads (> 39.000 reads) (Fig S6.7.3.). From this we determined that spurious mapping does not seem to be an issue for these metagenomic assembled mitochondria and therefore identify a minimum cut-off read length of 30Bp and speculate that this is likely a product of the conservative competitive mapping approach and the

naïve least common ancestor algorithm we use parsing only 95% similar sequences for the taxonomic profiling. In particular, if a read is likely to spuriously map to one genome in our databases, it likely can align to other reference genomes as well and is therefore placed at a high taxonomic level.

We checked potential coverage and depth to the nuclear DNA of the focal species found. By mapping all reads classified as Capreolinae to the published nuclear genome of *Rangifer tarandus caribou* (Accession number: GCA_019903745.1). This resulted in 3,488 reads mapping to the nuclear genome with a coverage of 4.6e-05, covering a total 111,400bp. We also mapped all reads classified as Elephantidae to the published nuclear genome of *Mammut americanum* (Accession number: SAMEA104469184). A total of 7,450 reads mapped to the nuclear genome with a coverage of 8.7e-05, and covering totally 277,942bp.

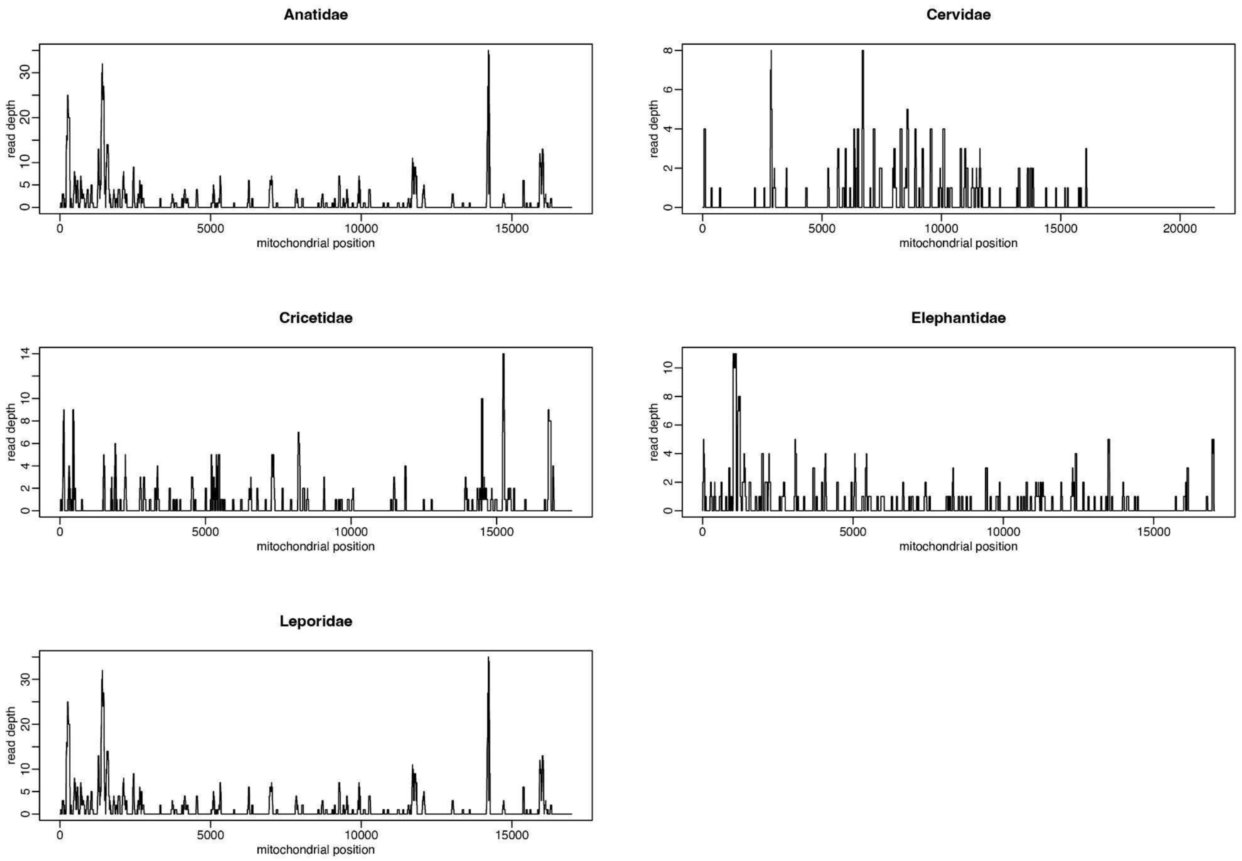

Fig. S6.7.1. Read distribution on the mitochondrial genomes of the key animal taxa.

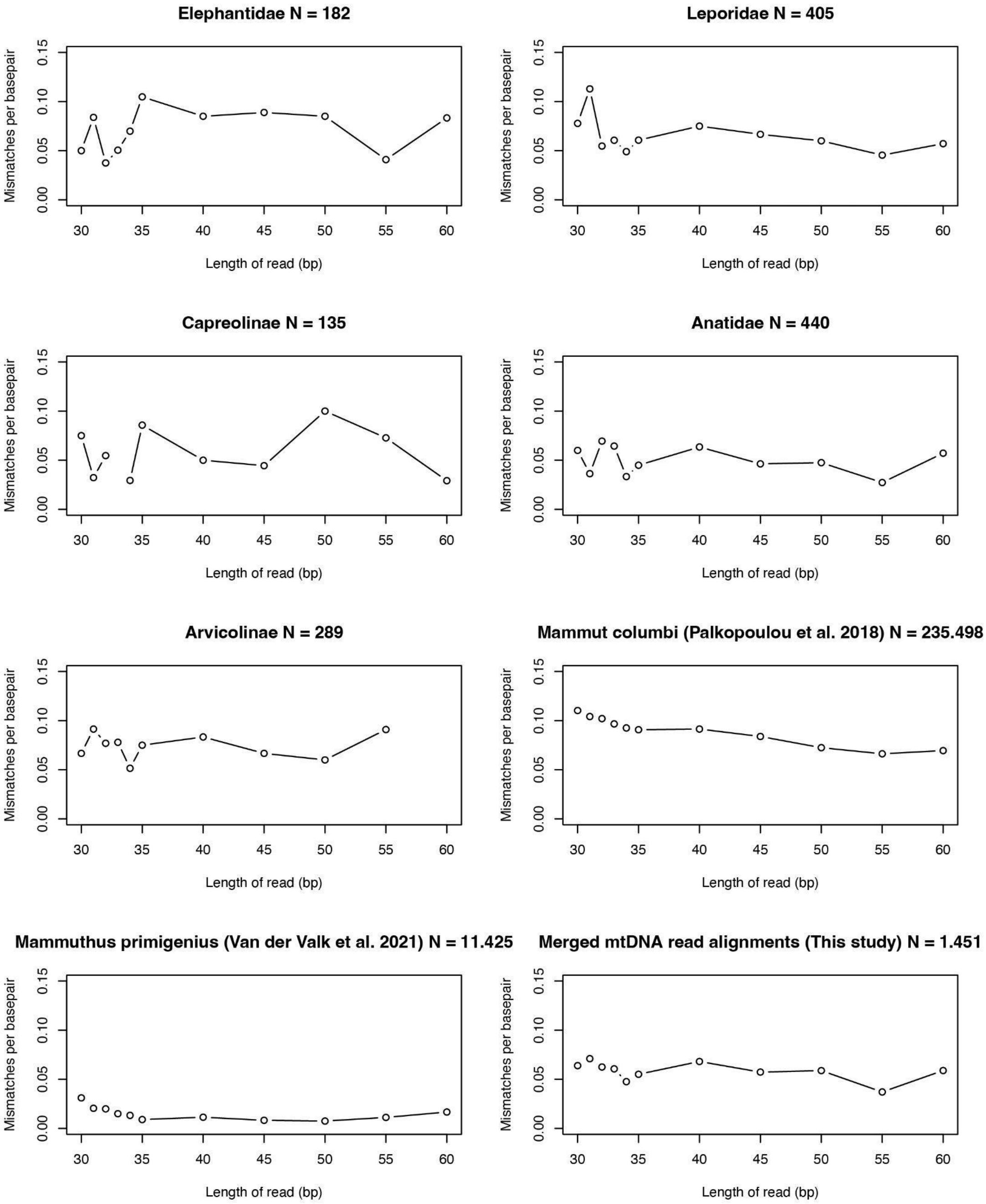

Fig. S6.7.2. Average number of mismatches to the mitochondrial reference per base pair across read lengths for animal key taxa identified in our samples and comparing these with average mismatch numbers obtained from a subset of the ~1.2 Ma old *Mammuthus primigenius* sequence data[21] as well as a subset of the younger (~13.5kyr old) mastodon data[145], following the method as described in https://github.com/stefaniehartmann/readLengthCutoff.

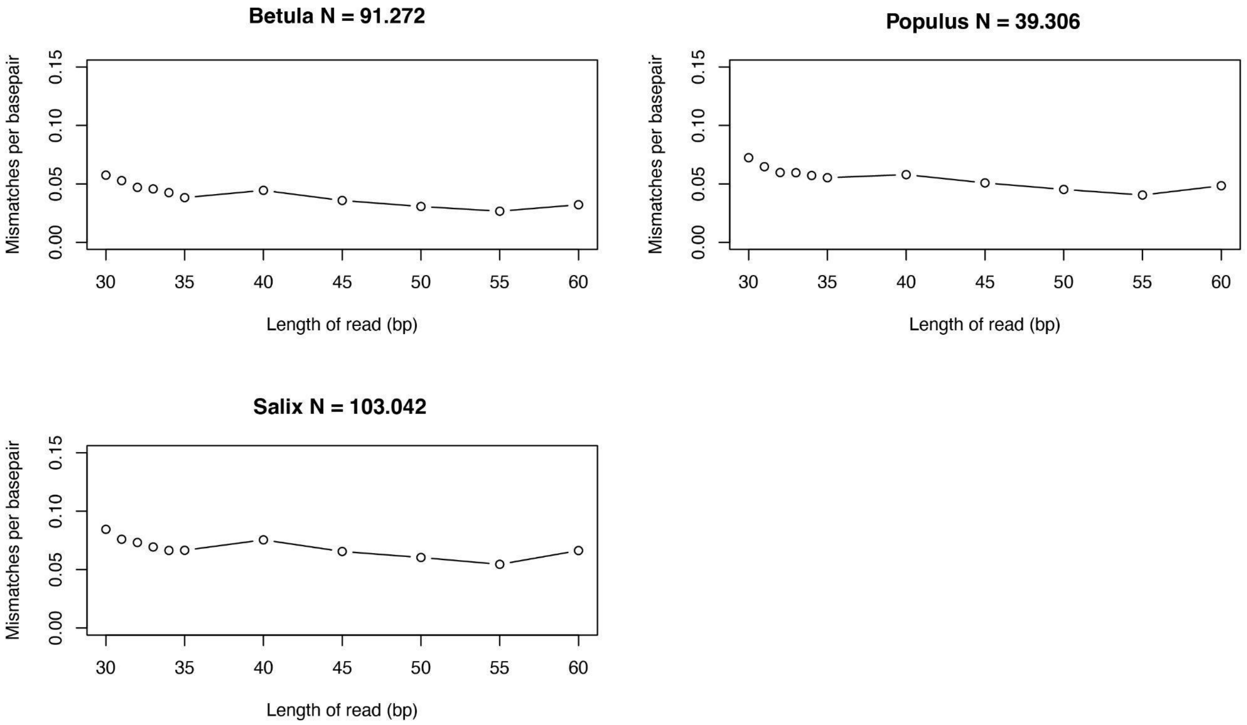

Fig. S6.7.3. Average number of mismatches to the chloroplast references per base pair across read lengths for key taxa identified in our samples.

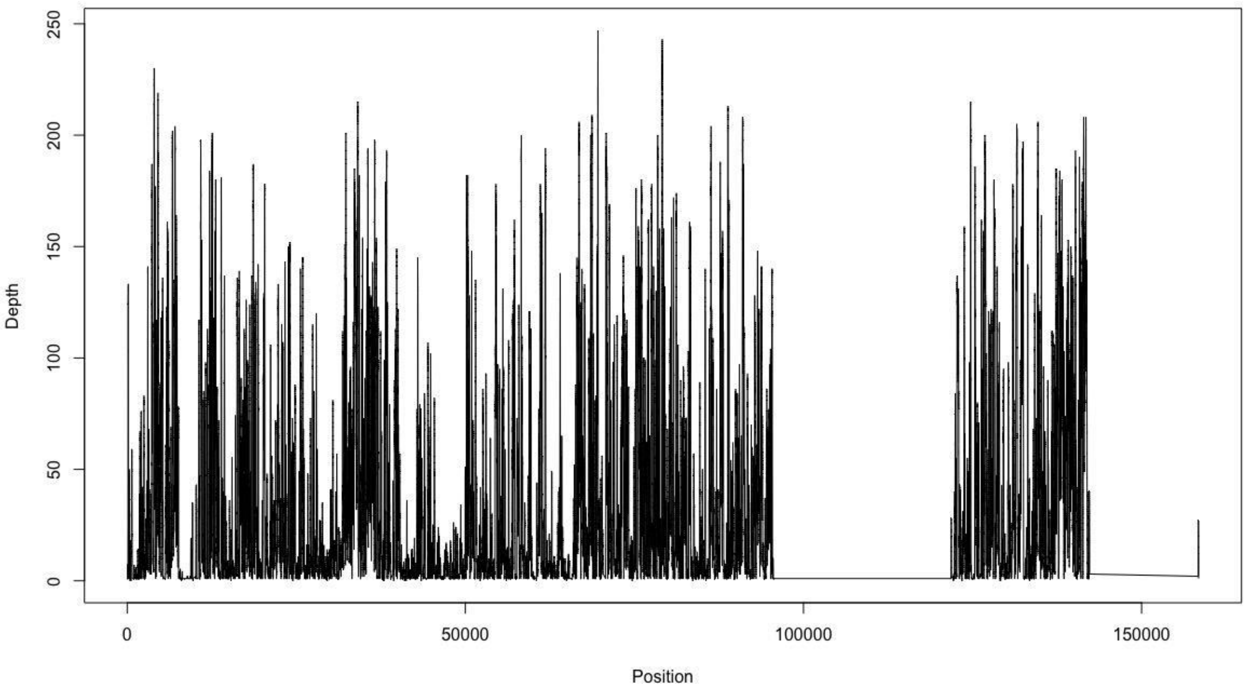

Fig. S6.7.4. Depth of the ancient *Betula* chloroplast reads mapped to the modern chloroplast consensus reference sequence. Coverage across the genome averages 24x but is highly uneven due to the competitive mapping procedure.

All reference genomes used for the phylogenetic placements, downloaded from the Genbank (NCBI) and European nucleotide archive (ENA) repositories, can be found in Source Data S1 Sheet 3.

Table S6.7.1. Table with summary stats of mitochondrial alignments to the key phylogenetically placed taxa.

Mitogenome	Start position	End position	Number of reads	Covered bases	Coverage %	Mean depth	Mean base quality	Mean mapping quality
Anatidae	1	16989	412	5282	31.091	1.343	40.6	35.7
Cervidae	1	21446	129	3511	16.371	0.344	40.7	34.7
Cricetidae	1	17578	209	4313	24.536	0.572	40.7	35.2

Elephantidae	1	17024	182	4774	28.043	0.545	40.7	35.2
Mammut americanum	1	16867	286	6696	39.699	0.963	40.4	36.7
Leporidae	1	16989	412	5282	31.091	1.343	40.6	35.7

6.7.1 General discussion on the phylogenetic placements

Many of the taxa are placed at the base of their respective phylogenies, suggesting that we have identified potential ancestors of the modern Arctic fauna and flora, including reindeers, hares, geese, birch, willow, and poplar. However, phylogenetic placement assumes a sufficient sampling of reference genomes. If we had too few reference samples, a sample might appear to place basally because its close relatives were not included in the phylogeny. However, we find it unlikely that this is the case for many of our samples, which are placed into dense phylogenies with many reference genomes spanning the genus.

6.8 Phylogenetic placement of the *Mammut*

The method for placing the reads classified as Elephantidae are described in the main text and method section, below we report the summary statistics. When mapping reads to the consensus sequence including the sea cow genome (*D. dugon*) for the pathPhynder analysis, we recover a mean read depth of 0.466 and cover 25.4 percent of the mitochondrial genome (4,354bp in total) with 162 reads. The average read length of these mapped reads is 49.33bp. The pathPhynder result showed our sequence falls within the Elephantidae and was most closely related to the mastodons supported by 2 SNPs (transversions) and 5 SNPs (transversions) disagreeing with placement within the other Elephantidae clade (Fig. 3.c.).

This result was further confirmed, when mapping reads to the consensus sequence excluding the sea cow (*D. dugon*) we generated from a single representative from each Elephantidae species. Here, we now recover a mean read depth of 0.545 and cover 28.04 percent of the mitochondrial genome (4,774bp in total) with 182 reads. While the average read length of these mapped reads is 56.79bp.

When mapping exclusively to the consensus sequence of the mastodon (*M. americanum*), generated from all publicly available mastodon sequences, we recovered a mean read depth of 0.963 and covered 39.7 percent of the mitochondrial genome (6,696bp in total) with 286 reads (Fig. S6.8.1.). The average read length of these mapped reads was 56.79bp.

In summary, we find an increase in the number of reads and covered bases, the closer the reads are to the most probable reference species. Hence, we find the reads to be closest to the mastodon references.

To confirm this placement, we created a consensus mitochondrial genome from the reads aligned to the consensus mitochondria genomes of all species within the Elephantidae family including the sea cow (*D. dugon*). We next performed a phylogenetic analysis using BEAST by running our consensus sequence with a panel of Elephantidae. This placed our consensus sequence as sister to American mastodons (*Mammut americanum*) with a high posterior probability of 1 (Fig. S6.8.2.). Within a panel of only mastodon sequences and one representative of the Elephantinae (*Elephas maximus*) used as an outgroup, our consensus sequence is placed basal to all published mastodons with a posterior probability node support of 0.865 (Fig. S6.8.3.).
We lastly sought to date our recovered mastodon mitochondrial genome using molecular age estimation in BEAST using the two different approaches following Karpinski et al (2020) (ref. [92]). When only comparing against previously published mastodon sequences, we find our sequence to have diverged from all other mastodon with a median age 1.37Ma when using the dataset with only radiocarbon dated (Fig. S6.8.4.) and 7.24Ma when using all, molecularly and radiocarbon dated specimens (Fig. S6.8.5.). The median age of our specimen was determined as 1.2Ma (95% HPD: 191,000 – 3.27Ma) when using only the published radiocarbon dated samples and 5.2Ma (95% HPD: 1.64Ma – 10.1Ma) when using all samples. However, both dating methods, with and without the estimated ages of samples >50kya, involve sources of uncertainty. The nature of the radiocarbon dated ancient mastodon data set used to estimate the age of our mitogenome, was highly imbalanced. It has been shown that in such calibrating analyses when using dated, heterochronous tips, tree imbalance can impact precision and produces a bias in which the overall evolutionary timescale is underestimated[146]. When combining radiocarbon and molecular tip dates for age calibration of the sample, tree imbalance decreases due to an increase in taxon sampling representing older lineages but at the same time will add noise to the analysis by introducing more uncertainties around each tip date. It is important to note that even though the analysis with both radiocarbon and molecularly dated tips

may have had more noise introduced, the analysis reached convergence and we are therefore more confident that the data support the result.

The phylogenetic placement of the published mastodon sequences is congruent with those of Karpinski et al. (2020) (ref. [92]) with divergence times broadly overlapping, and the age of our mastodon, although with high uncertainties, falls within the geological, biological and chloroplast molecular age estimates.

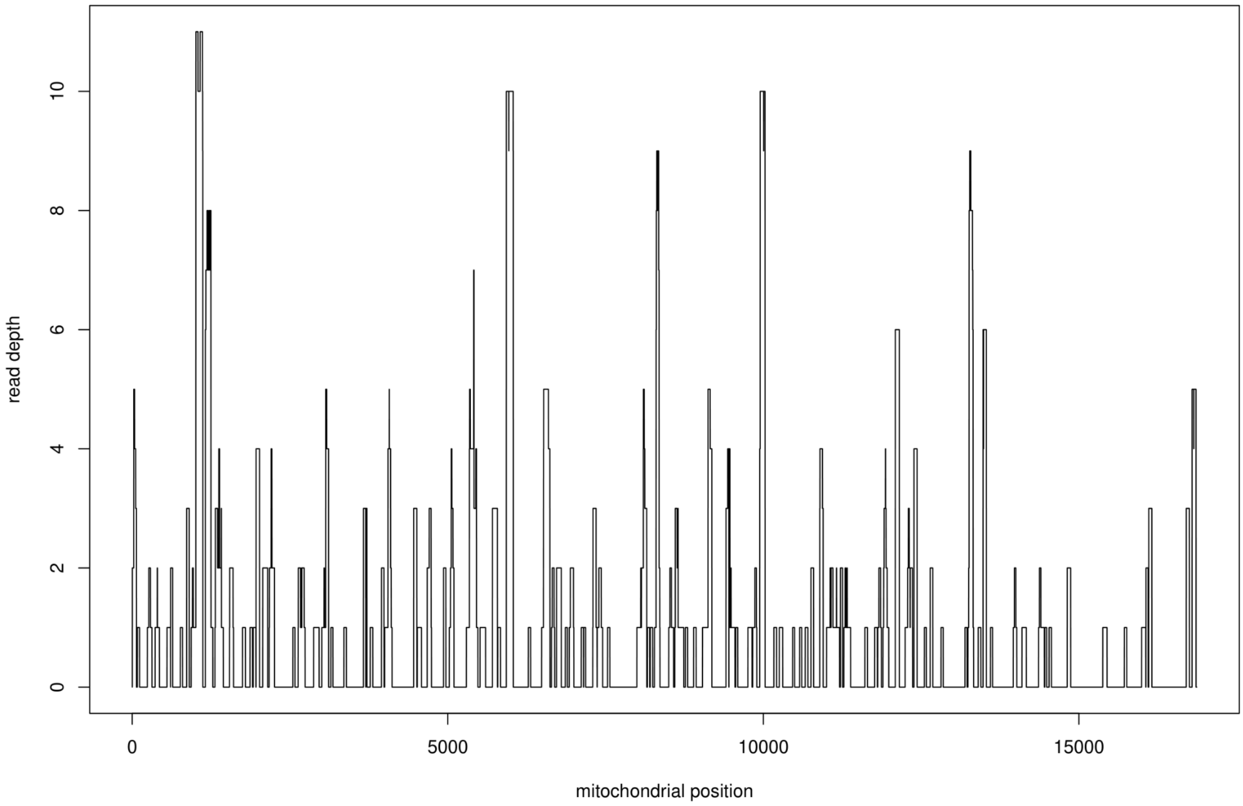

Fig. S6.8.1. Depth and distribution of the 286 reads mapped to *Mammut americanum's* mitochondrial genome.

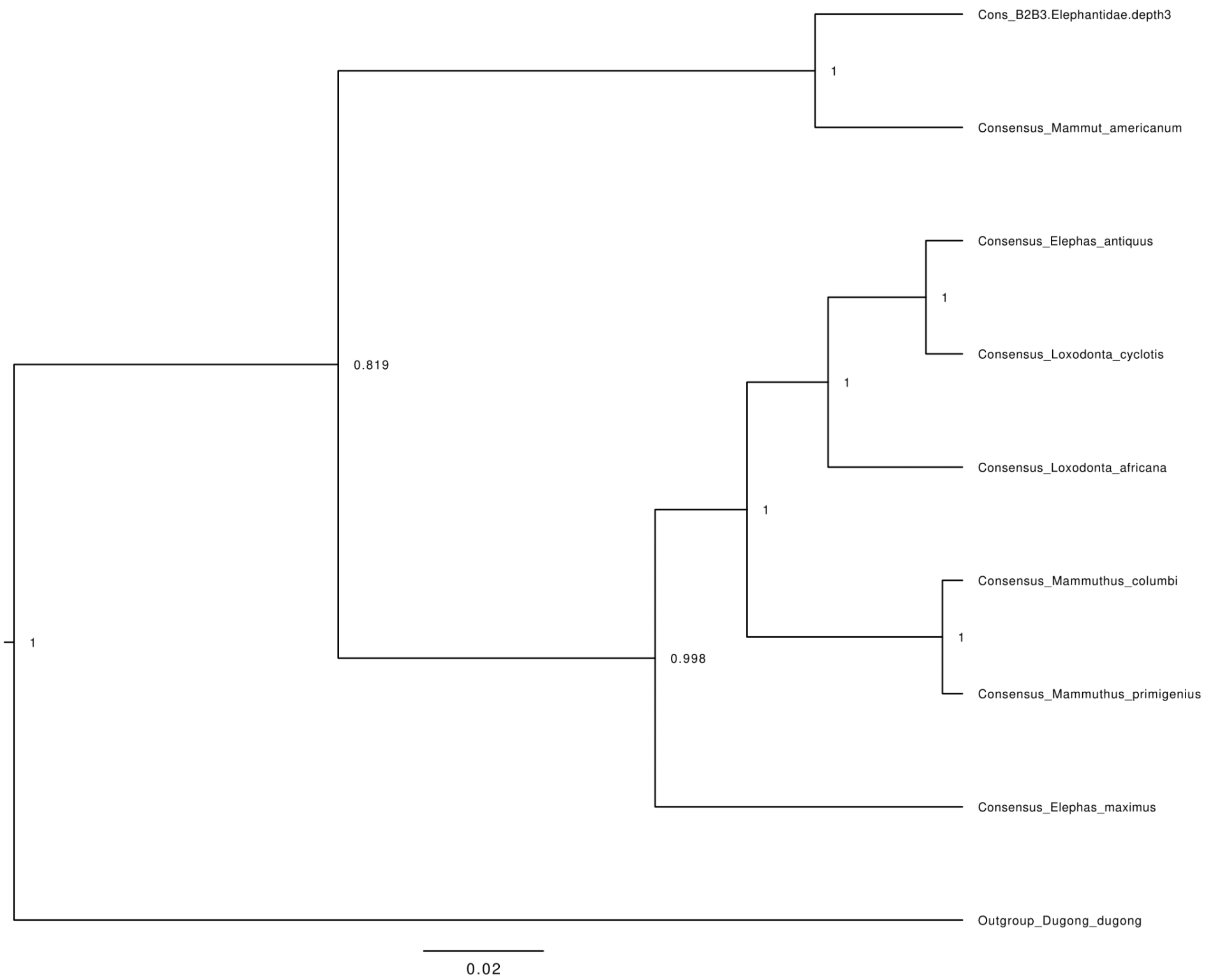

Fig. S6.8.2. BEAST phylogenetic tree inferred using our mastodon sequence and consensus sequences from all available Elephantidae representatives. Numbers on nodes represent posterior probabilities.

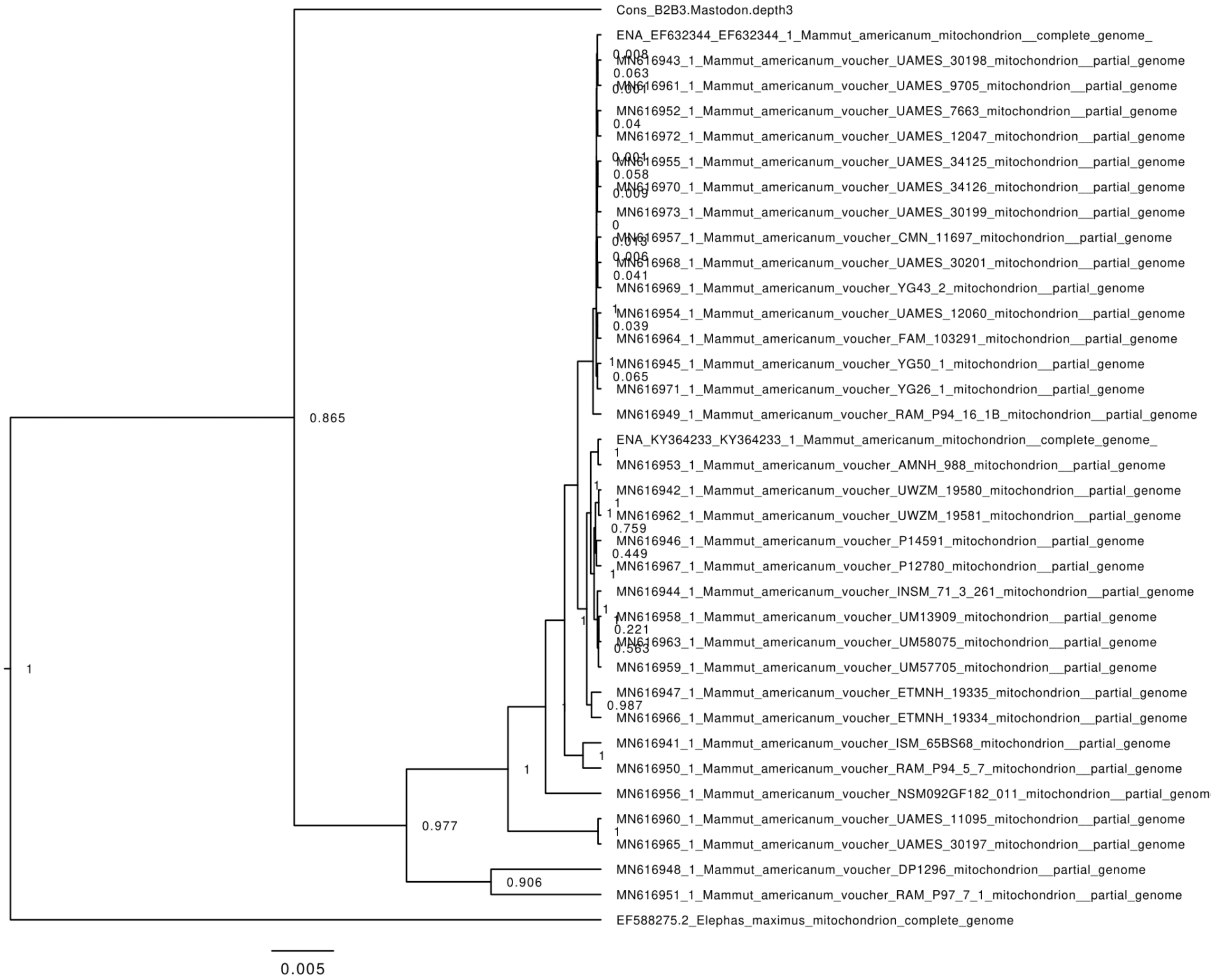

Fig. S6.8.3. BEAST phylogenetic tree inferred using our mastodon mitogenome sequence and all published mastodon mitogenomes. Numbers on nodes represent posterior probabilities.

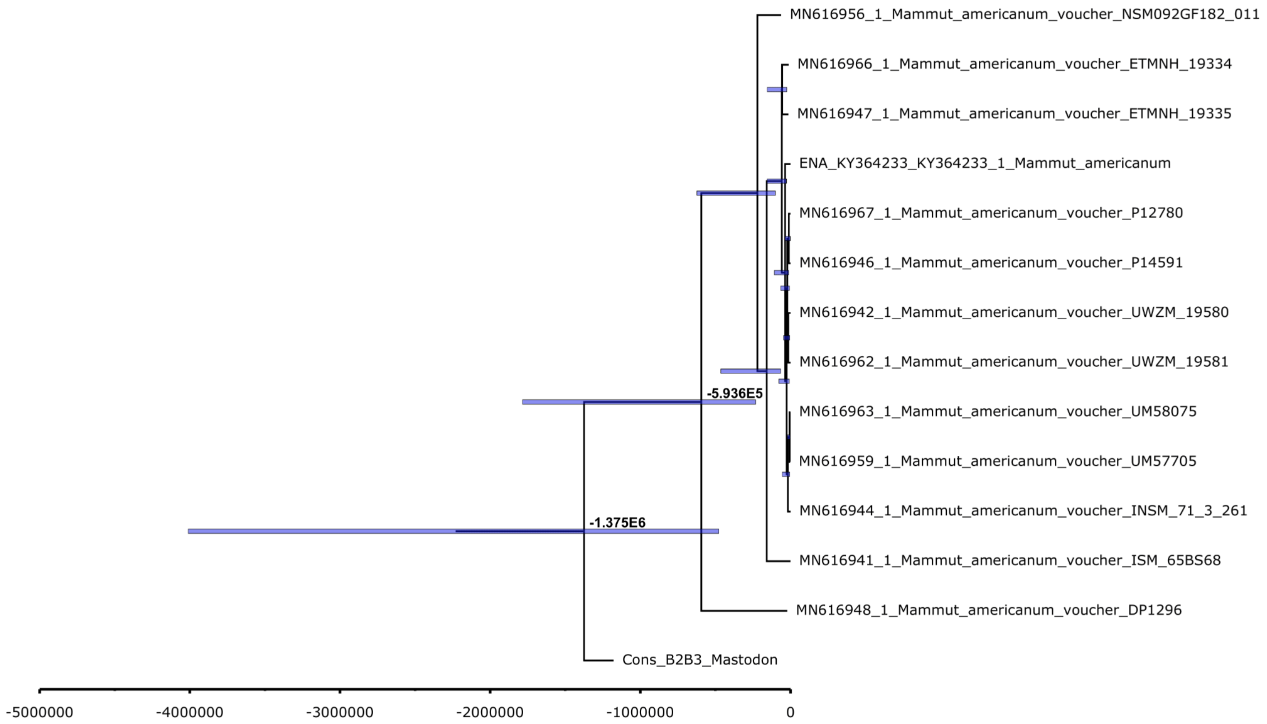

Fig. S6.8.4. Dated BEAST phylogenetic tree inferred using our sequence and only the published radiocarbon dated mastodon sequences. Median posterior ages are given for major nodes in the tree, blue bars represent the 95% HPD.

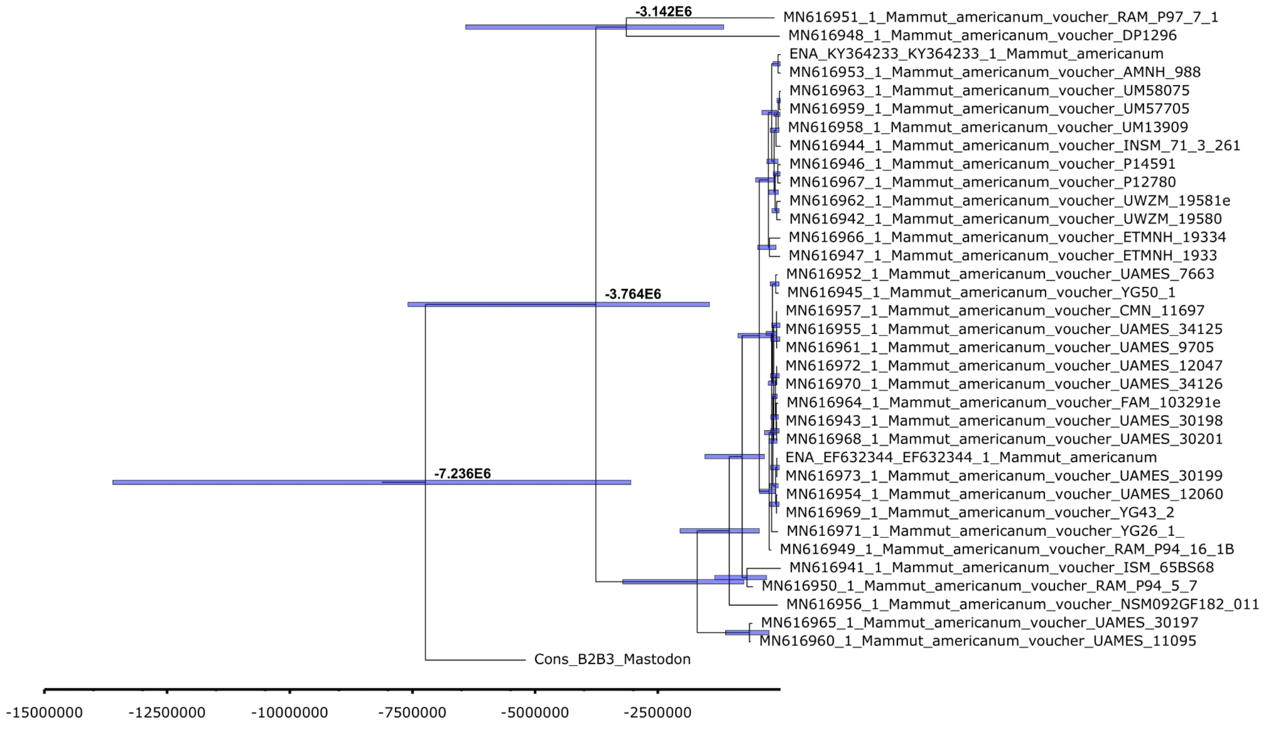

Fig. S6.8.5. Dated BEAST phylogenetic tree inferred using our sequence and all published mastodon mitochondrial genome sequences. Median posterior ages are given for major nodes in the tree, blue bars represent the 95% HPD.

6.9 Plant taxa discussion Plio-Pleistocene DNA, fossil comparison

Our findings from the Kap København Formation show that DNA can be preserved over geological time scales and indicate that successful retrieval of DNA from other Arctic sites in Canada, Alaska, and Siberia is possible, particularly where sample selection may be guided by prior knowledge of mineralogy. Comparison between plant macrofossils and the DNA assemblage has been highly informative at Kap København. It allowed direct confirmation of many genera identified by the DNA analysis, as well as identifying a set of taxa not previously found in the fossil record there, but which have been identified at earlier sites, such as *Saxifraga*, *Populus* and *Chamadaphne* (Fig. 3) dramatically expanding the age and spatial distribution of these ancient floras[55]. Furthermore, macrofossils identified to genera based on morphology can now be associated with phylogenies derived from DNA. The genus *Myrica* identified in the DNA assemblage could be derived from extinct species *M. arctogale* described in the Kap København macrofossil record and older sediments in the region[46,55]. Though these genera did not yield sufficient reads to confirm their ancestral status via phylogenetic placement, the basal position of *Betula* in the assemblage does signal the presence of a potential ancestral genus. In addition to the *Betula* macrofossils at Kap København, well-preserved macrofossils of extinct taxa classified within the Betulaceae[55] and referred to as fossil genus *Tubela* and fossil species *Alnus tertiaria Dorof.* in Takht.[147] have been found at the Fyles Leaf Beds, Ellesmere Island, and other Pliocene fossil-bearing sites in the Canadian High Arctic. Future DNA analysis from these sites might allow phylogenetic assignment of various fossil taxa from different periods. While our data generally support the postulate that the modern arctic flora is derived from a mixture of survivors of the Tertiary forest and elements from high altitudinal sites in Central Asia and North America[148,149], a more precise phylogeny is needed to understand how this evolution occurred.

6.10 DNA taphonomy

Because the topography has been modified by subsequent glaciations, the size and relief of the original catchment cannot be inferred with confidence. However, the taxonomic richness of

individual samples is consistent with an assemblage from a large low-gradient alluvial catchment, consistent with an estuary with multiple tributaries. All but the two least diverse samples contain both aquatic and terrestrial taxa. The most common terrestrial genera *Andromeda, Salix*, *Vaccinium*, *Carex*, *Dryas* and *Equisetum*, dominate the DNA assemblages in almost all samples and are well represented as macrofossils (Source Data S1 - sheet 2). Most of the aquatic taxa are restricted to standing water (e.g., *Hippuris, Stuckenia, Potamogeton, Menyanthes*) or occasionally streams (*Sparganium, Callitriche*). This range of genera combined with the presence of ferns and club mosses is consistent with alluvial deposition from drainage containing a range of microhabitats including well-drained slopes, stream valleys, lakes, coastal plains, and beaches. If the requirements and ecological amplitudes of these genera are generally conserved, the occurrence of *Erica*, *Sphagnum*, *Arctostaphylos* and *Kalmia* would suggest the presence of acidic histosols and snow cover; *Alnus* and perhaps *Populus and Salix* saturated alluvium / riparian habitats; *Artemisia* and *Astragalus* open, primarily mineral soils and *Dryas* cold, open and windswept areas.

A scenario in which eroded sediment from paleosols or permafrost are redeposited after mixing with younger entrained material could produce an assemblage comprising taxa from a succession of communities adapting over millennia to changing conditions. However, we assert that the biotic assemblages of DNA, macrofossils, and pollen from any discrete sample in the Kap København Formation are largely contemporaneous. Redeposited material is unlikely to contribute substantially to the assemblages for several reasons. The macrofossils were generally well preserved, indicative of primary deposition in stable sediments[10]. The fact that eDNA was preserved is also consistent with relatively rapid deposition and burial in a chemically stable and ultimately frozen environment. Multiple episodes of re-entrainment would have allowed the then extracellular DNA to degrade and oxidize and the macrofossils to be fragmented. Furthermore, correspondence between the most abundant macrofossils and eDNA taxa was good in all samples, consistent with a contemporaneous source. Therefore, we conclude that discrete samples are representative of coeval assemblages of plants and animals albeit from a potentially large region with variable habitats.

Conversely, we have generally refrained from interpreting paleoecological change through the sequence because sediment accumulation is chronologically unconstrained and taphonomic processes have been variable. A small group of plants contributes the majority of reads in every sample and occurs in all units, leaving minor taxa to drive the variance through the sequence (Fig. Fig. S6.6.2.). For example, though grass is an important component of Arctic ecosystems that may

be expected across units, we identified 12 genera of Poaceae in unit B3 and B2, while only 7 were identified in B1. Animal assemblages also differ across units - caribou occur only in B1, mastodon and lemmings in B2, and a rich variety in B3, these differences are likely due to the low abundance of animal DNA hence the majority go undetected (Fig. 3). Furthermore, few samples initially tested from Unit B1 yielded DNA and sequence able libraries, and the successful libraries from B1 did not have the same quality (based on appearance of fragment distribution, library concentration, duplication rate and later lower diversity of taxa), which all likely indicates poorer preservation in this unit. B2, which was expected to have the best-preserved DNA by virtue of its mineralogy, had a relatively less diverse DNA assemblage than B3. However, macrofossil incorporation was also variable between units. All these differences might be due to shifts in the deposition environment as well as changes in the drainage over time. Additional and higher resolution sampling and research at the site may provide better controls for these variables allowing more detailed palaeoecological reconstructions in the future.

6.11 Marine eukaryotic profiling.

Table S6.11.1. Taxonomic super groups, Phylum and Class of the detected SMAGs

Super Group	Phylum	Class	# SMAGs	SMAG IDs
Archaeplastida	Chlorophyta	Chloropicophyceae	3	TARA_PSE_93_MAG_00201,TARA_PSW_86_MAG_00260,TARA_PSE_93_MAG_00200
Opisthokonta	Arthropoda	Hexanauplia	4	TARA_MED_95_MAG_00493,TARA_ARC_108_MAG_00265,TARA_PON_109_MAG_00269,TARA_MED_95_MAG_00448
Opisthokonta	Ascomycota	Ascomycetes	1	TARA_PSE_93_MAG_00199
Opisthokonta	Choanozoa	Choanoflagellatea	1	TARA_ARC_108_MAG_00247
Stramenopiles	Bacillariophyta	Bacillariophyceae	2	TARA_ARC_108_MAG_00187,TARA_SOC_28_MAG_00031
Stramenopiles	Bacillariophyta	Mediophyceae	2	TARA_ARC_108_MAG_00108,TARA_SOC_28_MAG_00049
Stramenopiles	MAST-4	New_MAST-4	5	TARA_ARC_108_MAG_00188,TARA_AON_82_MAG_00281,TOSAG00_13,TARA_ARC_108_MAG

				_00220,TARA_ARC_108_MAG_0 0243
Stramenopiles	Ochrophyta	Bolidophyceae	1	TARA_PON_109_MAG_00217
Stramenopiles	Ochrophyta	Chrysophyceae	4	TARA_ARC_108_MAG_00261,TARA_MED_95_MAG_00434,TARA_ARC_108_MAG_00215,TARA_ARC_108_MAG_00234
Stramenopiles	Sister_MAST-4	Sister_MAST-4	1	TARA_SOC_28_MAG_00063

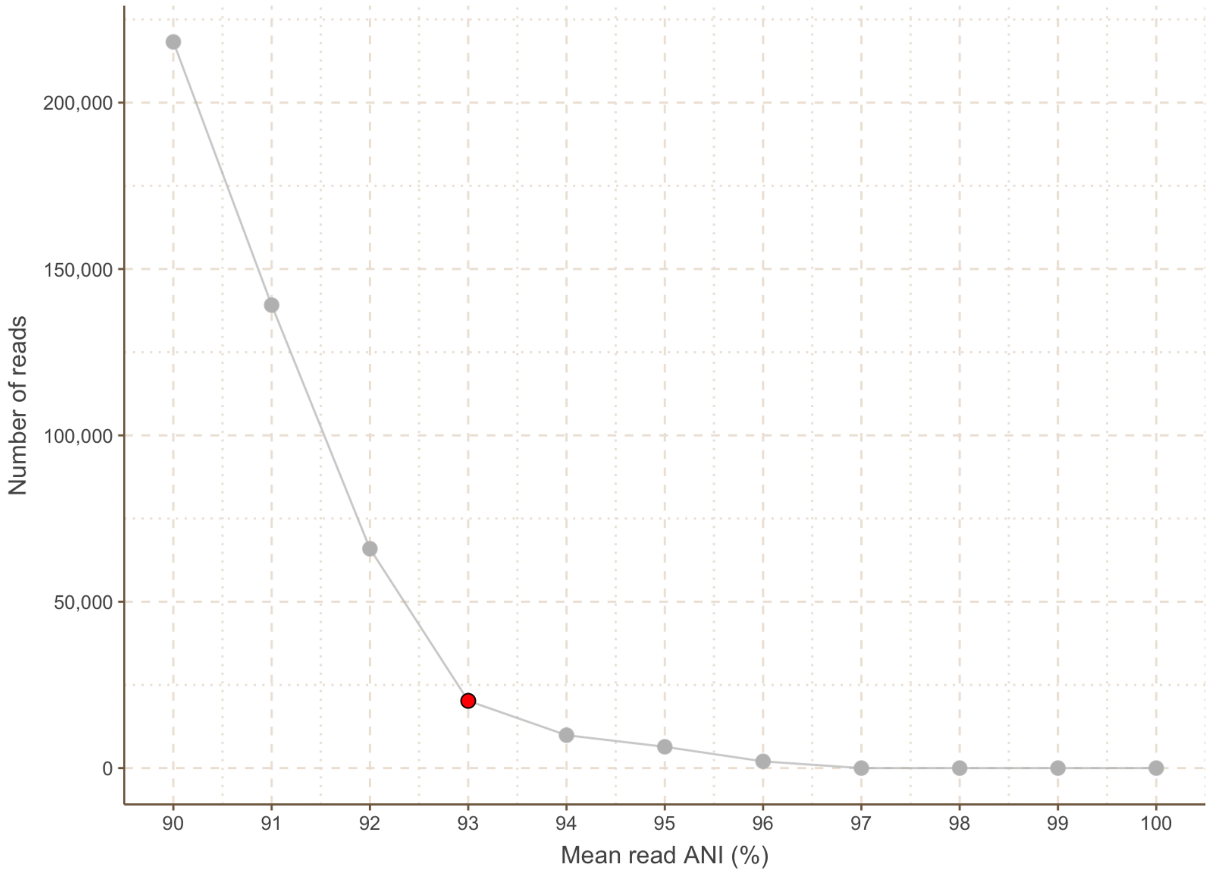

Fig S6.11.1. Identification of the mean read ANI (Average Nucleotide Identity) threshold by the elbow method.

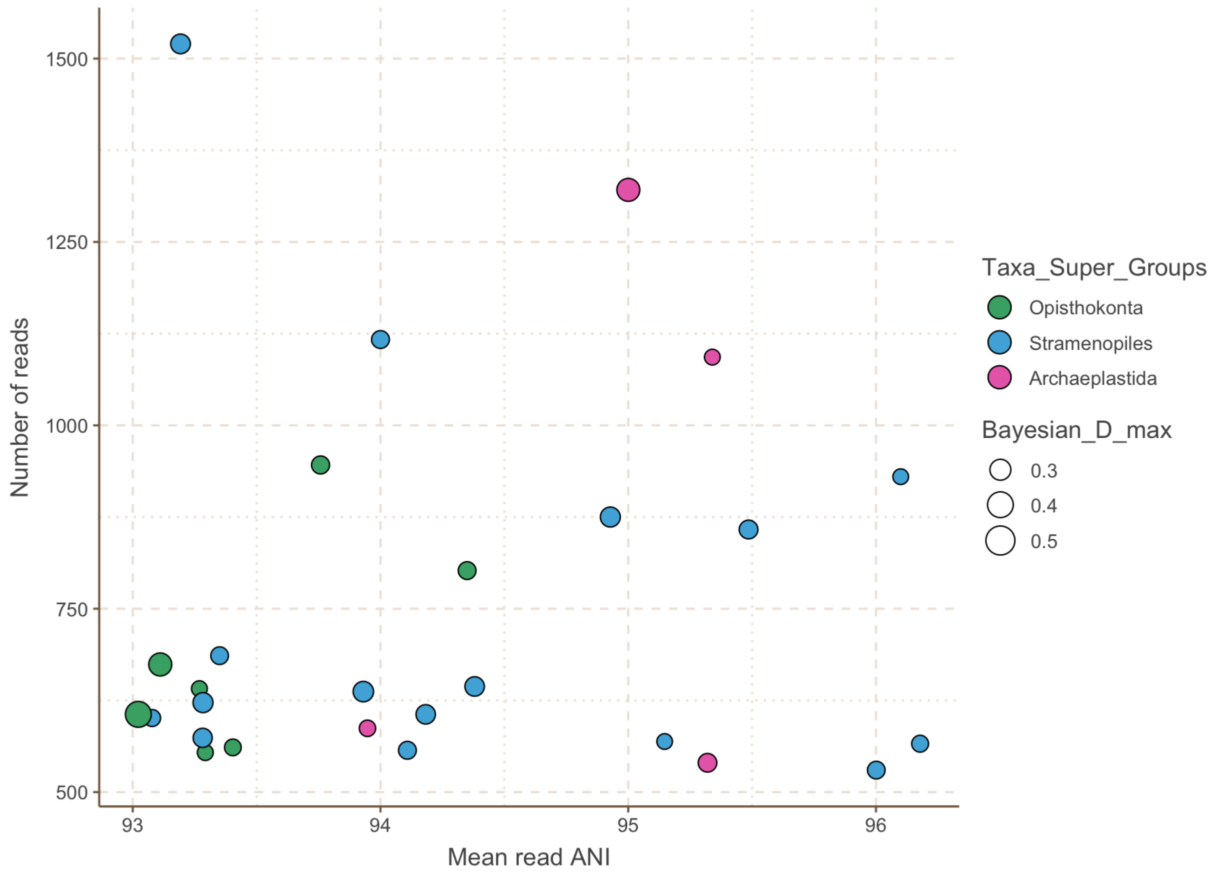

Fig S6.11.2. Mean of the Average Nucleotide Identity (ANI) for the reads mapping to the SMAGs in each sample.

6.12. Droplet digital PCR

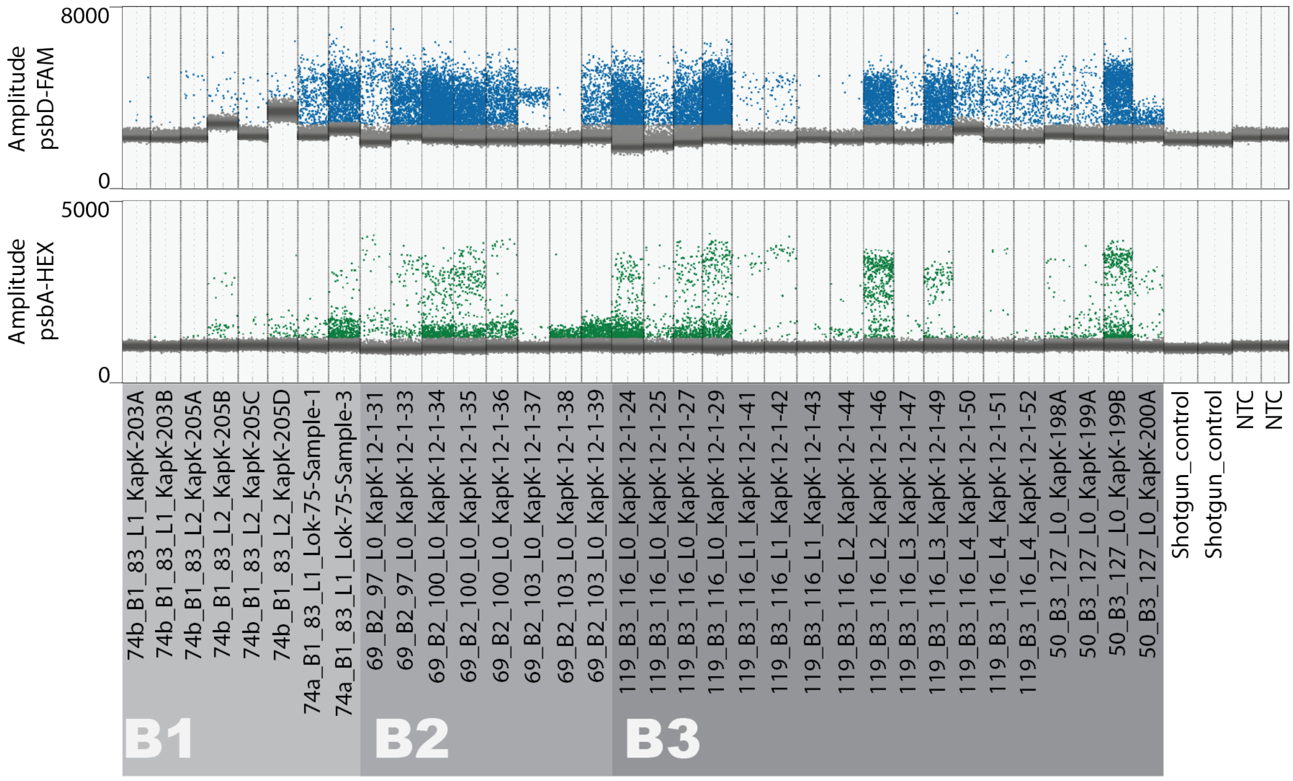

Fig. S6.12.1. Droplet digital PCR (ddPCR) prescreen of plant plastid DNA. Using ddPCR assays targeting Viridiplantae psdD and Poaceae psbA, we screened 34 dual-indexed Illumina libraries. Negative droplets are shown in gray, droplets positive for psbD are blue (upper panel), and droplets positive for psbA are green (lower panel). Two negative library controls and two no-template controls are included in the test. For all samples, the thresholds were set manually. See also Source Data S1 Sheet 4 for ddPCR results.

7. PhyloNorway consortium

Marie Kristine Føreid Merkel[13], Charles Pouchon[17], Adriana Alberti[21], France Denoeud[21], Patrick Wincker[21]

[13]The Arctic University Museum of Norway, UiT – The Arctic University of Norway, NO-9037 Tromsø, Norway

[17]Laboratoire d'Ecologie Alpine (LECA), Université Grenoble Alpes, CNRS, FR-38000 Grenoble, France

[21]Génomique Métabolique, Genoscope, Institut François Jacob, CEA, CNRS, Univ Evry, Université Paris-Saclay, FR-91057 Evry, France

8. References

100. Feyling-Hanssen, R. W. A remarkable foraminiferal assemblage from the Quaternary of northeast Greenland. *Bull. geol. Soc. Denmark,* **38**, 101–107 (1989).

101. Huang, D. I., Hefer, C. A., Kolosova, N., Douglas, C. J. & Cronk, Q. C. B. Whole plastome sequencing reveals deep plastid divergence and cytonuclear discordance between closely related balsam poplars, Populus balsamifera and P. trichocarpa (Salicaceae). *New Phytol.* **204**, 693–703 (2014).

102. Levsen, N. D., Tiffin, P. & Olson, M. S. Pleistocene speciation in the genus Populus (salicaceae). *Syst. Biol.* **61**, 401–412 (2012).

103. Zhang, L., Xi, Z., Wang, M., Guo, X. & Ma, T. Plastome phylogeny and lineage diversification of Salicaceae with focus on poplars and willows. *Ecol. Evol.* **8**, 7817–7823 (2018).

104. *Geologic Time Scale 2020 - 1st Edition*. (Elsevier, 2020).

105. Bennike, O. *et al.* Early Pleistocene sediments on Store Koldewey, northeast Greenland. *Boreas* **44**, 53 (2010).

106. Butler, P. *Paleomagnetism*. (Blackwell Scientific Publications, 1992).

107. Lurcock, P. C. & Wilson, G. S. PuffinPlot: A versatile, user-friendly program for paleomagnetic analysis. *Geochem. Geophys. Geosyst.* **13**, (2012).

108. Feyling-Hanssen, R. W. The biostratigraphic position of the Kap København Formation based upon its foraminifera. *Polar Research* vol. 5 345–346 (1987).

109. Feyling-Hanssen, R. W. Microbiostratigraphy of young Cenozoic marine deposits of the Qivituq Peninsula, Baffin Island. *Marine Micropaleontology* vol. 5 153–184 (1980).

110. Fyles, J. G. *et al.* Geology of Hvitland beds (Late Pliocene), White Point Lowland, Ellesmere Island, Northwest Territories. (1998) doi:10.4095/209577.

111. Símonarson, L. A., Petersen, K. S. & Funder, S. Molluscan palaeontology of the Pliocene-Pleistocene Kap København Formation, North Greenland. (1998).

112. Shackleton, N. J., Hall, M. A. & Pate, D. 15. Pliocene stable isotope stratigraphy of Site 846. in *Proc. Ocean Drill. Program Sci. Results* vol. 138 337–355 (1995).

113. Repenning, C. A. *et al.* The Beringian ancestry of Phenacomys (Rodentia: Cricetidae) and the beginning of the modern arctic ocean borderland Biota. *U.S. Geol. Surv. Bull.* **1687**, 31 pp (1987).

114. Bell, C. J. *et al.* 7. The Blancan, Irvingtonian, and Rancholabrean Mammal Ages. in *Late Cretaceous and Cenozoic Mammals of North America* 232–314 (Columbia University Press, 2004).

115. Repenning, C. A., Weasma, T. R. & Scott, G. R. *The Early Pleistocene (latest Blancan-earliest Irvingtonian) Froman Ferry Fauna and History of the Glenns Ferry Formation, Southwestern Idaho*. (U.S. Government Printing Office, 1995).

116. Woodburne, M. O. *Late Cretaceous and Cenozoic Mammals of North America: Biostratigraphy and Geochronology*. (Columbia University Press, 2004).

117. Janis, K. M. *Evolution of Tertiary Mammals of North America: Volume 1, Terrestrial Carnivores, Ungulates, and Ungulate Like Mammals*. (Cambridge University Press, 1998).

118. Martin, R. A. *et al.* Rodent community change at the Pliocene–Pleistocene transition in southwestern Kansas and identification of the Microtus immigration event on the Central Great Plains. *Palaeogeogr. Palaeoclimatol. Palaeoecol.* **267**, 196–207 (2008).

119. Eidvin, T. & Nagy, J. Foraminiferal biostratigraphy of Pliocene deposits at site 986, Svalbard Margin. in *Proceedings of the Ocean Drilling Program, Scientific Results* vol. 162 3–17 (1999).

120. Jenkins, D. G. *Stratigraphical Atlas of the Fossil Foraminifera*. (Ellis Horwood Limited, 1988).

121. Chmeleff, J., von Blanckenburg, F., Kossert, K. & Jakob, D. Determination of the 10Be half-life by multicollector ICP-MS and liquid scintillation counting. *Nucl. Instrum. Methods Phys. Res. B* **268**, 192–199 (2010).

122. Korschinek, G. *et al.* A new value for the half-life of 10Be by Heavy-Ion Elastic Recoil Detection and liquid scintillation counting. *Nucl. Instrum. Methods Phys. Res. B* **268**, 187–191 (2010).

123. Nishiizumi, K. Preparation of 26Al AMS standards. *Nucl. Instrum. Methods Phys. Res. B*

223-224, 388–392 (2004).

124. Norris, T. L., Gancarz, A. J., Rokop, D. J. & Thomas, K. W. Half-life of26Al. *Journal of Geophysical Research* vol. 88 B331 (1983).

125. Braucher, R., Merchel, S., Borgomano, J. & Bourlès, D. L. Production of cosmogenic radionuclides at great depth: A multi element approach. *Earth Planet. Sci. Lett.* **309**, 1–9 (2011).

126. Borchers, B. *et al.* Geological calibration of spallation production rates in the CRONUS-Earth project. *Quat. Geochronol.* **31**, 188–198 (2016).

127. Granger, D. E. & Muzikar, P. F. Dating sediment burial with in situ-produced cosmogenic nuclides: theory, techniques, and limitations. *Earth Planet. Sci. Lett.* **188**, 269–281 (2001).

128. Lal, D. Cosmic ray labeling of erosion surfaces: in situ nuclide production rates and erosion models. *Earth Planet. Sci. Lett.* **104**, 424–439 (1991).

129. Lifton, N., Sato, T. & Dunai, T. J. Scaling in situ cosmogenic nuclide production rates using analytical approximations to atmospheric cosmic-ray fluxes. *Earth Planet. Sci. Lett.* **386**, 149–160 (2014).

130. Balco, G. Production rate calculations for cosmic-ray-muon-produced 10Be and 26Al benchmarked against geological calibration data. *Quat. Geochronol.* **39**, 150–173 (2017).

131. Gosse, J. C. & Phillips, F. M. Terrestrial in situ cosmogenic nuclides: theory and application. *Quat. Sci. Rev.* **20**, 1475–1560 (2001).

132. Corbett, L. B. *et al.* Cosmogenic 26 Al/ 10 Be surface production ratio in Greenland. *Geophys. Res. Lett.* **44**, 1350–1359 (2017).

133. Nørgaard-Pedersen, N., Mikkelsen, N. & Kristoffersen, Y. Late glacial and Holocene marine records from the Independence Fjord and Wandel Sea regions, North Greenland. *Polar Res.* **27**, 209–221 (2008).

134. Funder, S. *et al.* A 10,000-Year Record of Arctic Ocean Sea-Ice Variability—View from the Beach. *Science* **333**, 747–750 (2011).

135. Kohl, C. P. & Nishiizumi, K. Chemical isolation of quartz for measurement of in-situ -produced cosmogenic nuclides. *Geochim. Cosmochim. Acta* **56**, 3583–3587 (1992).

136. Nishiizumi, K. *et al.* Absolute calibration of 10Be AMS standards. *Nucl. Instrum. Methods Phys. Res. B* **258**, 403–413 (2007).

137. Hidy, A. J., Gosse, J. C., Froese, D. G., Bond, J. D. & Rood, D. H. A latest Pliocene age for the earliest and most extensive Cordilleran Ice Sheet in northwestern Canada. *Quat. Sci. Rev.* **61**, 77–84 (2013).

138. Hidy, A. J., Gosse, J. C., Pederson, J. L., Mattern, J. P. & Finkel, R. C. A geologically constrained Monte Carlo approach to modeling exposure ages from profiles of cosmogenic nuclides: An example from Lees Ferry, Arizona. *Geochem. Geophys. Geosyst.* **11**, (2010).

139. Balco, G. & Rovey, C. W. An isochron method for cosmogenic-nuclide dating of buried soils and sediments. *Am. J. Sci.* **308**, 1083–1114 (2008).

140. Bjerager, M., Alsen, P., Bojesen-Koefoed, J., Kjøller, C., Lindström, S. & Therkelsen, J. *The Triassic of the Wandel Sea Basin and correlation to Svalbard and Western Barents Sea – A summary report*. (2016).

141. Wehmiller, J. F. Amino acid studies of the Del Mar, California, midden site: Apparent rate constants, ground temperature models, and chronological implications. *Earth and Planetary Science Letters* vol. 37 184–196 (1977).

142. Eischeid, J. K., Bruce Baker, C., Karl, T. R. & Diaz, H. F. The Quality Control of Long-Term Climatological Data Using Objective Data Analysis. *J. Appl. Meteorol. Climatol.* **34**, 2787–2795 (1995).

143. Huang, S., Pollack, H. N. & Shen, P. Y. Temperature trends over the past five centuries reconstructed from borehole temperatures. *Nature* **403**, 756–758 (2000).

144. van der Valk, T., Vezzi, F., Ormestad, M., Dalén, L. & Guschanski, K. Index hopping on the Illumina HiseqX platform and its consequences for ancient DNA studies. *Mol. Ecol. Resour.* **20**, 1171–1181 (2020).

145. Palkopoulou, E. *et al.* A comprehensive genomic history of extinct and living elephants. *Proc. Natl. Acad. Sci. U. S. A.* **115**, E2566–E2574 (2018).

146. Duchêne, D., Duchêne, S. & Ho, S. Y. W. Tree imbalance causes a bias in phylogenetic

estimation of evolutionary timescales using heterochronous sequences. *Mol. Ecol. Resour.* **15**, 785–794 (2015).

147. Matthews, J. V., Jr. Plant Macrofossils From the Neogene Beaufort Formation On Banks and Meighen Islands, District of Franklin. *Current Research Part A; Geological Survey of Canada* **87**, 73–87 (1987).

148. Murray, D. F. Causes of Arctic Plant Diversity: Origin and Evolution. in *Arctic and Alpine Biodiversity: Patterns, Causes and Ecosystem Consequences* (eds. Chapin, F. S. & Körner, C.) 21–32 (Springer Berlin Heidelberg, 1995).

149. Abbott, R. J. & Brochmann, C. History and evolution of the arctic flora: in the footsteps of Eric Hultén. *Mol. Ecol.* **12**, 299–313 (2003).
