# Supplementary material for: A 2-million-year-old ecosystem in Greenland uncovered by environmental DNA
Source: Nature. 2022 Dec 7;612(7939):283–91. doi: 10.1038/s41586-022-05453-y (PMC9729109; doi:10.1038/s41586-022-05453-y)
Supplement: Supplementary file 2 — Reporting Summary [file 41586_2022_5453_MOESM2_ESM.pdf]

nature portfolio

Corresponding author(s): Kurt H. Kjær and Eske Willerslev

Last updated by author(s): 01/09/2022

Reporting Summary

Nature Portfolio wishes to improve the reproducibility of the work that we publish. This form provides structure for consistency and transparency in reporting. For further information on Nature Portfolio policies, see our Editorial Policies and the Editorial Policy Checklist.

Statistics

For all statistical analyses, confirm that the following items are present in the figure legend, table legend, main text, or Methods section.

n/a	Confirmed	
☐	☒	The exact sample size (*n*) for each experimental group/condition, given as a discrete number and unit of measurement
☐	☒	A statement on whether measurements were taken from distinct samples or whether the same sample was measured repeatedly
☐	☒	The statistical test(s) used AND whether they are one- or two-sided<br>*Only common tests should be described solely by name; describe more complex techniques in the Methods section.*
☒	☐	A description of all covariates tested
☒	☐	A description of any assumptions or corrections, such as tests of normality and adjustment for multiple comparisons
☐	☒	A full description of the statistical parameters including central tendency (e.g. means) or other basic estimates (e.g. regression coefficient) AND variation (e.g. standard deviation) or associated estimates of uncertainty (e.g. confidence intervals)
☒	☐	For null hypothesis testing, the test statistic (e.g. *F*, *t*, *r*) with confidence intervals, effect sizes, degrees of freedom and *P* value noted<br>*Give P values as exact values whenever suitable.*
☐	☒	For Bayesian analysis, information on the choice of priors and Markov chain Monte Carlo settings
☐	☒	For hierarchical and complex designs, identification of the appropriate level for tests and full reporting of outcomes
☒	☐	Estimates of effect sizes (e.g. Cohen's *d*, Pearson's *r*), indicating how they were calculated

Our web collection on statistics for biologists contains articles on many of the points above.

Software and code

Policy information about availability of computer code

Data collection: No software used for data collection.

Data analysis: Software: BWA (0.7.15), bowtie2 (1.2.3), samtools (1.10), fastq-tools (0.8), sga (0.10.15), gz-sort (1.0), ngsLCA (1.0.0), simka (1.5.3), mafft (7.427), FigTree (1.4.4), biopython (1.79), SNPSites (35), Seqtk-1.3 (r106), PathPhynder, BEAST (1.10.4), metaDMG (0.5.2). BEAST2 (2.6.4), Geneious Prime (2020.0.5), angsd (0.931).

R packages: vegan (2.5-7), ggplot2 (3.3.5), ComplexHeatmap (2.4.3), taxize (0.9.99), IntClust (0.1.0), tidyverse (1.3.1), readxl (1.3.1), reshape2 (1.4.4), lattice (0.20-40), gplots (3.1.1), readr (2.0.1), limma (3.46.0), gghighlight (0.3.2), GGally (2.1.2), Hmisc (4.5-0).

The custom scripts and code are available at https://github.com/miwipe/KapCopenhagen.git

For manuscripts utilizing custom algorithms or software that are central to the research but not yet described in published literature, software must be made available to editors and reviewers. We strongly encourage code deposition in a community repository (e.g. GitHub). See the Nature Portfolio guidelines for submitting code & software for further information.

Data

Policy information about availability of data

All manuscripts must include a data availability statement. This statement should provide the following information, where applicable:

- Accession codes, unique identifiers, or web links for publicly available datasets
- A description of any restrictions on data availability
- For clinical datasets or third party data, please ensure that the statement adheres to our policy

The datasets generated and analysed in this study are included in the paper, in the Extended Data Figures, its Supplementary Information, and the SourceData files 1-5. Raw sequence data is available through the ENA project accession PRJEB55522. Pollen counts are available through https://github.com/miwipe/KapCopenhagen.git.

Human research participants

Policy information about studies involving human research participants and Sex and Gender in Research.

Reporting on sex and gender	*Use the terms sex (biological attribute) and gender (shaped by social and cultural circumstances) carefully in order to avoid confusing both terms. Indicate if findings apply to only one sex or gender; describe whether sex and gender were considered in study design whether sex and/or gender was determined based on self-reporting or assigned and methods used. Provide in the source data disaggregated sex and gender data where this information has been collected, and consent has been obtained for sharing of individual-level data; provide overall numbers in this Reporting Summary. Please state if this information has not been collected. Report sex- and gender-based analyses where performed, justify reasons for lack of sex- and gender-based analysis.*
Population characteristics	*Describe the covariate-relevant population characteristics of the human research participants (e.g. age, genotypic information, past and current diagnosis and treatment categories). If you filled out the behavioural & social sciences study design questions and have nothing to add here, write "See above."*
Recruitment	*Describe how participants were recruited. Outline any potential self-selection bias or other biases that may be present and how these are likely to impact results.*
Ethics oversight	*Identify the organization(s) that approved the study protocol.*

Note that full information on the approval of the study protocol must also be provided in the manuscript.

Field-specific reporting

Please select the one below that is the best fit for your research. If you are not sure, read the appropriate sections before making your selection.

☐ Life sciences ☐ Behavioural & social sciences ☒ Ecological, evolutionary & environmental sciences

For a reference copy of the document with all sections, see nature.com/documents/nr-reporting-summary-flat.pdf

Ecological, evolutionary & environmental sciences study design

All studies must disclose on these points even when the disclosure is negative.

Study description	We shotgun sequenced ancient environmental DNA from sediments found at the geological formation Kap København in Greenland, for paleo-environmental recontruction.
Research sample	41 samples obtained from bulk samples or directly in the profiles were used for DNA analysis. 8 1kg bulk samples were obtained for Cosmogenic nuclide burial dating. In addition, different types of minerals were used to test DNA adsorption and release. Sixty-nine samples were collected for determination of the polarity. All samples were taken during three field trips, and spanning 5 different localities within the same formation.
Sampling strategy	Samples were taken across the three units and from 5 different sites, within each site biological replicates were taken in the units both horizontally and vertically see DNA metadata.
Data collection	DNA processing was performed at Centre for GeoGenetics and sequenced at the Danish National Sequencing Centre on Illumina platforms (HiSeq 4000, NovaSeq6000).
Timing and spatial scale	DNA Data were collected from sediment samples from Kap København formation, the northern most Greenland which has been date to 2.0 Mya.
Data exclusions	The DNA results only includes samples that yielded sequenceable DNA. Some samples did not.

Reproducibility	The strongest evidence for reproducibility is that this study includes replicates of geological layers from the same unit but at different locations within the formation (sites) and the fact that they yield highly identical taxonomic profiles. Further, we had biological replicates within the same site and unit, as well as technical replicates of individual samples. All yielding near to identical results.
Randomization	Randomization is not relevant.
Blinding	Blinding is not relevant, as there is no presupposed hypothesis.

Did the study involve field work? ☒ Yes ☐ No

Field work, collection and transport

Field conditions	Field works were performed by three different expedition groups. Details are supplied in Methods and SI.
Location	Kap København Formation in North Greenland (82° 24' 00" N 22° 12' 00" W)
Access & import/export	Sediment samples were collected and exported by different research groups from different countries, in agreement with the rules of the specific countries. All sediment samples were imported to Denmark as geological sediment samples for research, for which there is no specific permit required by the authorities.
Disturbance	The samples concerns small sediment samples, and didn't cause disturbance to the surrounding environment as a whole.

Reporting for specific materials, systems and methods

We require information from authors about some types of materials, experimental systems and methods used in many studies. Here, indicate whether each material, system or method listed is relevant to your study. If you are not sure if a list item applies to your research, read the appropriate section before selecting a response.

Materials & experimental systems

n/a	Involved in the study	
☒	☐	Antibodies
☒	☐	Eukaryotic cell lines
☒	☐	Palaeontology and archaeology
☒	☐	Animals and other organisms
☒	☐	Clinical data
☒	☐	Dual use research of concern

Methods

n/a	Involved in the study	
☒	☐	ChIP-seq
☒	☐	Flow cytometry
☒	☐	MRI-based neuroimaging
